# YY1 regulates vascular resistance and blood pressure dynamics through epigenetic control of m^6^A RNA modifications in vascular smooth muscle cells

**DOI:** 10.1093/cvr/cvaf136

**Published:** 2025-08-07

**Authors:** Wenchu Ye, Wentao Gao, Cheng Kiu Ho, Lei Cui, James Y W Lau, Xiao Yu Tian, Bin Zhou, Kathy O Lui

**Affiliations:** CAS CEMCS-CUHK Joint Laboratory for Cardiovascular Sciences, Department of Chemical Pathology, and Li Ka Shing Institute of Health Science, The Chinese University of Hong Kong, Room 306D, Li Ka Shing Medical Sciences Building, Prince of Wales Hospital, Hong Kong, China; CAS CEMCS-CUHK Joint Laboratory for Cardiovascular Sciences, Department of Chemical Pathology, and Li Ka Shing Institute of Health Science, The Chinese University of Hong Kong, Room 306D, Li Ka Shing Medical Sciences Building, Prince of Wales Hospital, Hong Kong, China; Department of Surgery, Prince of Wales Hospital, The Chinese University of Hong Kong, Hong Kong, China; CAS CEMCS-CUHK Joint Laboratory for Cardiovascular Sciences, Department of Chemical Pathology, and Li Ka Shing Institute of Health Science, The Chinese University of Hong Kong, Room 306D, Li Ka Shing Medical Sciences Building, Prince of Wales Hospital, Hong Kong, China; CAS CEMCS-CUHK Joint Laboratory for Cardiovascular Sciences, Department of Chemical Pathology, and Li Ka Shing Institute of Health Science, The Chinese University of Hong Kong, Room 306D, Li Ka Shing Medical Sciences Building, Prince of Wales Hospital, Hong Kong, China; Department of Surgery, Prince of Wales Hospital, The Chinese University of Hong Kong, Hong Kong, China; School of Biomedical Sciences, The Chinese University of Hong Kong, Hong Kong, China; CAS CEMCS-CUHK Joint Laboratory for Cardiovascular Sciences, New Cornerstone Investigator Institute, State Key Laboratory of Cell Biology, Shanghai Institute of Biochemistry and Cell Biology, Center for Excellence in Molecular Cell Science, Chinese Academy of Sciences, Shanghai, China; CAS CEMCS-CUHK Joint Laboratory for Cardiovascular Sciences, Department of Chemical Pathology, and Li Ka Shing Institute of Health Science, The Chinese University of Hong Kong, Room 306D, Li Ka Shing Medical Sciences Building, Prince of Wales Hospital, Hong Kong, China

**Keywords:** Smooth muscle contraction, Vascular resistance, Blood pressure, Hypertension, YY1, Mettl3, m^6^A

## Abstract

**Aims:**

Recent genome-wide association study analysis has identified YY1 as a novel locus associated with blood pressure traits; however, whether YY1 directly controls vasoreactivity remains unknown. The principal function of vascular smooth muscle cells (VSMCs) is to contract, which is essential for regulating vascular tone, blood flow, and blood pressure. We hypothesized that YY1, a transcription factor, facilitates vascular function by epigenetically regulating gene expression in VSMCs.

**Methods and results:**

The effects of VSMC-specific YY1 loss were studied in mice. Lineage tracing, calcium imaging, and wire myography were performed to assess vasoreactivity. Genome-wide analysis through RNA-seq, ChIP-seq, m^6^A-seq, RNA immunoprecipitation, and transcript stability assays were conducted to evaluate gene expression and regulation. Co-immunoprecipitation was performed to study interactions between YY1 and chromatin regulators. AAV-mediated SM22-specific gene delivery was used to rescue vascular function *in vivo*. Contractile VSMCs were differentiated from human embryonic stem cells for *in vitro* experiments. Hypertension was induced *in vivo* using salt and L-NAME treatments. We demonstrate that vascular contraction and blood pressure are significantly reduced in *Myh11^CreER^;Yy1^fl/fl^* mice. YY1 does not regulate VSMC proliferation, survival, calcium entry, or membrane polarization in homeostasis. Integrative analyses of transcriptomics, epitranscriptomics, and epigenetics identified *Mettl3* as a putative downstream target of YY1. Like YY1 loss-of-function, impaired vascular contraction and reduced blood pressure were observed in *Myh11^CreER^;Mettl3^fl/fl^* mice. *Mylk2*, *Tgfb2*, and *Myh11* were significantly down-regulated after genetic ablation of *Yy1* or *Mettl3* in VSMCs. Further analysis showed that Mettl3-mediated m^6^A mRNA methylation stabilizes the transcripts of these genes, possibly through the m^6^A reader IGF2BP1. AAV-mediated, VSMC-specific *Mettl3* gene delivery significantly improved vascular contractility in *Yy1*-deficient mice, functionally confirming *Mettl3* as a direct downstream target of YY1. Mechanistically, YY1 binds to the *Mettl3* promoter near regions of H3K4 trimethylation and activates *Mettl3* transcription by recruiting Set1A-Wdr82 complex for H3K4me3 deposition. Both *Myh11^CreER^;Yy1^fl/fl^* and *Myh11^CreER^;Mettl3^fl/fl^* mice exhibited delayed onset of hypertension.

**Conclusion:**

YY1 maintains vascular contraction and regulates blood pressure by stabilizing *Mylk2*, *Tgfb2*, and *Myh11* transcripts through the activation of *Mettl3* transcription in VSMCs. These findings provide novel insights into the epigenetic control of VSMC epitranscriptomes and unravel a new mechanism underlying VSMC-mediated vasoconstriction through the YY1/Mettl3 regulatory axis. Additionally, our results demonstrate a clinically relevant role for the YY1/Mettl3 axis in mitigating hypertension and regulating blood pressure under both normal and hypertensive conditions.

## Graphical Abstract

**Graphical Abstract:**
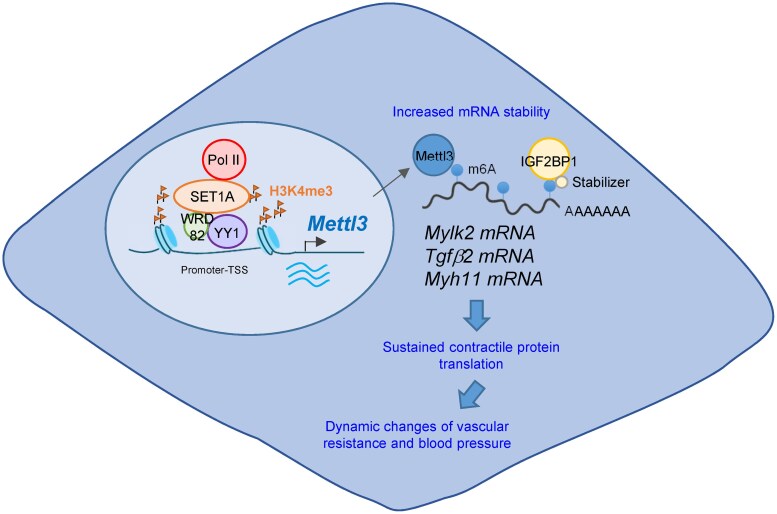

**Time of primary review: 31 days**

**See the editorial comment for this article ‘Epigenetic leash on the epitranscriptome: unveiling a novel regulatory axis in vascular smooth muscle contractility’, by J. Huang and Y. Fu, https://doi.org/10.1093/cvr/cvaf162.**

## 1. Introduction

In the vasculature, the contractility of the vascular smooth muscle cell (VSMC) layer is a primary determinant of blood pressure, which, in turn, controls blood flow to deliver oxygen and nutrients to all organ systems of the body. VSMC contraction is initiated by a membrane potential-dependent influx of calcium (Ca^2+^) through tightly orchestrated Ca^2+^ and potassium (K^+^) channels (for review, see Hill-Eubanks *et al*., Jackson, and Sanders and Koh^1–3^ ). The increased intracellular Ca^2+^ concentration promotes the formation of a Ca^2+^-calmodulin complex.^4^ This complex binds to and activates the enzymatic activity of myosin light chain kinase (MLCK), which phosphorylates the myosin regulatory light chain (RLC). Phosphorylation of RLC activates myosin ATPase, enabling its binding to actin for cross-bridge formation and muscle contraction. On the other hand, dephosphorylation of RLC by myosin light chain phosphatase (MLCP) favours relaxation.^5^ Therefore, a balance between MLCK and MLCP activities is required to control the sliding of actin filaments and maintain VSMC vasoreactivity. In addition to their role in the contractile apparatus, VSMCs produce complex extracellular matrix (ECM) proteins that define the mechanical strength and elasticity of the vessel.^6^ In response to increased mechanical strain, human VSMCs have been demonstrated to produce elevated levels of ECM proteins, including collagen and fibronectin, mediated by TGFβ signalling.^7^ Importantly, VSMCs exhibit remarkable plasticity, allowing them to switch from a contractile phenotype to a synthetic phenotype during vascular remodelling in disease or after injury. While recent studies have elegantly demonstrated the epigenetic control of VSMC plasticity in various vascular diseases,^8,9^ the epigenetic regulation of the principal contractile function of VSMCs remains poorly understood.

Transcription factors often reshape the epigenetic landscape to regulate gene expression during development and disease. In particular, they can directly influence the establishment and maintenance of epigenetic marks on DNA. Yin Yang 1 (YY1), a DNA-binding zinc finger transcription factor, functions as both a transcriptional activator and repressor to regulate networks of genes required for cellular processes during the development of the brain,^10^ heart,^11^ pancreas,^12^ immune cells,^13^ and even tumour cells.^14^ Mechanistically, our previous work has demonstrated that YY1 stabilizes promoter–enhancer looping to facilitate gene transcription through directly interacting with RNA polymerase II.^12^ YY1 also epigenetically regulates gene expression by recruiting chromatin modifiers. For instance, it acts as a transcriptional activator by interacting with the histone acetyltransferase p300, which promotes the formation of open and active chromatin.^15^ Conversely, YY1 can serve as a repressor by interacting with the histone deacetylase RPD3^16^ or by recruiting the polycomb methyltransferase EZH2 to mediate trimethylation of histone (H) 3 lysine (K) 27 (H3K27me3).^17^ Although YY1 is ubiquitously expressed in mammalian cells, its pathophysiological functions in many cell types, including VSMCs, remain poorly understood. Studies have reported that YY1 inhibits the proliferation of rat and rabbit VSMCs *in vitro*.^18–20^ Specifically, YY1 represses p21^WAF1/Cip1^ transcription, blocking Rb phosphorylation by perturbing the assembly of the p21^WAF1/Cip1^/Cdk4/Cyclin D1 complex.^18^ As a result, adenovirus-mediated overexpression of full-length^18^ or truncated^19^ YY1 reduces neointima formation 14 days after balloon catheter injury in rats. These studies highlight a potential therapeutic role of YY1 in combating vascular proliferative diseases. Nevertheless, the physiological function of YY1 in VSMCs remains largely unknown due to the lack of *in vivo* genetic evidence. This gap underscores the need for further investigation to understand YY1’s role in the regulation of VSMC function under physiological and pathological conditions.

Recent genome-wide association studies involving over a million individuals have identified YY1 as one of the new loci associated with blood pressure traits.^21^ We hypothesized that YY1 may facilitate vasoreactivity through epigenetically regulating gene expression in VSMCs. In this study, we demonstrate that YY1 loss-of-function results in significantly impaired vascular contraction, independent of membrane depolarization and Ca^2+^ entry in VSMCs. Mechanistically, we describe how YY1 directly binds to the *Mettl3* promoter, adjacent to regions of H3K4 trimethylation, and activates *Mettl3* transcription by recruiting the Set1A-Wdr82 complex for H3K4me3 deposition. The YY1/Mettl3 regulatory axis is essential for maintaining the stability of *Mylk2*, *Tgfb2*, and *Myh11* transcripts through m^6^A mRNA methylation. These findings provide novel insights into the epigenetic control of VSMC epitranscriptomes and unravel a new mechanism underlying VSMC-mediated vasoconstriction.

## 2. Methods

The data and analytical methods that support the findings of this study are available from the corresponding author on reasonable request. An expanded experimental procedures section is provided in the Supplementary material.

### 2.1 Mice

The care and use of mice were in accordance with the US NIH guidelines and approval by the CUHK Animal Experimentation Ethics Committee. The mouse lines *Myh11-CreER*,^22^  *R26-YFP*,^23^  *YY1-flox*,^12^ and *Mettl3-flox*^24^ were reported previously by us. As *Myh11-CreER* is Y-linked, only male mice were used in this study. To induce Cre recombination activation, tamoxifen (Tam, MedChemExpress, cat no. HY-13757A) was dissolved in corn oil (10 mg/mL) and five doses with each at 100 µg/g body weight were given to 6–12 weeks old males for five consecutive days through intraperitoneal injection. The duration following the last dose of Tam administration is indicated where appropriate. To record cell proliferation *in vivo*, mice were intraperitoneally injected with EdU at a dosage of 10 mM per 20 gram body weight. To rescue Mettl3 expression *in vivo*, mice were intravenously injected with AAV serotype 9 vector containing *Mettl3* or ZsGreen (control) driven under the control of a *Tagln* (encoding SM22α) promoter. Mice were euthanized by performing cervical dislocation under anaesthesia with 2.5% isoflurane.

### 2.2 Hypertensive model and blood pressure measurement

Male littermate mice of each genotype, aged at 8–12 weeks, were treated with L-NAME (N^G^-nitro-L-arginine methyl ester, 0.5 mg/mL, Sigma) and NaCl (0.9%) in drinking water for 42 days to induce hypertension, as previously described.^25^ Blood pressure was measured at the indicated time points using the tail-cuff plethysmograph method with the CODA™ non-invasive blood pressure system (Kent Scientific, Torrington, CT, USA). Briefly, the experiments were conducted in a designated quiet area, and mice were acclimatized for a 1 h period before the experiments commenced. The patency of the occlusion and volume pressure recording (VPR) cuffs was routinely checked before the start of each experiment. Mice were encouraged to walk into the restraint tubes and were secured with adjustable end holders to minimize excessive movement. The occlusion cuff was placed at the base of the tail, while the VPR sensor cuff was positioned adjacent to the occlusion cuff. Heating pads were preheated to 33–35°C, and mice were warmed for 5–10 min prior to blood pressure recordings. To measure blood pressure, the occlusion cuff was inflated to 250 mmHg and then deflated over a 15 s period. The VPR sensor cuff detected changes in tail volume as blood returned to the tail during occlusion cuff deflation. The minimum volume change was set at 15 μL. Each recording session consisted of 15–25 inflation and deflation cycles per set, with the first five cycles designated as ‘acclimation’ cycles (not included in the analysis). The subsequent cycles were used for data collection and analysis.

### 2.3 Resource availability

All sequencing data have been made publicly available at the NCBI BioProject. The data for bulk RNA-seq are available through PRJNA1279010, YY1 ChIP-seq through PRJNA1279011; and me-RIP-seq through PRJNA1278981. Previously published VSMC histone ChIP-seq datasets were retrieved from NCBI GEO through GSE96206. The computer code used in this study is available on request.

### 2.4 Statistical analysis

All data are expressed as MEAN ± SEM, with at least *n* = 6 biological replicates performed under the same conditions unless otherwise indicated. Statistical analysis for comparisons between two groups was performed using a two-sided, unpaired Student’s *t*-test. For comparisons involving more than two groups, data were analysed using ANOVA followed by Tukey’s method for multiple comparisons. Time–response relationships were analysed by calculating the area under curve, followed by Student’s *t*-tests, or by one-way ANOVA with Tukey’s multiple comparisons. Blood pressure changes were analysed using two-way repeated measures ANOVA with Sidak’s multiple comparisons. Statistical significance was accepted at *P* < 0.05, with actual *P* values reported where appropriate.

## 3. Results

### 3.1 VSMC YY1 controls vascular contraction *in vivo*

To investigate the functional role of YY1 in VSMCs, we generated a VSMC lineage-specific *Yy1* knockout mouse model, *Myh11^CreER^;Yy1^fl/fl^*, by crossing *Myh11^CreER^* with *Yy1^fl/fl^* (referred to hereafter as VSMC-YY1^cKO^). *Myh11^CreER^* (referred to as VSMC-YY1^WT^) was used as a control. Additionally, we crossed VSMC-YY1^cKO^ and VSMC-YY1^WT^ with *R26-loxP-Stop-loxP-YFP* (*R26^YFP/+^*) to genetically trace VSMCs *in vivo* (see Supplementary material online, *Figure S1A*). To determine whether YY1 regulates VSMC survival, we examined the quantity, proliferation, and apoptosis of VSMCs after *Yy1* ablation. Flow cytometric analysis showed no significant difference in %YFP^+^ cells among all aortic cells derived from VSMC-YY1^WT^;R26^YFP/YFP^ and VSMC-YY1^cKO^;R26^YFP/YFP^ mice at 4 weeks after Tam administration (see Supplementary material online, *Figure S1B*). To further examine if YY1 regulates VSMC proliferation, we injected EdU intraperitoneally to VSMC-YY1^WT^;R26^YFP/YFP^ and VSMC-YY1^cKO^;R26^YFP/YFP^ mice at 2 weeks after Tam administration. Aortas were harvested 16 h after EdU incorporation for flow cytometric analysis (see Supplementary material online, *Figure S1C*). Very few EdU^+^YFP^+^ cells were found among the total YFP^+^ cells in both groups, and there was no significant difference in the turnover rate of VSMCs between the two groups (see Supplementary material online, *Figure S1D*), confirming that adult VSMCs exhibit minimal proliferation even after *Yy1* depletion. Furthermore, immunostaining of frozen aortic sections from VSMC-YY1^cKO^ and VSMC-YY1^WT^ mice revealed no difference in the number of cCASP3 (cleaved caspase 3) ^+^ α-SMA^+^ apoptotic VSMCs at 8 weeks after Tam administration (see Supplementary material online, *Figure S1E*). These results indicate that YY1 does not regulate VSMC proliferation or survival under homeostatic conditions.

**Figure 1:**
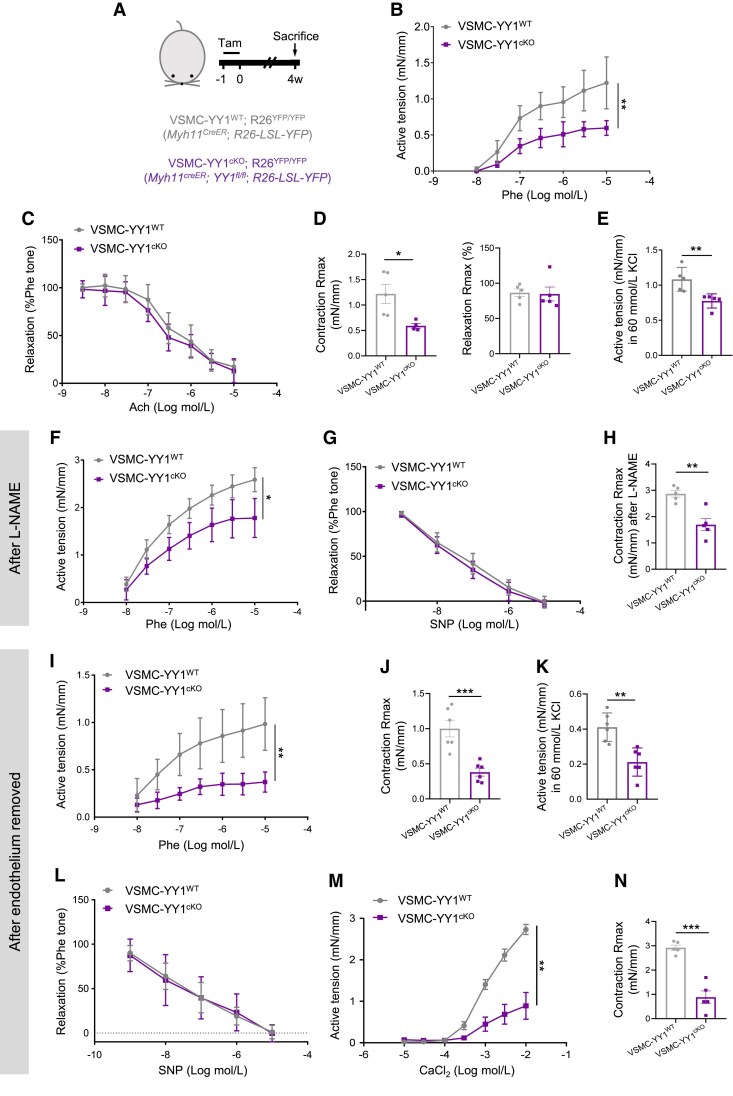
Loss of YY1 in VSMCs impairs vascular contraction *in vivo.* (*A*) A schematic showing the experimental design and the generation of Myh11-CreER (VSMC-YY1^WT^);R26^YFP/YFP^ and Myh11-CreER;Yy1^fl/fl^ (VSMC-YY1^cKO^);R26^YFP/YFP^ mice. (*B*) Concentration-dependent vasoconstriction responses of mouse aortas to Phe and (*C*) concentration-dependent relaxation responses to Ach were measured in VSMC-YY1^cKO^ and VSMC-YY1^WT^ aortas 28 after Tam treatment, five vessels per group. (*D* and *E*) The maximum responses (Rmax) in vasoconstrictions and relaxations of the aortas (*D*) and 60 mM KCl-mediated contractile responses (*E*) were examined, five vessels per group. (*F* and *G*) Endothelium-independent vasoconstrictions (*F*) and relaxations (*G*) were evaluated in the presence of the nitric oxide synthase inhibitor L-NAME, five vessels per group. (*H*) Rmax in vasoconstrictions of the aortas were examined. (*I–L*) Endothelium-independent vasoconstrictions (*I*), Rmax in vasoconstrictions (*J*), KCl-mediated contractile responses (*K*), and endothelium-independent relaxations (*L*) were measured after surgically removal of the endothelium, six vessels per group. (*M* and *N*) The contractile responses (*M*) and Rmax (*N*) of the aortas to 10 μM–10 mM CaCl_2_ were also examined, six vessels per group. All quantification data are represented as the mean ± SEM. Statistics for concentration–response relationships were performed by calculating the area under curve followed by *t*-test analysis. *P* values were calculated by Student’s *t*-tests. *P < 0.05, **P < 0.01, ***P < 0.001.

Next, we examined the vasoreactivity of aortas derived from VSMC-YY1^cKO^ and VSMC-YY1^WT^ mice 4 weeks after Tam treatment (*Figure 1A*) using wire myography.^23^ In aortas from VSMC-YY1^cKO^ mice, phenylephrine (Phe)-mediated vasoconstrictions were significantly impaired compared to those of VSMC-YY1^WT^ mice, whereas acetylcholine (Ach)-induced relaxations remained unaffected (*Figure 1B* and *C*). Although the sensitivity/potency of Phe or Ach, as determined by EC50, showed no significant differences (see Supplementary material online, *Table S1*), aortas from VSMC-YY1^cKO^ mice exhibited a significantly reduced maximum response (Rmax) in vasoconstrictions but not in relaxations (*Figure 1D*). Moreover, KCl (60 mM)-mediated contractile responses were significantly diminished after YY1 depletion (*Figure 1E*). To evaluate endothelial-independent vasoreactivity, vessels were treated with L-NAME. Under these conditions, VSMC-YY1^cKO^ aortas displayed significantly impaired Phe-mediated vasoconstrictions, while sodium nitroprusside (SNP)-induced relaxations were unaffected (*Figure 1F* and *G*). Similarly, the EC50 showed no significant differences (see Supplementary material online, *Table S1*), but Rmax was significantly reduced in vasoconstrictions (*Figure 1H*). After mechanical removal of the endothelium, similar results were observed. Aortas from VSMC-YY1^cKO^ mice exhibited significantly reduced Phe-mediated vasoconstrictions (*Figure 1I*) and impaired Rmax (*Figure 1J*), while EC50 remained unaffected (see Supplementary material online, *Table S1*). KCl-mediated contractile responses were also significantly reduced (*Figure 1K*). Consistently, there were no changes in SNP-induced relaxations with EC50 remaining unaffected (*Figure 1L*; Supplementary material online, *Table S1*). We extended these experiments to mesenteric resistance vessels from the same mice at 4 and 10 weeks after Tam treatment. At 4 weeks (see Supplementary material online, *Figure S2A*), mesenteric vessels from VSMC-YY1^cKO^ mice exhibited significantly reduced Phe-mediated vasoconstrictions compared to VSMC-YY1^WT^ vessels, while Ach-induced relaxations remained unaffected (see Supplementary material online, *Figure S2B* and *C*). The EC50 values showed no significant differences (see Supplementary material online, *Table S2*), but Rmax was significantly reduced in vasoconstrictions, and KCl-mediated contractile responses were also diminished (see Supplementary material online, *Figure S2D* and *E*). In L-NAME-treated mesenteric vessels, Phe-mediated vasoconstrictions were significantly impaired, while SNP-induced relaxations remained unaffected (see Supplementary material online, *Figure S2F* and *G*). Again, EC50 values demonstrated no significant differences under these conditions (see Supplementary material online, *Table S2*), but Rmax was significantly reduced in vasoconstrictions (see Supplementary material online, *Figure S2H*). At 10 weeks after Tam treatment (see Supplementary material online, *Figure S2I*), similar results were observed. Mesenteric vessels from VSMC-YY1^cKO^ mice exhibited significantly reduced Phe-mediated vasoconstrictions and unaffected SNP-induced relaxations compared to VSMC-YY1^WT^ vessels (see Supplementary material online, *Figure S2J*–*P* and *Table S2*). These findings highlight impaired vasoreactivity and contractile responses in both large and resistance vessels of VSMC-YY1^cKO^ mice. To further investigate whether YY1 regulates calcium-mediated vasoconstrictions in VSMCs, we recorded CaCl_2_ (2.5 mM)-mediated contractile responses using wire myography at 4 weeks after Tam treatment. Aortas from VSMC-YY1^cKO^ mice showed significantly reduced vasoconstrictions and Rmax, with no changes in EC50 (*Figure 1M* and *N*; Supplementary material online, *Table S2*).

To gain molecular insights into how YY1 regulates VSMC contraction, we collected aortic media from VSMC-YY1^WT^ and VSMC-YY1^cKO^ mice 7 days after Tam administration for genome-wide RNA-sequencing. We first analysed the expression levels of genes encoding Ca^2+^ channels and kinases involved in membrane depolarization, which have been previously reported in VSMCs.^1^ These included calcium voltage-gated channels (see Supplementary material online, *Figure S3A*) such as CaV1.2 (encoded by *Cacna1c*), CaV1.3 (*Cacna1d*), CaV3.1 (*Cacna1 g*), CaV3.2 (*Cacna1h*), and alpha 2 delta (*Cacna2d1*); ryanodine receptor (see Supplementary material online, *Figure S3B*) such as *Ryr2*; Ca^2+^ and sodium channels (see Supplementary material online, *Figure S3C*) such as inositol 1,4,5-trisphosphate receptor (IP3R) encoded by *Itpr* types 1–3 (see Supplementary material online, *Figure S3C*); Rho kinases (see Supplementary material online, *Figure S3D*) such as *Rock1* and *Rock2*; and protein kinase C isoforms (see Supplementary material online, *Figure S3E*) such as alpha (*Prkca1*) and beta (*Prkcb*). We also examined the expression of K^+^ channels involved in VSMC contraction^2,3^ including Ca^2+^ activated channels (see Supplementary material online, *Figure S3F*) such as KCa1.1 (*Kcnma1*), KCa2.1 (*Kcnn1*), KCa2.3 (*Kcnn3*), and KCa3.1 (*Kcnn4*); voltage-gated channels (see Supplementary material online, *Figure S3G*) such as Kv1 (*Kcna5*, *Kcna6*), Kv3 (*Kcnc4*), and Kv4 (*Kcnd1*); inwardly rectifying channels (see Supplementary material online, *Figure S3H*) such as Kir2.1 (*Kcnj2*) and Kir6.1 (*Kcnj8*); and two-pore domain channels (see Supplementary material online, *Figure S3I*) such as TASK 1 (*Kcnk3*). Notably, there were no significant differences in the expression levels of any of these genes between VSMC-YY1^WT^ and VSMC-YY1^cKO^ mice. We further assessed Ca^2+^ influx in the VSMC layer of the aorta using confocal microscopy.^26^ Intracellular Ca^2+^ concentrations of VSMCs were measured after stimulation with Phe or Ca^2+^ ionophore A23187. These experiments revealed no significant differences in Ca^2+^ influx between VSMC-YY1^WT^ and VSMC-YY1^cKO^ mice (see Supplementary material online, *Figure S3J*–*M*), suggesting that YY1 does not regulate membrane depolarization in VSMCs.

We analysed the differentially expressed genes (DEGs) between VSMC-YY1^WT^ and VSMC-YY1^cKO^ (see Supplementary material online, *Figure S4A*). Gene Ontology (GO) enrichment analysis showed significant downregulation of biological pathways associated with the collagen metabolic process (e.g. *Col1a2*, *Hif1a*), collagen fibril organization (e.g. *Col1a1*, *Tgfb2*, *Col3a1*, *Col1a2*, *Col14a1*, *Tnxb*), blood vessel remodelling (e.g. *Tgfb2*, *Angpt2*, *Nrp3*, *Adra1b*), ECM organization (e.g. *Adamts2*, *Adamtsl4*, *Tgfb2*, *Col1a1*, *Col3a1*, *Mmp14*, *Col1a2*, *Col6a6, Tnxb*), and heart development in the aortic media of VSMC-YY1^cKO^ mice (see Supplementary material online, *Figure S4B* and *Table S3*). Kyoto Encyclopedia of Genes and Genomes (KEGG) pathway analysis further revealed that signalling pathways associated with Ca^2+^ signalling (e.g. *Camk2b*, *Ntrk2*, *Mylk2*, *Mrln*, *Vegfb*, *Adra1b*, *Tpcn2*), adrenergic signalling (e.g. *Camk2b*, *Adra1b*), thyroid hormone signalling (e.g. *Thrb*, *Crebbp*, *Wnt4*), MAPK signalling (e.g. *Ntrk2*, *Tgfb2*, *Hspa1a*, *Hspa1b*, *Hspa1l*, *Vegfb*, *Egfr*, *Map4k3*), and PI3K-Akt signalling (e.g. *Ghr*, *Ntrk2*, *Vegfb*, *Col1a1*, *Cdk6*, *Col1a2*), all of which are associated with VSMC contraction, proliferation, and migration, were significantly reduced in VSMC-YY1^cKO^ (see Supplementary material online, *Figure S4C* and *Table S4*). Despite these molecular changes, histological analysis using H&E staining showed no significant differences in vessel wall thickness or thickness-to-circumference ratio between VSMC-YY1^WT^ and VSMC-YY1^cKO^ mice (see Supplementary material online, *Figure S4D*). Masson’s trichrome staining also demonstrated no obvious changes in collagen fibre organization (see Supplementary material online, *Figure S4E*), while western blot analysis revealed no significant differences in COL1A1 protein levels in VSMCs between the two groups (see Supplementary material online, *Figure S4F*). Collectively, these results suggest that YY1 deficiency in VSMCs contributes to significantly impaired vascular contraction.

### 3.2 YY1 is a transcriptional activator of *Mettl3* in VSMCs

**Figure 2:**
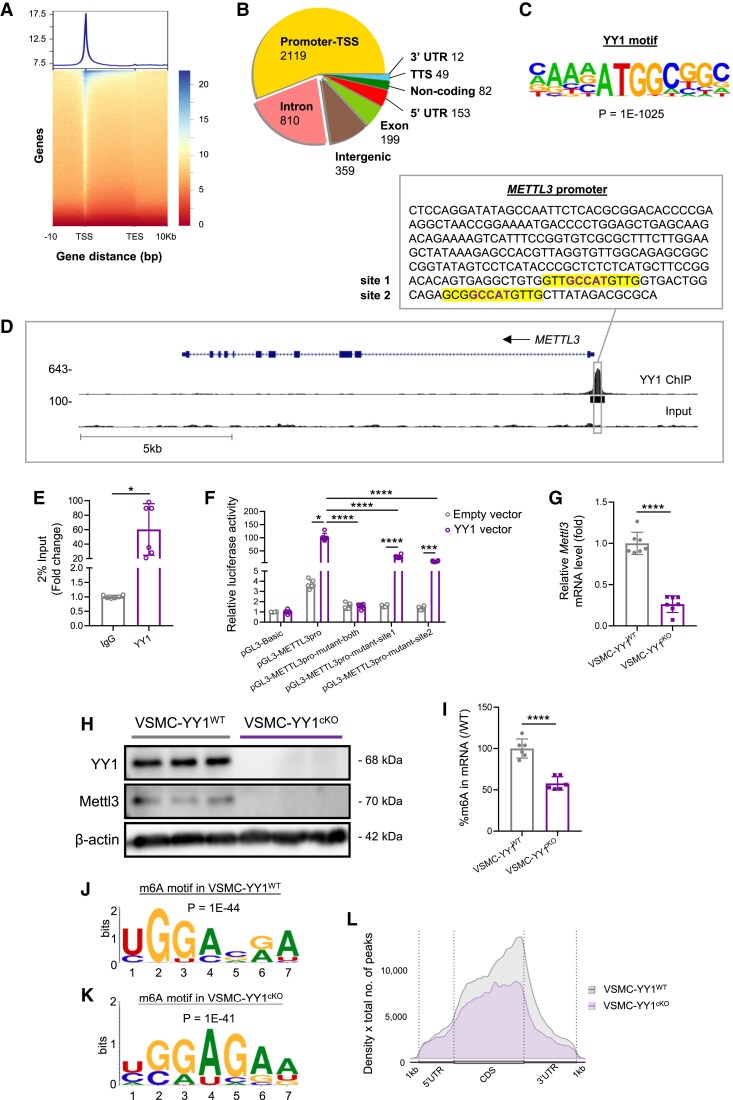
YY1 is a transcriptional activator of *Mettl3* in VSMCs. (*A*) Heatmap representing the normalized YY1 ChIP-seq intensities ± 10 kb over the gene body from transcription start site (TSS) to transcription end site (TES) of YY1-bound DNA in hESC-VSMCs. The upper panel shows an enrichment plot representing the average distribution of YY1 intensities ± 10 kb over the gene body. (*B*) Typical peak annotation pie chart shows that the majority of the peaks fall into promoter/TSS regions. (*C*) *De novo* motif discovery was identified by HOMER with YY1 ChIP-seq data. (*D*) Genome snapshot from YY1 ChIP-seq analysis showing the region and sequence of the putative *METTL3* promoter with two YY1 binding sites (highlighted). (*E*) ChIP-quantitative PCR analysis for YY1 binding to the putative *METTL3* promoter. Chromatin was extracted from hESC-VSMCs and then precipitated with an anti-YY1 antibody or IgG (negative control). The genomic DNA fragments were evaluated for enrichment by RT-qPCR using specific primers targeting the *METTL3* promoter. Data are expressed as the respective DNA inputs, six vessels per group. (*F*) Dual luciferase report assays for the *METTL3* gene promoter. The pGL3-basic vector containing the putative *METTL3* promoter region (TSS—196 bp) and promoter region with both or either one of the YY1 motifs being mutated were co-transfected with YY1 plasmid in hESC-VSMCs for 48 h, six vessels per group. (*G*) Quantitative RT-qPCR targeting *Mettl3* in aortic media of Myh11-CreER (VSMC-YY1^WT^) and Myh11-CreER;Yy1^fl/fl^ (VSMC-YY1^cKO^) mice at Day 7 after Tam treatment, seven vessels per group. (*H*) Western blot analysis using aortic media of VSMC-YY1^cKO^ and VSMC-YY1^WT^ was carried out at 2 weeks after Tam, representatives from six vessels per group. (*I*) Quantification showing the relative m^6^A levels among mRNAs in aortic media of VSMC-YY1^cKO^ and VSMC-YY1^WT^ at 7 days after Tam, six vessels per group. (*J* and *K*) The respective m^6^A motifs of meRIP-seq (m^6^A-seq) in aortic media of VSMC-YY1^cKO^ and VSMC-YY1^WT^ at 7 days after Tam. (*L*) Metaplot of m^6^A-seq showing the m^6^A distribution enriched at the site nearby stop codon in aortic media. All quantification data are represented as the mean ± SEM. The *P* values were calculated by Student’s *t*-tests or two-way ANOVA followed by Tukey’s multiple comparisons. *P < 0.05, ****P < 0.0001.

To identify gene targets directly regulated by YY1 in VSMCs, we performed chromatin immunoprecipitation followed by deep sequencing (ChIP-seq) in contractile VSMCs. Human primary aortic smooth muscle cells (HASMCs) were induced to adopt a contractile phenotype using TGFβ (10 ng/mL) treatment for 24 h, as previously described.^27^ Western blot analysis confirmed significantly increased expression of downstream targets SM22α^28^ and αSMA^29^ in HASMCs after TGFβ treatment compared to solvent-treated controls (see Supplementary material online, *Figure S5A*). However, MYH11 and MLCK were minimally expressed in HASMCs even after TGFβ induction (see Supplementary material online, *Figure S5A*). As a result, we differentiated contractile VSMCs from human embryonic stem cells (hESC-VSMCs) using a previously reported protocol.^30^ Unlike HASMCs, which proliferate readily in cultures, contractile hESC-VSMCs showed minimal proliferation and expressed high levels of MYH11, MLCK, SM22α, and αSMA (see Supplementary material online, *Figure S5B* and *C*), highly reminiscent of primary VSMCs. Besides, the expression levels of these contractile proteins were significantly higher in hESC-VSMCs compared to TGFβ-treated HASMCs (see Supplementary material online, *Figure S5C*). Therefore, we performed ChIP-seq experiments using contractile hESC-VSMCs instead of HASMCs (*Figure 2A*). ChIP-seq analysis revealed that YY1 predominantly bound to the promoter or transcription start site regions of the genome (*Figure 2B*). Consistent with the motif reported in pancreatic beta cells,^12^ our *de novo* motif analysis identified CGGTA/GCCAT as the core motif sequence in hESC-VSMCs, which was preferentially bound by YY1 in 53.71% of peaks identified on its gene targets (*P* = 1 × 10^−1025^) (*Figure 2C*). A total of 3783 peaks were identified, 3319 of which were from protein-coding transcripts. Subsequent analyses focused on the 3083 protein-coding genes with YY1 binding peaks.

Considering the impaired vasoconstriction observed in VSMC-YY1^cKO^ mice despite membrane depolarization or exogenous Ca^2+^ supplementation (*Figure 1L*), we first examined whether YY1 directly regulates transcription of genes in the Ca^2+^ signalling pathway that were significantly reduced in the aortic media of VSMC-YY1^cKO^ mice compared to VSMC-YY1^WT^ (see Supplementary material online, *Figure S4C* and *Table S4*). However, ChIP-seq analysis showed no YY1 binding peaks at these down-regulated Ca^2+^ signalling genes (see Supplementary material online, *Figure S6*). We then conducted integrative analyses using ChIP-seq data from hESC-VSMCs and RNA-seq data from aortic media of VSMC-YY1^WT^ and VSMC-YY1^cKO^ mice at 7 days after Tam administration. This approach identified 293 differentially expressed protein-coding genes with YY1 binding peaks, 174 of which displayed the canonical core motif sequence. Among these genes, *Mettl3* stood out as one of the most significantly altered genes in aortic media of VSMC-YY1^cKO^, with two YY1 binding sites in its putative promoter region (*Figure 2D*). Bioinformatics analysis showed that these YY1 binding sites at the *Mettl3* promoter were conserved across mouse and human genomes (see Supplementary material online, *Figure S7*). ChIP-qPCR further validated significantly increased YY1 enrichment at the *METTL3* promoter in hESC-VSMCs (*Figure 2E*). To confirm the function of YY1 in regulating *METTL3* transcription, we performed firefly luciferase reporter assays. Luciferase signals driven by the GCCAT-containing putative *METTL3* promoter were significantly higher compared to signals driven by putative promoter after mutating both (pGL3-METTL3pro-mutant-both) or either one (pGL3-METTL3pro-mutant-site1/site2) of the core motif sequences in lentivirus-delivered YY1 overexpressing hESC-VSMCs (*Figure 2F*). This demonstrated that YY1 binding to the *METTL3* promoter is required for directly activating its transcription. Consistent with these findings, RT-qPCR revealed significantly reduced mRNA levels of *Mettl3* in aortic media of VSMC-YY1^cKO^ compared to VSMC-YY1^WT^ mice at 7 days after Tam administration (*Figure 2G*). Western blot analysis further confirmed that METTL3 protein levels were nearly undetectable in VSMC-YY1^cKO^ mice at 14 days after Tam treatment (*Figure 2H*). As METTL3 is an m^6^A RNA methyltransferase (writer), we assessed its functional impact by measuring m^6^A levels in mRNA from the aortic media. The percentage of m^6^A in mRNA was significantly reduced in VSMC-YY1^cKO^ compared to VSMC-YY1^WT^ mice at 7 days after Tam administration (*Figure 2I*). Further m^6^A-seq analysis identified m^6^A consensus motifs GGACA^31^ and GGAGA^32^ in mRNA purified from the aortic media of VSMC-YY1^WT^ and VSMC-YY1^cKO^ mice at 7 days after Tam administration, respectively (*Figure 2J* and *K*). m^6^A-seq also demonstrated a marked reduction in m^6^A enrichment at coding sequences (CDSs), stop codons, and 3′UTRs in mRNA of VSMC-YY1^cKO^ compared to VSMC-YY1^WT^ (*Figure 2L*). These findings collectively demonstrate that Mettl3 is a direct gene target of YY1 in VSMCs. Loss of YY1 in VSMCs leads to reduced METTL3-mediated m^6^A mRNA methylation, potentially contributing to the observed vascular dysfunction in VSMC-YY1^cKO^ mice.

### 3.3 VSMC Mettl3 controls vascular contraction *in vivo*

**Figure 3:**
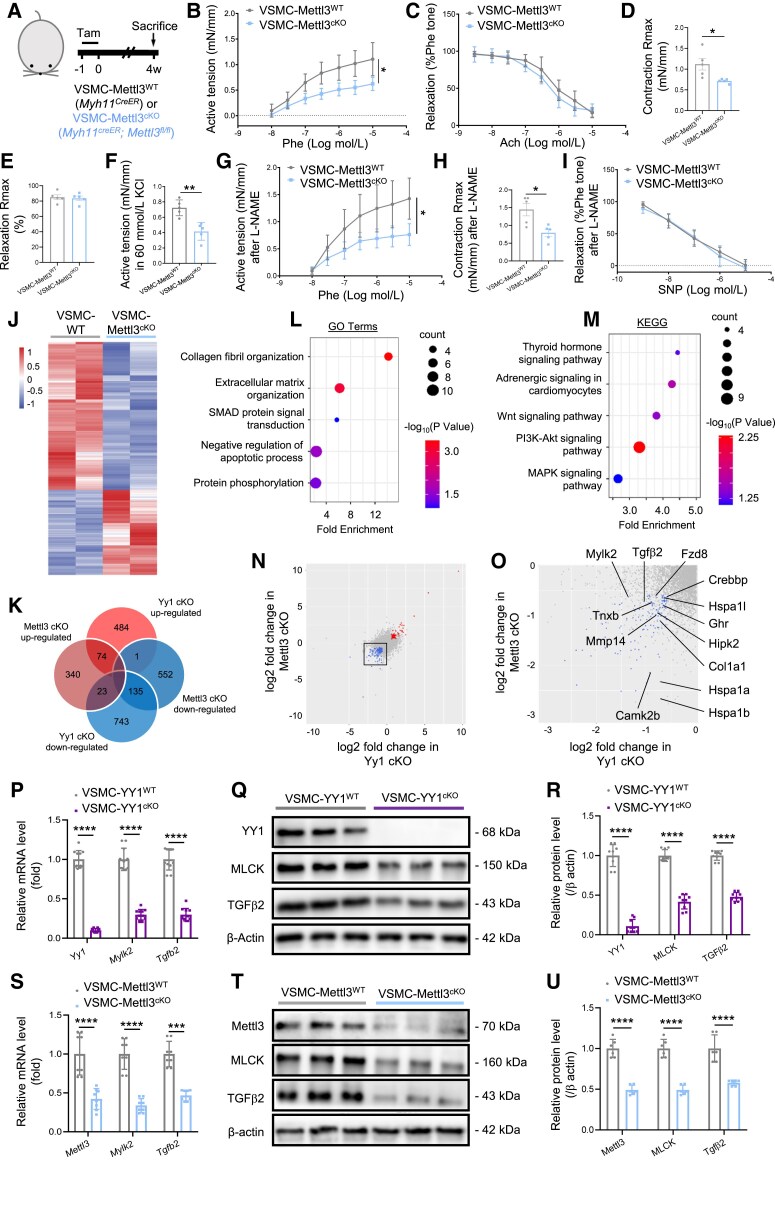
Loss of Mettl3 in VSMCs impairs vascular contraction *in vivo*. (*A*) A schematic showing the experimental design and the generation of Myh11-CreER;Mettl3^fl/fl^ (VSMC-Mettl3^cKO^) and Myh11-CreER (VSMC-Mettl3^WT^) mice. (*B*) Concentration-dependent vasoconstriction responses of mouse aortas to Phe and (*C*) concentration-dependent relaxation responses to Ach were measured in VSMC-Mettl3^cKO^ and VSMC-Mettl3^WT^ aortas 28 after Tam treatment, five vessels per group. (*D–F*) The maximum responses (Rmax) in vasoconstrictions (*D*) and relaxations (*E*) or after 60 mM KCl stimulation (*F*) were examined, five vessels per group. (*G–I*) Endothelium-independent vasoconstrictions (*G*), Rmax in vasoconstrictions (*H*), and endothelium-independent relaxations (*I*) were evaluated in the presence of the nitric oxide synthase inhibitor L-NAME, five vessels per group. (*J*) Heatmap of top 800 DEGs derived from bulk RNA-seq in aortic media of VSMC-Mettl3^cKO^ compared to VSMC-Mettl3^WT^ at Day 7 after Tam. (*K*) Venn diagram summarizing the numbers of commonly up-regulated and down-regulated genes in VSMC-Mettl3^cKO^ and VSMC-YY1^cKO^ mice compared to their respective controls of same genotype. GO enrichment (*L*) and KEGG pathway analysis (*M*) of significantly down-regulated genes in transcription. (*N*) Comparison of all genes identified in RNA-seq experiments. Red dots denote significantly and commonly up-regulated genes; and blue dots denote significantly and commonly down-regulated genes. (*O*) Down-regulated genes from pathways in (*L*) and (*M*) that showed differential m^6^A methylation in aortic VSMCs of VSMC-YY1^cKO^ compared to VSMC-YY1^WT^ as identified by integrative analysis of bulk RNA-seq and m^6^A-seq. (*P*) Quantitative RT-qPCR using aortic media of VSMC-YY1^cKO^ and VSMC-YY1^WT^ at Day 7 after Tam, 11 vessels per group. (*Q*) Western blot analysis and (*R*) quantification using aortic media of VSMC-YY1^cKO^ and VSMC-YY1^WT^ at 2 weeks after Tam, nine vessels per group. (*S*) Quantitative RT-qPCR using aortic media of VSMC-Mettl3^cKO^ and VSMC-Mettl3^WT^ at Day 7 after Tam, eight vessels per group. (*T*) Western blot analysis and (*U*) quantification using aortic media of VSMC-Mettl3^cKO^ and VSMC-Mettl3^WT^ at day 14 after Tam, six vessels per group. All quantification data are represented as the mean ± SEM. Statistics for concentration–response relationships were performed with calculation of area under curve followed by *t*-test analysis. The *P* values were calculated by Student’s *t*-tests or two-way ANOVA followed by Tukey’s multiple comparisons. *P < 0.05, **P < 0.01, ***P < 0.001, ****P < 0.0001.

To study the biological function of Mettl3 in VSMCs, we generated VSMC-specific *Mettl3* knockout mice, *Myh11^CreER^;Mettl3^fl/fl^* (hereafter referred to as VSMC-Mettl3^cKO^), by crossing *Myh11^CreER^* with *Mettl3^fl/fl^* (*Figure 3A*). Control mice, *Myh11^CreER^* (hereafter named as VSMC-Mettl3^WT^), were of the same genotype as VSMC-YY1^WT^. We first measured VSMC vasoreactivity by performing wire myography on aortas collected 4 weeks after Tam treatment. Similar to the phenotype observed in VSMC-YY1^cKO^ mice, Phe-mediated vasoconstrictions were significantly impaired in aortas of VSMC-Mettl3^cKO^ mice compared to controls, while Ach-induced relaxations remained unaffected (*Figure 3B* and *C*). Although EC50 showed no significant differences (see Supplementary material online, *Table S5*), the aortas of VSMC-Mettl3^cKO^ mice exhibited a significantly reduced Rmax in vasoconstrictions but not in relaxations (*Figure 3D* and *E*). Additionally, KCl-mediated contractile responses were significantly diminished (*Figure 3F*). To evaluate endothelial-independent vasoreactivity, vessels were treated with L-NAME. Under these conditions, VSMC-Mettl3^cKO^ aortas displayed significantly impaired Phe-mediated vasoconstrictions and reduced Rmax (*Figure 3G, H*), while SNP-induced relaxations were unaffected (*Figure 3I*). We extended these experiments to mesenteric resistance vessels collected from the same mice at both 4 and 10 weeks after Tam treatment (see Supplementary material online, *Figure S8* and *Table S6*). At both time points, Phe-mediated vasoconstrictions were significantly reduced in mesenteric vessels from VSMC-Mettl3^cKO^ mice compared to VSMC-Mettl3^WT^ controls, while Ach-induced relaxations remained unaffected. Similarly, while EC50 values showed no significant differences, Rmax was significantly reduced in vasoconstrictions. KCl-mediated contractile responses were also significantly reduced in mesenteric vessels from VSMC-Mettl3^cKO^ mice. In L-NAME-treated mesenteric vessels, Phe-mediated vasoconstrictions and Rmax were significantly reduced, while SNP-induced relaxations remained unchanged. These findings demonstrate that Mettl3 is likely a direct target of YY1 in VSMCs, as evidenced by the similar impairments in contractile function observed after lineage-specific genetic ablation of either YY1 or Mettl3 *in vivo*.

To further understand the regulatory network by which the YY1/Mettl3 axis maintains VSMC contraction, we performed RNA-seq analysis on the aortic media of VSMC-Mettl3^cKO^ and control VSMC-Mettl3^WT^ mice 7 days after Tam administration. DEGs were identified (*Figure 3J*), and the overlapping DEGs between VSMC-YY1^cKO^ and VSMC-Mettl3^cKO^ were analysed. Among all DECs, 74 genes were significantly and commonly up-regulated, whereas 135 genes were significantly and commonly down-regulated in both VSMC-YY1^cKO^ and VSMC-Mettl3^cKO^ mice compared to their controls (*Figure 3K*). Gene-set enrichment analysis of the up-regulated DEGs identified only three significantly enriched pathways including Fanconi anaemia, selenocompound metabolism, and one carbon pool by folate. Conversely, GO enrichment analysis of the down-regulated DEGs revealed several key biological pathways that were significantly and commonly affected in aortic media of both VSMC-YY1^cKO^ and VSMC-Mettl3^cKO^ mice, including collagen fibril organization (e.g. *Col1a1*, *Tgfb2*, *Tnxb*), ECM organization (e.g. *Col1a1*, *Tgfb2*, *Adamts2*, *Mmp14*, *Adamtsl4*, *Tnxb*), SMAD protein signal transduction (e.g. *Tgfb2*, *Gdf15*, *Hipk2*), negative regulation of apoptosis (e.g. *Tgfb2*, *Vegfb*), and protein phosphorylation (e.g. *Camk2b*, *Mylk2*, *Ntrk2*, *Tgfb2*, *Hipk2*) (*Figure 3L*; Supplementary material online, *Table S7*). Moreover, KEGG pathway analysis revealed significant and common downregulation of pathways associated with thyroid hormone signalling (e.g. *Crebbp*, *Wnt4*), adrenergic signalling (e.g. *Camk2b*), Wnt signalling (e.g. *Camk2b*, *Tle3*, *Crebbp*, *Fzd8*, *Wnt4*), PI3K-Akt signalling (e.g. *Ghr*, *Ntrk2*, *Col1a1*, *Cdk6*, *Col1a2*), and MAPK signalling (e.g. *Ntrk2*, *Tgfb2*, *Hspa1a*, *Hspa1b*, *Hspa1l*) (*Figure 3M*; Supplementary material online, *Table S8*). Given that Mettl3 is an m^6^A RNA methyltransferase, we further examined the mRNA transcripts of these significantly and commonly down-regulated genes in reference to their m^6^A methylation status as determined by m^6^A-seq. Integrative analysis demonstrated that several genes, including *Camk2b*, *Col1a1*, *Crebbp*, *Fzd8*, *Ghr*, *Hipk2*, *Hspa1a*, *Hspa1b*, *Hspa1l*, *Mmp14*, *Mylk2*, *Tgfb2*, and *Tnxb*, were among the most significantly down-regulated in RNA-seq and showed significantly different m^6^A methylation on their mRNAs in VSMC-YY1^cKO^ compared to VSMC-YY1^WT^ (*Figure 3N* and *O*).

Considering their function in the maintenance of contractile phenotype of VSMCs, we focused to validate if *Mylk2* and *Tgfb2* are downstream targets of the YY1/Mettl3 regulatory axis. We performed knockdown and overexpression experiments in hESC-VSMCs and conducted *in vivo* validation in mice. First, *YY1* expression was knocked down in hESC-VSMCs using siRNA (20 nM) for 48 h, which resulted in significantly reduced MLCK and TGFβ2 levels compared to control siRNA (see Supplementary material online, *Figure S9A* and *B*). Conversely, overexpression of YY1 using chemically synthesized 5-methylcytidine- and pseudouridine-modified mRNA (modRNA), as previously described^33^ to enhance transcript stability and translation efficiency, significantly increased MLCK and TGFβ2 expression levels 48 h after transfection compared to eGFP modRNA controls (see Supplementary material online, *Figure S9A* and *B*). Nonetheless, ChIP-seq analysis showed no direct YY1 binding to the DNA of *MYLK2* (see Supplementary material online, *Figure S6J*) or *TGFB2* (see Supplementary material online, *Figure S9C*), indicating that they are not direct gene targets of YY1 in VSMCs. Similarly, METTL3 knockdown with siRNA significantly reduced MLCK and TGFβ2 levels in hESC-VSMCs, while METTL3 overexpression using modRNA significantly increased their expression (see Supplementary material online, *Figure S10A* and *B*). To validate these findings *in vivo*, we analysed aortic media from VSMC-YY1^cKO^ and VSMC-Mettl3^cKO^ mice. RT-qPCR at Day 7 after Tam treatment and western blot at day 14 demonstrated that *Mylk2*/MLCK and TGFβ2 mRNA and protein levels were significantly down-regulated in aortic media of VSMC-YY1^cKO^ compared to VSMC-YY1^WT^ mice (*Figure 3P–R*). Similarly, *Mylk2*/MLCK and TGFβ2 levels were significantly reduced in aortic media of VSMC-Mettl3^cKO^ compared to VSMC-Mettl3^WT^ mice (*Figure 3S–U*). Collectively, these findings suggest that while *Mylk2*/MLCK and TGFβ2 are not direct transcriptional targets of YY1, their expression is regulated by the YY1/Mettl3 axis. The loss of YY1 or Mettl3 leads to decreased levels of MLCK and TGFβ2, contributing to impaired vascular contraction. This strongly supports the critical role of the YY1/Mettl3 regulatory axis in maintaining the contractile phenotype of VSMCs and vascular function.

### 3.4 YY1/Mettl3 regulates mRNA stability of *Mylk2* and *Tgfb2* in VSMCs

**Figure 4:**
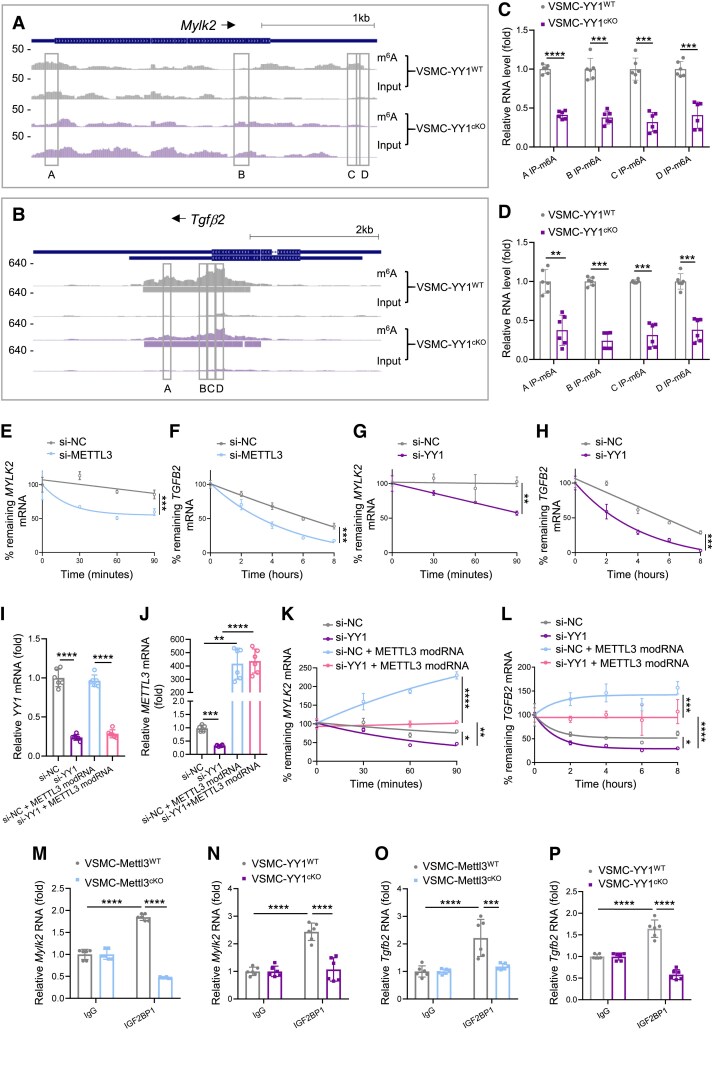
YY1/Mettl3 regulates mRNA stability of *Mylk2* and *Tgfb2* in VSMCs. (*A* and *B*) The m^6^A methylation landscape of *Mylk2* and *Tgfb2* mRNA in aortic media of VSMC-YY1^cKO^ and VSMC-YY1^WT^ mice at Day 7 after Tam as determined by m^6^A RNA immunoprecipitation (RIP) followed by high throughput sequencing (m^6^A-seq). Significant peaks are indicated in horizontal rectangles; and regions of differential methylation are highlighted with boxes. (*C* and *D*) m^6^A RIP followed by quantitative RT-qPCR, six vessels per group. RNA was extracted from aortic media of VSMC-YY1^cKO^ and VSMC-YY1^WT^ mice at Day 7 after Tam and then precipitated with an anti-m^6^A antibody. The RNA fragments were evaluated for enrichment by RT-qPCR using specific primers targeting the m^6^A peak regions of *Mylk2* and *Tgfb2* mRNA as indicated in boxes of (*A*) and (*B*) respectively. (*E–H*) Decay curves of *MYLK2* and *TGFB2* mRNAs were quantified in hESC-VSMCs after being treated with *METTL3* (*E* and *F*) or *YY1* (*G* and *H*) siRNA (20 nM) for 24 h, six samples per group. Transcription was terminated by treatment of cells with actinomycin D (5 μg/mL), and total RNA was extracted at the indicated time point. The mRNA levels were measured by RT-qPCR using specific primers targeting *MYLK2* and *TGFB2* mRNA. (*I* and *J*) RT-qPCR targeting *YY1* (*I*) and *METTL3* (*J*) using hESC-VSMCs in different treatments, six vessels per group. (*K* and *L*) Decay curves of *MYLK2* (*K*) and *TGFB2* (*L*) mRNAs were quantified in hESC-VSMCs after being treated with *YY1* siRNA and *METTL3* modified mRNA (modRNA, 1 μg/mL) for 48 h, six vessels per group. (*M–P*) IGF2BP1 RIP followed by RT-qPCR, six vessels per group. RNA was extracted from aortic media of VSMC-Mettl3^cKO^ and VSMC-Mettl3^WT^ mice or VSMC-YY1^cKO^ and VSMC-YY1^WT^ mice at Day 7 after Tam and then precipitated with an anti-IGF2BP1 antibody. The RNA fragments were evaluated for enrichment by RT-qPCR using specific primers targeting the *Mylk2* (*M* and *N*) or *Tgfb2* (*O* and *P*) mRNA. All quantification data are represented as the mean ± SEM. The *P* values were calculated by two-way ANOVA followed by Tukey’s multiple comparisons. Statistical analysis of time–response relationships was performed by calculating the area under curve, followed by Student’s *t*-tests, or by two-way ANOVA with Tukey’s multiple comparisons. **P < 0.01, ***P < 0.001, ****P < 0.0001.

Mettl3-mediated m^6^A RNA methylation regulates gene expression via multiple mechanisms, as m^6^A modifications can directly or indirectly influence the binding of reader proteins to methylated mRNA, thereby affecting RNA metabolism, including degradation, stabilization, and translation initiation (for review, see Frye^34^) For instance, recognition of specific m^6^A-methylated transcripts by the IGF2BP family proteins enhances mRNA stability and translation.^35^ Based on this, we hypothesized that YY1 regulates the expression of MLCK and TGFβ2 through Mettl3-mediated m^6^A RNA methylation. m^6^A-seq analysis revealed reduced methylation at the 5′UTR, CDS, and 3′UTR regions of *Mylk2* (*Figure 4A*), as well as decreased methylation at the CDS, stop codon, and 3′UTR regions of *Tgfb2* (*Figure 4B*) in the aortic media of VSMC-YY1^cKO^ compared to VSMC-YY1^WT^ mice at 7 days after Tam administration. These findings were validated with RIP by m^6^A followed by RT-qPCR, targeting the methylated regions, which confirmed significantly reduced m^6^A levels at these regions of both *Mylk2* (*Figure 4C*) and *Tgfb2* (*Figure 4D*) in VSMC-YY1^cKO^ mice. Although neither gene is a direct target of YY1, these results confirmed that *Mylk2* and *Tgfb2* are directly subjected to m^6^A mRNA methylation.

Given the significant reduction in both mRNA and protein levels of *Mylk2* and *Tgfb2* in VSMC-YY1^cKO^ and VSMC-Mettl3^cKO^ mice, we hypothesized that YY1 regulates gene expression via Mettl3-mediated m^6^A methylation by controlling mRNA stability and translation efficiency. To prove this, we measured mRNA decay rates using actinomycin D (5 µg/mL) in hESC-VSMCs. After 24 h of *METTL3* siRNA treatment, *MYLK2* and *TGFβ2* mRNA decayed more rapidly (*Figure 4E* and *F*), indicating that METTL3 regulates transcript stability. Similarly, *YY1* siRNA treatment also accelerated the decay of *MYLK2* and *TGFβ2* mRNA (*Figure 4G* and *H*). To further confirm this mechanism, we re-expressed METTL3 in hESC-VSMCs treated with *YY1* siRNA by introducing *METTL3* modRNA. RT-qPCR confirmed successful knockdown of *YY1* by siRNA (*Figure 4I*) and significant upregulation of *MELTT3* after 48 h of modRNA treatment, even in the presence of *YY1* siRNA (*Figure 4J*). Importantly, the transcript stability of *MYLK2* (*Figure 4K*) and *TGFβ2* (*Figure 4L*) was partially rescued in the *METTL3* modRNA and *YY1* siRNA co-treatment group compared to the *YY1* siRNA-only treatment group, confirming that YY1 regulates transcript stability via Mettl3. Finally, we quantified recognition of *MYLK2* and *TGFβ2* transcripts by IGF2BP1, a known m^6^A reader protein, in VSMCs using RIP followed by RT-qPCR. Significantly reduced amounts of *MYLK2* RNA were immunoprecipitated by IGF2BP1 in the aortic media of VSMC-Mettl3^cKO^ (*Figure 4M*) and VSMC-YY1^cKO^ (*Figure 4N*) compared to their respective controls at 7 days after Tam administration. Similarly, significantly decreased amounts of *TGFβ2* RNA were immunoprecipitated by IGF2BP1 in VSMC-Mettl3^cKO^ (*Figure 4O*) and VSMC-YY1^cKO^ (*Figure 4P*) compared to controls. Altogether, these findings indicate that the YY1/Mettl3 axis epitranscriptomically regulates the mRNA stability of *MYLK2* and *TGFβ2*, potentially through the recognition of m^6^A-methylated mRNA by IGF2BP1 in VSMCs.

### 3.5 YY1 controls vascular contraction through Mettl3 activation *in vivo*

**Figure 5:**
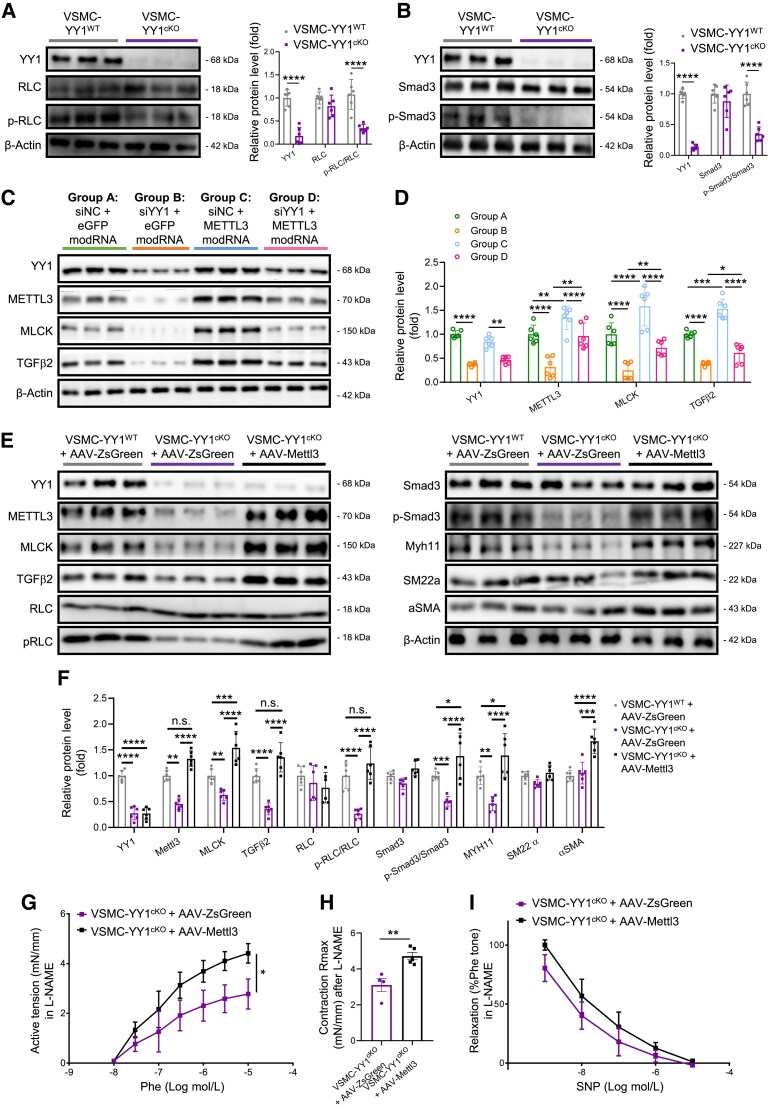
YY1 controls vascular contraction through Mettl3 activation *in vivo*. (*A* and *B*) Western blot analyses and quantification targeting expression and phosphorylation (p-) levels of RLC (*A*) and Smad3 (*B*) in aortic media of VSMC-YY1^cKO^ and VSMC-YY1^WT^ mice at Day 14 after Tam, respectively, six vessels per group. (*C*) Western blot analysis and (*D*) quantification in hESC-VSMCs after being treated with siRNA (20 nM) for 72 h and with modified mRNA (modRNA, 1 μg/mL) for 48 h, six samples per group. (*E–H*) VSMC-YY1^WT^ mice were intravenously injected with AAV-SM22-ZsGreen (AAV-ZsGreen) and VSMC-YY1^cKO^ mice were injected with AAV-ZsGreen or AAV-SM22-Mettl3 (AAV-Mettl3). Mice were first subjected to AAV-mediated gene delivery for 4 weeks, followed by Tam for another 4 weeks before tissue collection, six vessels per group. Aortic media were purified for western blot analyses (*E*) and quantification (*F*) of protein expression levels. Aortas were harvested for examination on concentration-dependent vasoconstrictions (*G*), Rmax in vasoconstrictions (*H*), and relaxations (*I*) in response to Phe and SNP, respectively. Endothelium-mediated vasoconstrictions and relaxations were antagonized by co-treatment with the nitric oxide synthease inhibitor L-NAME. All quantification data are represented as the mean ± SEM. The *P* values were calculated by *t*-tests or two-way ANOVA followed by Tukey’s multiple comparisons. Statistics for concentration–response relationships were performed with calculation of area under curve followed by *t*-test analysis (*G* and *I*). *P < 0.05, **P < 0.01, ***P < 0.001, ****P < 0.0001.

To confirm whether the YY1/Mettl3 regulatory axis activates MLCK and TGFβ signalling in VSMCs, we examined the phosphorylation (p-) levels of their downstream targets, RLC and Smad3, after YY1 knockout. At 2 weeks after Tam treatment, phosphorylation levels but not total expression levels of RLC (*Figure 5A*) and Smad3 (*Figure 5B*) were significantly reduced in the aortic media of VSMC-YY1^cKO^ compared to VSMC-YY1^WT^ mice. Next, we examined whether overexpression of METTL3 could rescue the expression of MLCK and TGFβ2 in hESC-VSMCs after YY1 knockdown. *METTL3* modRNA was administered 24 h after *YY1* siRNA induction, and cells were harvested for western blot analysis 48 h after modRNA treatment (*Figure 5C*). We showed that *YY1* siRNA significantly reduced the expression of YY1, METTL3, MLCK, and TGFβ2 compared to control siRNA-treated cells (*Figure 5C* and *D*; Group B vs. Group A). *METTL3* modRNA significantly up-regulated the expression of MLCK and TGFβ2 compared to eGFP modRNA-treated cells (*Figure 5C* and *D*; Group C vs. Group A). Importantly, *METTL3* modRNA partially salvaged the expression of MLCK and TGFβ2 in hESC-VSMCs after *YY1* siRNA treatment (*Figure 5C* and *D*; Group D vs. Group B), likely due to the restoration of *MYLK2* and *TGFB2* transcript stability (*Figure 4K* and *L*).

To further validate these findings *in vivo*, we assessed whether Mettl3 overexpression in VSMCs could improve vascular function in VSMC-YY1^cKO^ mice. VSMC-YY1^WT^ (positive control) and VSMC-YY1^cKO^ (negative control) mice were injected intravenously with AAV-mediated SM22-specific ZsGreen (AAV-ZsGreen control), while VSMC-YY1^cKO^ mice were also injected with AAV-mediated SM22-specific Mettl3 (AAV-Mettl3). At 4 weeks after AAV-mediated gene delivery, mice were administered Tam, and aortic media were harvested for western blot or aortas were collected for wire myography 4 weeks after Tam treatment. Similar to the *in vitro* experiments, expression levels of MLCK and TGFβ2 were significantly increased in AAV-Mettl3-treated compared to AAV-ZsGreen-treated VSMC-YY1^cKO^ mice (*Figure 5E* and *F*). As a result, p-RLC/RLC and p-Smad3/Smad3 levels were also significantly increased in AAV-Mettl3-treated compared to AAV-ZsGreen-treated VSMC-YY1^cKO^ mice. Additionally, downstream targets^28,29,36^ of TGFβ/Smad3 signalling, including *Myh11*, *Tagln* (encoding SM22α), and *Acta2* (encoding αSMA), were significantly increased after Mettl3 overexpression. Specifically, MYH11 and αSMA were significantly increased in AAV-Mettl3-treated than AAV-ZsGreen-treated VSMC-YY1^cKO^ mice. We further investigated whether MYH11 and αSMA expression are regulated by m^6^A modification. m^6^A-seq analysis revealed m^6^A peaks at the 3′ end of the *Myh11* gene but not the *Acta2* gene (see Supplementary material online, *Figure S11A* and *B*), suggesting that MYH11 expression could be regulated by m^6^A methylation. This was validated using RIP followed by RT-qPCR, which confirmed significantly reduced m^6^A levels at the methylated regions of *Myh11* in the aortic media of VSMC-YY1^cKO^ mice (see Supplementary material online, *Figure S11C*). To determine whether Myh11 is a target gene of Mettl3, RT-qPCR analysis demonstrated that MYH11 mRNA levels were significantly reduced after *METTL3* siRNA treatment but significantly increased after *METTL3* modRNA treatment in hESC-VSMCs (see Supplementary material online, *Figure S11D*). Additionally, mRNA decay assays revealed that *MYH11* mRNA decayed more rapidly following 24 h of either *YY1* or *METTL3* siRNA treatment (see Supplementary material online, *Figure S11E* and *F*), indicating that METTL3 regulates the transcript stability of *MYH11*. The transcript stability of *MYH11* was rescued in the *METTL3* modRNA and *YY1* siRNA co-treatment group compared to the *YY1* siRNA-only treatment group (see Supplementary material online, *Figure S11G*), confirming that YY1 regulates *MYH11* transcript stability via Mettl3. Furthermore, RIP analysis showed significantly reduced amounts of *MYH11* RNA immunoprecipitated by IGF2BP1 in the aortic media of VSMC-YY1^cKO^ (see Supplementary material online, *Figure S11H*) and VSMC-Mettl3^cKO^ (see Supplementary material online, *Figure S11I*) mice compared to their respective controls at 7 days after Tam administration. Functionally, we observed significantly improved Phe-mediated vasoconstrictions and increased Rmax in aortas derived from the AAV-Mettl3 group compared to the AAV-ZsGreen group (*Figure 5G* and *H*). However, there was no significant difference in SNP-induced relaxations between the two groups (*Figure 5I*). Additionally, the EC50 values for responses to Phe or Ach showed no significant differences (see Supplementary material online, *Table S9*). Collectively, these findings confirm that Mettl3 is a direct downstream target of YY1 in VSMCs. YY1 regulates vasoreactivity by stabilizing *Mylk*, *Tgfβ2*, and *Myh11* mRNA in VSMCs through directly promoting *Mettl3* transcription and m^6^A methylation.

### 3.6 YY1 activates *Mettl3* transcription by recruiting the WSC complex to promoter

**Figure 6:**
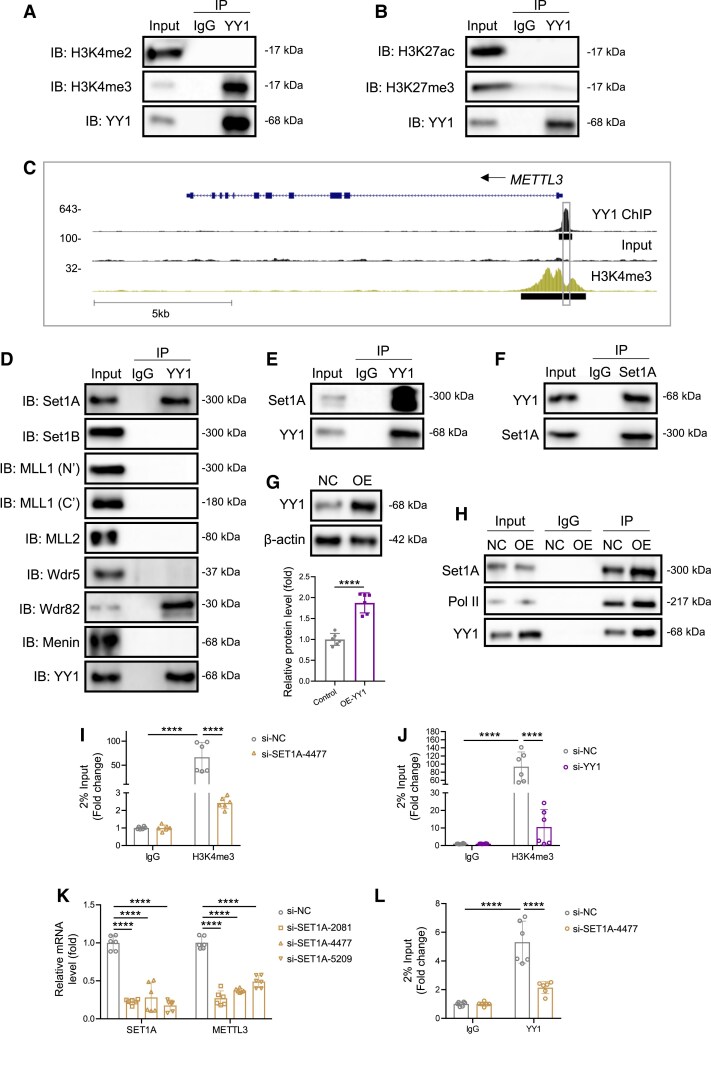
YY1 activates *Mettl3* transcription by recruiting WSC complex to the promoter. (*A*) Co-IP showing the endogenous protein interaction between YY1 and histone 3 lysine 4 di (H3K4me2)- or tri (H3K4me3)-methylation in murine aortic media. Protein was extracted and then precipitated with an anti-YY1 antibody or IgG (negative control) before immunoblot. (*B*) Co-IP showing the endogenous protein interaction between YY1 and histone 3 lysine 27 acetylation (H3K27ac) or tri-methylation (H3K27me3) in murine aortic media. Protein was extracted and then precipitated with an anti-YY1 antibody or IgG before immunoblot. (*C*) Genome snapshots from YY1 and H3K4me3 ChIP-seq analyses performed in human VSMCs at the Mettl3 locus. The putative promoter region was also highlighted. (*D*) Co-IP showing the endogenous protein interaction between YY1 and H3K4me3 writers or co-factors in murine aortic media. Protein was extracted and then precipitated with an anti-YY1 antibody or IgG before immunoblot. (*E* and *F*) Reciprocal co-IP showing the endogenous protein interaction between YY1 and SET1A in hESC-VSMCs. Protein was extracted and then precipitated with an anti-YY1 antibody (*E*), anti-SET1A antibody (*F*), or IgG. (*G*) Western blot and quantification at 48 h after lentivirus-mediated YY1 delivery in hESC-VSMCs, six vessels per group. (*H*) Co-IP showing the endogenous protein interaction between YY1 and SET1A or RNA polymerase II in hESC-VSMCs. Protein was extracted and then precipitated with an anti-YY1 antibody or IgG. (*I* and *J*) ChIP-quantitative PCR analysis for H3K4me3 binding to the putative *METTL3* promoter, six samples per group. Chromatin was extracted from hESC-VSMCs treated with control (siNC), *SET1A* siRNA (20 nM, *I*), or *YY1* siRNA (20 nM, *J*) for 48 h and then precipitated with an anti-H3K4me3 antibody or IgG. The genomic DNA fragments were evaluated for enrichment by RT-qPCR using specific primers targeting the *METTL3* promoter. (*K*) Quantitative RT-qPCR targeting *SET1A* and *METTL3* in hESC-VSMCs treated with control or *SET1A* siRNA for 48 h, six samples per group. (*L*) ChIP-quantitative PCR analysis for YY1 binding to the putative *METTL3* promoter, six samples per group. Chromatin was extracted from hESC-VSMCs treated with control (siNC) or *SET1A* siRNA for 48 h and then precipitated with an anti-YY1 antibody or IgG. The genomic DNA fragments were evaluated for enrichment by RT-qPCR using specific primers targeting the *METTL3* promoter. All quantification data are represented as the mean ± SEM. The *P* values are calculated by Student’s *t*-tests or two-way ANOVA followed by Tukey’s multiple comparisons. ****P < 0.0001.

The above findings demonstrate that YY1 directly activated *METTL3* transcription in VSMCs by modifying its promoter. To obtain further insights into the mechanism of YY1-mediated transcriptional activation of *Mettl3*, we examined whether YY1 modulates the epigenetic landscape of VSMCs. Co-immunoprecipitation (Co-IP) assays were performed to assess active histone marks, such as H3K4me2, H3K4me3, and H3K27ac, as well as the repressive mark H3K27me3, in aortic media (*Figure 6A* and *B*). Among these, YY1 was found to particularly enrich H3K4me3 binding to DNA in VSMCs (*Figure 6A*). Next, we combined our YY1 ChIP-seq data from hESC-VSMCs with H3K4me3 ChIP-seq data from human SMCs (ENCODE project consortium^37^) to study gene-specific regulation by YY1 in human VSMCs. We found that YY1 bound to the *METTL3* promoter, adjacent to H3K4me3 histone marks (*Figure 6C*). Considering the close proximity of YY1 binding sites to H3K4me3-enriched regions, we investigated how YY1 mediates H3K4me3 deposition at the *METTL3* promoter. In mammals, at least six Set1-like factors, including Set1A, Set1B, and MLL1-4, exhibit histone methyltransferase activity.^38^ Co-IP assays in murine aortic media showed that YY1 interacted with Set1A and its binding partner, Wrd82 (*Figure 6D*). We further confirmed this interaction in hESC-VSMCs using protein immunoprecipitation with either an anti-YY1 (*Figure 6E*) or anti-Set1A (*Figure 6F*) antibody. Moreover, lentivirus-mediated overexpression of YY1 (*Figure 6G*) enhanced the interaction between YY1 and Set1A (*Figure 6H*). Since the N-terminal domain of Set1A can also interact with RNA polymerase II (pol II),^39^ we hypothesized that YY1 might enrich the transcription initiation complex by forming a YY1-Set1A-RNA pol II complex. Indeed, YY1 was found to directly interact with pol II in hESC-VSMCs (*Figure 6H*).

We further investigated whether the interaction between YY1 and Set1A depends on regulatory DNA. DNase I preferentially cleaves accessible regions of the genome, facilitating the discovery of all classes of cis-regulatory elements, including promoters and enhancers.^40^ To test this, we treated whole-cell extracts with DNase I and performed reciprocal co-IP assays using anti-YY1 and anti-Set1A antibodies. The interaction between YY1 and Set1A persisted in the presence of DNase I, indicating that YY1 associates with Set1A independently of chromatin regulatory elements (see Supplementary material online, *Figure S12*). These findings indicate that the initial YY1-Set1A interaction is not restricted to regions of high chromatin accessibility. However, the subsequent activity of Set1A may modify the chromatin state through H3K4 trimethylation, thereby promoting gene transcription. Such interactions may play a role in maintaining regulatory flexibility and responsiveness across the genome in VSMCs.

Recent studies have suggested that the Wdr82-Set1A/COMPASS **(**WSC) complex is the primary entity for efficient H3K4 trimethylation in mammals.^38,41^ We sought to confirm the function of Set1A in regulating H3K4me3 in VSMCs by knocking down *Set1A* in hESC-VSMCs with siRNA (20 nM) for 48 h. We then performed DNA immunoprecipitation using H3K4me3 antibodies, followed by qPCR targeting the *METTL3* promoter. Our results revealed that H3K4me3 deposition at the *METTL3* promoter was almost completely abolished in hESC-VSMCs treated with *Set1A* siRNA compared to controls (*Figure 6I*), confirming that Set1A is primarily responsible for H3K4 trimethylation in VSMCs. Next, we knocked down *YY1* in hESC-VSMCs by siRNA and performed the same immunoprecipitation experiments at 48 h after knockdown. We found that the deposition of H3K4me3 at the *METTL3* promoter was significantly reduced after *YY1* siRNA treatment (*Figure 6J*), indicating that YY1 is required for H3K4 trimethylation at the *METTL3* promoter, possibly through recruiting the Set1A-RNA pol II complex to chromatin. Moreover, hESC-VSMCs treated with *Set1A* siRNA showed significantly decreased *METTL3* mRNA levels (*Figure 6K*) and reduced *METTL3* promoter DNA immunoprecipitated by YY1 (*Figure 6L*) compared to controls. These findings indicate that Set1A is functionally required for *METTL3* transcription and for stabilizing YY1 binding to the *METTL3* promoter in human VSMCs. Altogether, our results suggest that YY1 may function as a novel co-factor within the Set1A-RNA pol II transcription initiation complex, assisting *METTL3* transcription in VSMCs by epigenetically promoting H3K4 trimethylation at its promoter region.

### 3.7 The YY1/Mettl3 axis in VSMCs regulates blood pressure and protects against hypertension

**Figure 7:**
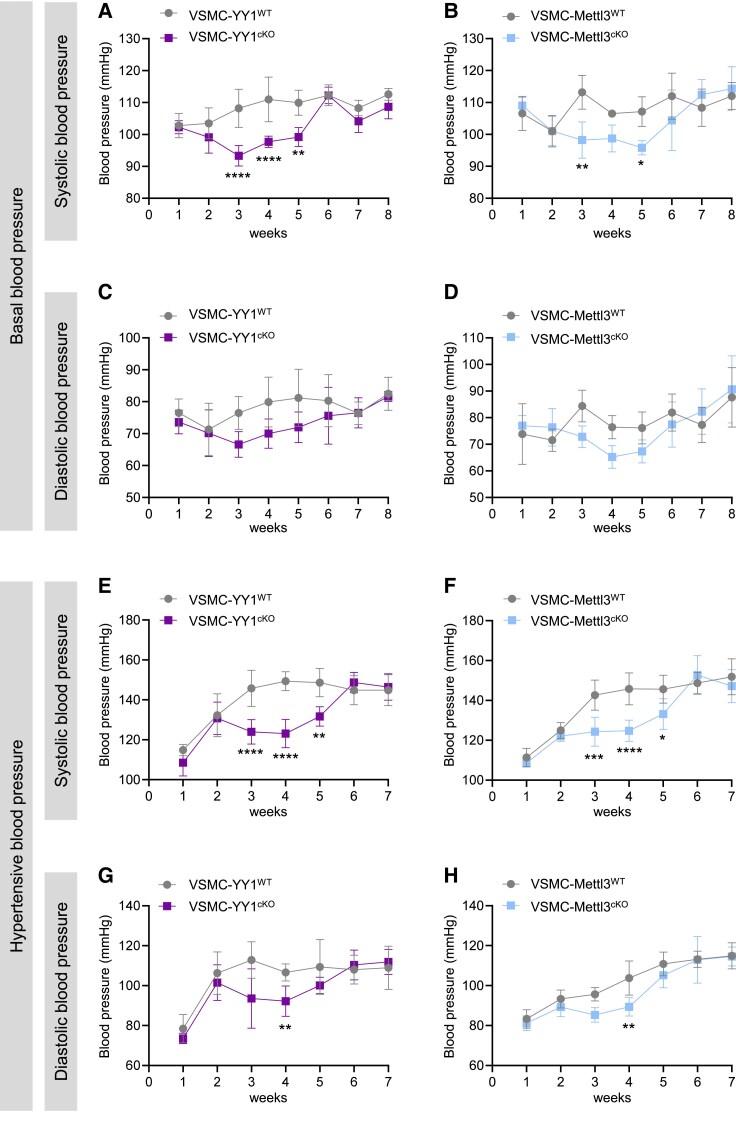
The YY1/Mettl3 axis in VSMCs regulates blood pressure and protects against hypertension. Haemodynamic responses were assessed using the tail-cuff technique. (*A–D*) Basal blood pressure. (*A* and *B*) Systolic blood pressure of VSMC-YY1^cKO^ and VSMC-YY1^WT^ mice (*A*) and VSMC-Mettl3^cKO^ and VSMC-Mettl3^WT^ mice (*B*) was measured weekly from 1 to 8 weeks after Tam administration, six mice per group. (*C* and *D*) Diastolic blood pressure of VSMC-YY1^cKO^ and VSMC-YY1^WT^ mice (*C*) and VSMC-Mettl3^cKO^ and VSMC-Mettl3^WT^ mice (*D*) was similarly measured weekly from 1 to 8 weeks after Tam, six mice per group. (*E–H*) Hypertensive blood pressure: mice were treated with NaCl (0.9%) and L-NAME (0.5 mg/mL) in drinking water for 42 days to induce hypertension. (*E* and *F*) Systolic blood pressure of VSMC-YY1^cKO^ and VSMC-YY1^WT^ mice (*E*) and VSMC-Mettl3^cKO^ and VSMC-Mettl3^WT^ mice (*F*) was measured weekly from 1 to 7 weeks after Tam administration, six mice per group. (*G* and *H*) Diastolic blood pressure of VSMC-YY1^cKO^ and VSMC-YY1^WT^ mice (*G*) and VSMC-Mettl3^cKO^ and VSMC-Mettl3^WT^ mice (*H*) was similarly measured weekly from 1 to 7 weeks after Tam, six mice per group. All quantification data are represented as the mean ± SEM. The *P* values were analysed using two-way repeated measures ANOVA with Sidak’s multiple comparisons. *P < 0.05, **P < 0.01, ***P < 0.001, ****P < 0.0001.

Given that the YY1/Mettl3 axis regulates vasoconstriction in both large and resistance vessels, we explored its role in blood pressure control by monitoring weekly blood pressure measurements over 8 weeks following Tam treatment. Compared to control mice, systolic blood pressure was significantly reduced in VSMC-YY1^cKO^ (*Figure 7A*) and VSMC-Mettl3^cKO^ (*Figure 7B*) mice beginning 3 weeks after Tam treatment, with levels gradually normalizing by 6 weeks. Similarly, diastolic blood pressure also showed a reduced trend in VSMC-YY1^cKO^ (*Figure 7C*) and VSMC-Mettl3^cKO^ (*Figure 7D*) mice at 3 weeks after Tam treatment, also normalizing by 5 weeks. These findings suggest that the YY1/Mettl3 axis plays a key role in blood pressure control and presents a potential therapeutic target. To further assess this axis under hypertensive conditions, we induced hypertension using 0.9% NaCl and L-NAME (0.5 mg/mL) in drinking water. Systolic blood pressure was significantly higher in control mice compared to VSMC-YY1^cKO^ (*Figure 7E*) and VSMC-Mettl3^cKO^ (*Figure 7F*) mice starting 3 weeks after Tam treatment, with levels gradually increasing by 6 weeks. Similarly, diastolic blood pressure was higher in control mice than VSMC-YY1^cKO^ (*Figure 7G*) and VSMC-Mettl3^cKO^ (*Figure 7H*) mice beginning at 3 weeks after Tam treatment, with levels progressing rising by 5 weeks. Taken together, these results highlight the critical role of the YY1/Mettl3 axis in mitigating hypertension and regulating blood pressure under both normal and hypertensive conditions.

## 4. Discussion

To date, the precise biological role of YY1 in VSMCs remains unknown due to a lack of sufficient genetic evidence from *in vivo* loss-of-function studies. Here, we demonstrate that the loss of YY1 in VSMCs significantly compromises vascular contraction. YY1 does not directly regulate VSMC proliferation nor survival. Further analysis shows that YY1 does not influence the expression of Ca^2+^ and K^+^ ion channels, nor does it affect Ca^2+^ entry, even after exposure to Phe or a Ca^2+^ ionophore. Moreover, Ca^2+^ entry through voltage-operated Ca^2+^ channels after KCl-mediated membrane depolarization, or supplementation with exogenous CaCl_2_, does not rescue the contractile function of blood vessel. These findings indicate that YY1 does not directly regulate membrane depolarization in VSMCs. We further elucidate the molecular mechanisms by which YY1 controls VSMC contractile function through genome-wide RNA-seq, YY1 ChIP-seq, and luciferase reporter assays. Our results identify YY1 as a transcriptional activator of *Mettl3*, directly binding to its promoter regions. In addition, m^6^A-seq analysis of GGACG and GGAGA motifs shows reduced m^6^A deposition, particularly at the CDS, stop codons, and 3′UTRs of mRNAs in YY1-deficient VSMCs. Specifically, the transcript stability of *Mylk2*, *Tgfb2*, and *Myh11* is regulated by Mettl3-mediated m^6^A RNA methylation. Expression of MLCK, TGFβ2, and the phosphorylation of their target proteins, RLC and Smad3, was restored after AAV-mediated VSMC-specific delivery of *Mettl3* in VSMC-YY1^cKO^ mice. Similarly, the expression of MYH11 was also rescued. Importantly, the impaired vasoconstriction observed in the blood vessels of VSMC-YY1^cKO^ mice was restored after *Mettl3* gene delivery. Collectively, these findings highlight a novel regulatory axis in which YY1 and Mettl3 maintain the contractile function of VSMCs through promoting the mRNA stability and, therefore, translational efficiency of *MYLK2*, *TGFβ2*, and *MYH11* by m^6^A RNA modifications.

In the current work, we also reveal a potential epigenetic mechanism through which YY1 regulates *Mettl3* transcription. We provided multiple lines of evidence to prove that YY1 acts as a regulator of the transcriptional initiation complex WSC-RNA pol II in VSMCs and is essential for the recruitment of this complex to chromatin, thereby promoting the transcriptional activation of *Mettl3*. Within this complex, Set1A primarily mediates H3K4 trimethylation, which facilitates gene activation at promoters or transcription start sites.^38^ Moreover, both Wdr82^38^ and Set1,^39^ via its N-terminal domain, can directly bind to RNA pol II to initiate transcription. Our Co-IP data showed that YY1 interacts with Set1A, Wrd82, and RNA pol II in primary murine aortic VSMCs and hESC-VSMCs. In addition, YY1 and H3K4me3 ChIP-seq results demonstrated overlapping peaks at the promoter region of the *Mettl3* gene in human VSMCs. Knockdown of *Set1A* in hESC-VSMCs significantly reduced H3K4me3 at the *METTL3* promoter and decreased *METTL3* mRNA expression, highlighting the importance of H3K4 trimethylation in *METTL3* transcription. Similarly, knockdown of *YY1* led to a marked reduction in H3K4me3 at the *METTL3* promoter, possibly due to reduced recruitment of the WSC-RNA pol II complex to the promoter. Furthermore, knockdown of *Set1A* also significantly reduced the binding of YY1 to the *METTL3* promoter, suggesting that the stability of the YY1-WSC-RNA pol II complex is critical for efficient *METTL3* transcription. These findings establish YY1 as a peripheral component of the transcriptional initiation complex WSC-RNA pol II in VSMCs. Based on these observations, we hypothesize that in the absence of YY1, the WSC-RNA pol II complex is less stable at the *METTL3* promoter, H3K4 trimethylation becomes insufficient, and *METTL3* transcription is less efficient. It is also noteworthy that we utilized contractile hESC-VSMCs for *in vitro* experiments, as the commonly used human VSMC cell line HASMC displayed synthetic features, characterized by proliferation and minimal MLCK and MYH11 expression, which are key targets in our studies. We, therefore, demonstrate that hESC-VSMCs represent a better alternative for modelling quiescent, contractile VSMCs.

In smooth muscle, MLCK promotes contraction of actin filaments by activating myosin ATPase activity, with or without phosphorylation of RLC.^42^ TGFβ is also a potent inducer of the contractile phenotype in VSMCs, driving the expression of contractile genes. Mechanistically, TGFβ initiates a receptor kinase cascade that phosphorylates Smad3, which in turn activates transcription by binding to the CArG elements present in the promoter regions of contractile genes, such as SM22α,^28^ αSMA,^29^ and Myh11.^36^ In addition, TGFβ enhances vessel wall strength by promoting collagen synthesis in VSMCs.^43^ The reduced expression of genes involved in collagen and ECM organization observed after genetic ablation of *Yy1* in VSMCs may be attributed to reduced TGFβ signalling. In addition to *Mylk2* and *Tgfb2*, we also observed that the significantly reduced MYH11 protein levels in VSMC-YY1^cKO^ mice, measured 4 weeks after Tam induction, were restored after AAV-Mettl3 treatment. MYH11, a major component of the contractile apparatus in VSMCs, is essential for maintaining both contractile function and structural stability. In addition to TGFβ/Smad3 signalling, we showed that MYH11 expression is also regulated by Mettl3-mediated m^6^A RNA modifications. Considering the essential roles of MLCK, TGFβ2, and MYH11 in preserving the contractile function of VSMCs, our findings uncover a novel regulatory mechanism in which the YY1/Mettl3 axis epigenetically regulates vascular contraction and peripheral resistance. By controlling mRNA stability, the YY1/Mettl3 axis modulates dynamic blood pressure changes through its effects on vascular resistance. In our study, long-term blood pressure monitoring revealed a decrease in blood pressure between 3 and 5 weeks after Tam administration, followed by a gradual increase, which appears to be independent of YY1/Mettl3 expression. Notably, vascular contraction measurements in mesenteric vessels at 10 weeks after Tam treatment still showed significantly reduced vasoconstriction in these mice. This gradual adaptation in blood pressure is likely due to compensatory mechanisms, as blood pressure is influenced by multiple factors, including blood volume, cardiac output, kidney function, and sympathetic nervous system activity, among others. One limitation of the current study is the lack of access to radiotelemetry. Radiotelemetry allows us to measure blood pressure continuously in conscious, freely moving mice, eliminating the potential stress-induced artefacts associated with tail-cuff measurements. Nevertheless, it has been recently commented that the tail-cuff method remains a reliable approach, particularly when focusing on average blood pressure within a sufficient number of animals.^44^ In this study, we ensured the robustness of our findings by using an adequate sample size and carefully controlling experimental conditions. Future experiments utilizing radiotelemetry would help further validate and extend our findings perhaps on circadian blood pressure patterns and dynamic changes over time. Despite this limitation, our results demonstrate that the YY1/Mettl3 axis plays a pivotal role in regulating vascular resistance and blood pressure dynamics. It contributes to the development and progression of blood pressure-related disorders, including hypertension, thereby highlighting its potential as a promising therapeutic target.

**Figure 8:**
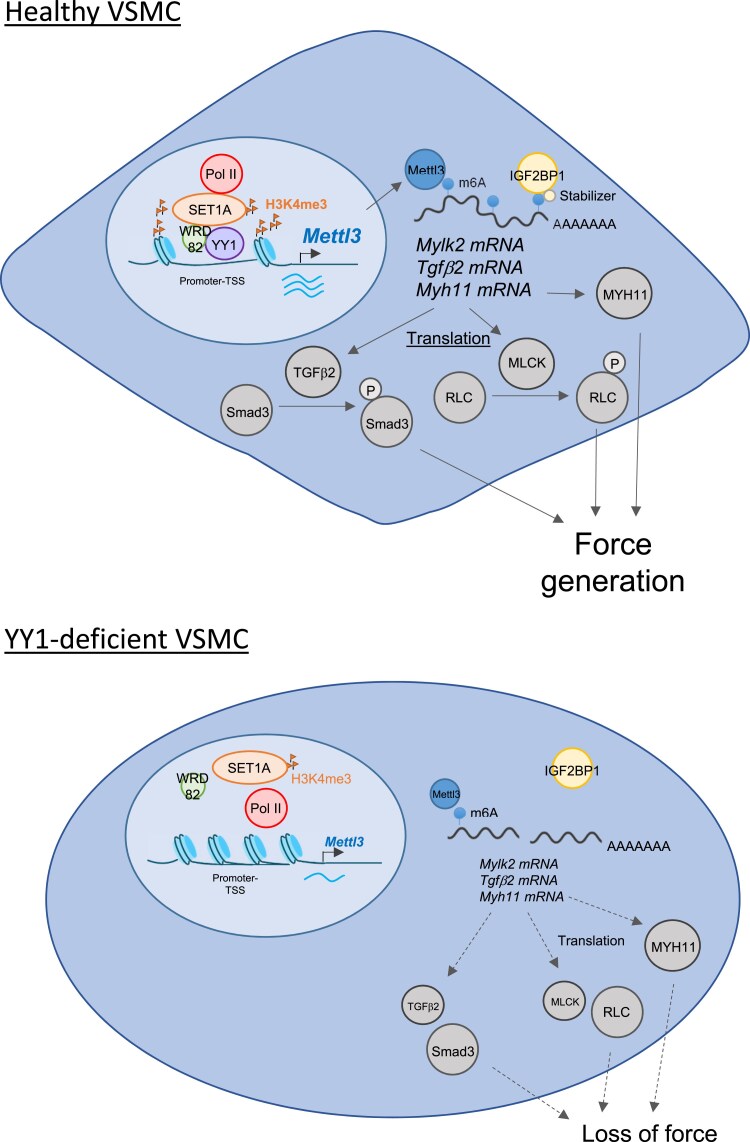
An epigenetic model by which YY1 regulates the contractile function of VSMCs. In healthy VSMCs, YY1 activates the transcription of *Mettl3* by recruiting Set1A for H3K4 trimethylation and stabilizing the transcription initiation complex Set1A/Wrd82-RNA pol II at the promoter region. Mettl3 mediates m^6^A RNA methylation in VSMCs, enhancing the stability and translation efficiency of its mRNA targets, including *Mylk2*, *Tgfb2*, and *Myh11*, possibly through the m^6^A reader IGF2BP1. As a result, MYH11 forms part of the contractile apparatus. MLCK (encoded by *Mylk2*) and TGFβ2 facilitate phosphorylation of RLC and Smad3, respectively, which further activate downstream machineries essential for vessel contraction and force generation. On the other hand, in YY1-deficient VSMCs, transcription of *Mettl3* is inhibited. This leads to accelerated decay of *Mylk2*, *Tgfb2*, and *Myh11* transcripts, resulting in reduced vessel contractility.

Recent genetic analysis of over a million individuals has identified YY1 as one of the novel loci associated with blood pressure traits.^21^ However, it has not yet been experimentally demonstrated that YY1 contributes to the regulation of vasoreactivity and blood pressure. Our study provides what may be the first evidence highlighting the importance of YY1 as a regulator of vessel contraction and vascular resistance. Taken together, we unravel a novel epigenetic mechanism by which YY1 regulates the contractile function of VSMCs (*Figure 8*). In healthy VSMCs, YY1 activates the transcription of *Mettl3* by recruiting Set1A to mediate H3K4 trimethylation and stabilizing the transcription initiation complex Set1A/Wrd82-RNA pol II at the *Mettl3* promoter region. Mettl3 mediates m^6^A RNA methylation in VSMCs, enhancing the stability and translation efficiency of its mRNA targets, including *Mylk2*, *Tgfb2*, and *Myh11*, possibly through the m^6^A reader IGF2BP1. As a result, MLCK and TGFβ2 mediate the phosphorylation of RLC and Smad3, respectively, activating downstream machineries that maintain the contractile function of VSMCs. MYH11 expression is stabilized, providing structural support and maintaining vascular homeostasis. On the other hand, in YY1-deficient VSMCs, transcription of *Mettl3* is inhibited, leading to faster decay of *Mylk2*, *Tgfb2*, and *Myh11* transcripts and a subsequent loss of contractility. Our findings give novel insights into the epigenetic control of epitranscriptomics in VSMCs, which promotes vascular contraction through the YY1/Mettl3 regulatory axis. Moreover, our results highlight the clinically relevant role of the YY1/Mettl3 axis in mitigating hypertension and regulating blood pressure under both normal and hypertensive conditions.

## Supplementary Material

cvaf136_Supplementary_Data
