# Supplementary material for: YY1 regulates vascular resistance and blood pressure dynamics through epigenetic control of m6A RNA modifications in vascular smooth muscle cells
Source: Cardiovasc Res. 2025 Aug 7;121(12):1898–916. doi: 10.1093/cvr/cvaf136 (PMC12551392; doi:10.1093/cvr/cvaf136)
Supplement: cvaf136_Supplementary_Data [file cvaf136_supplementary_data.zip › Supplemental unedited gel 20250222 R2.pdf]

Unedited gels to corresponding figures

Fig 2H

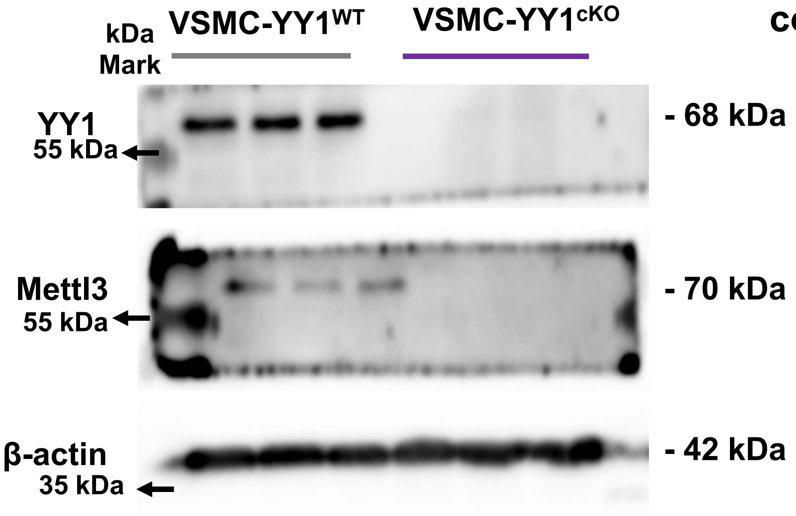

Fig 3O

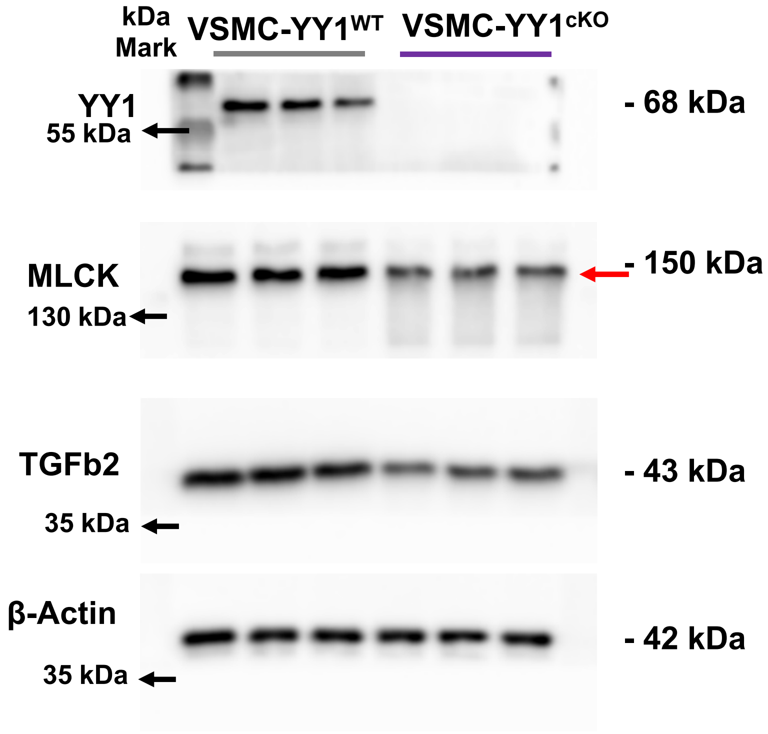

Fig 3R

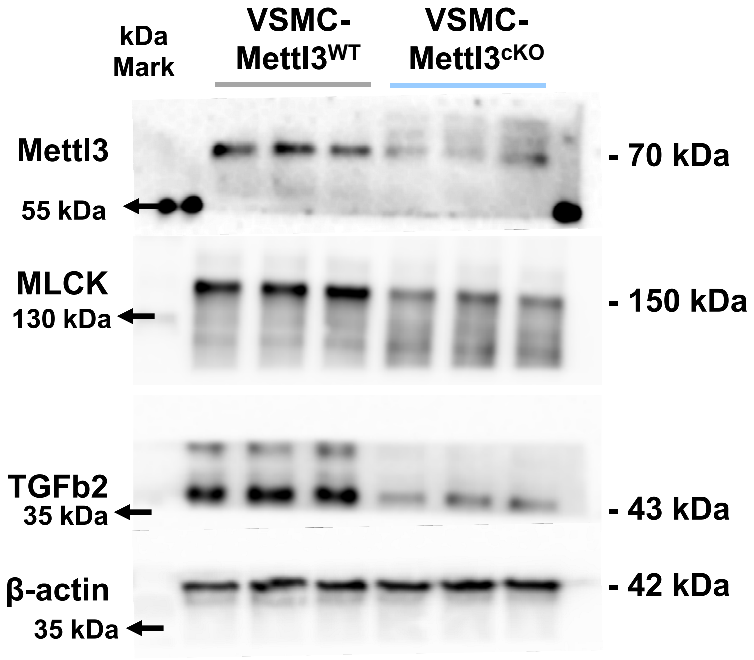

Unedited gels to corresponding figures

Fig 5A

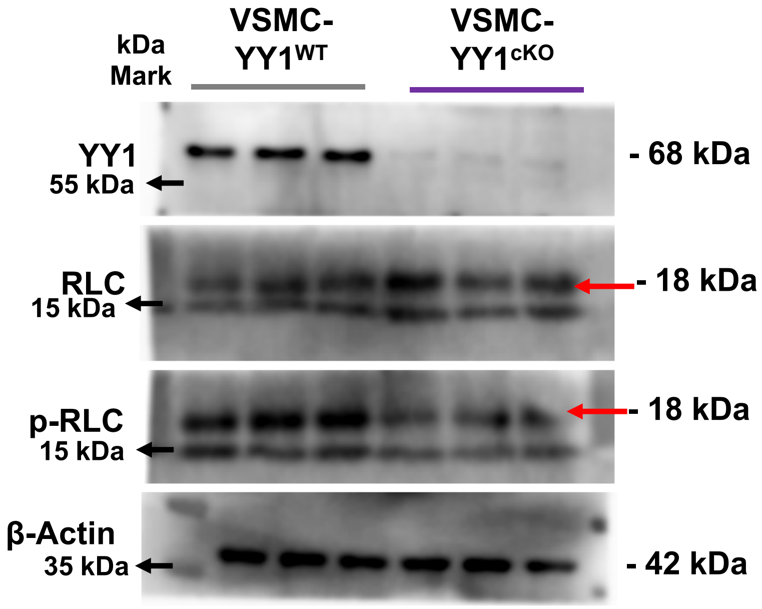

Fig 5B

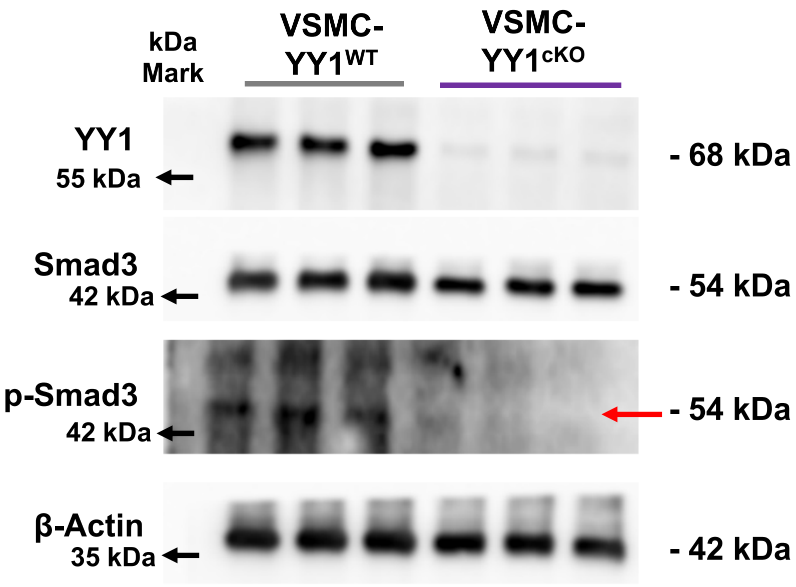

Unedited gels to corresponding figures

Fig 5C

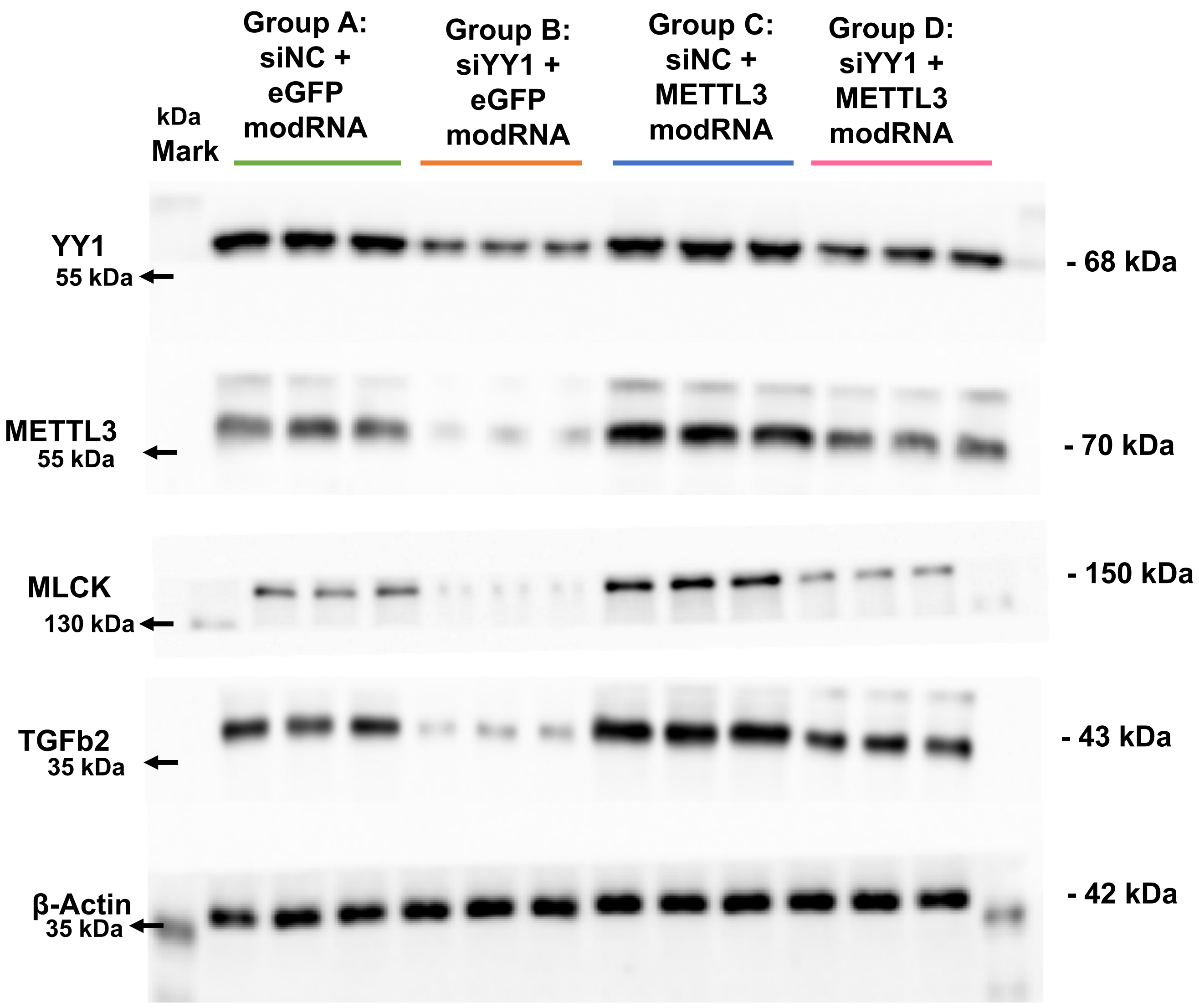

Unedited gels to corresponding figures

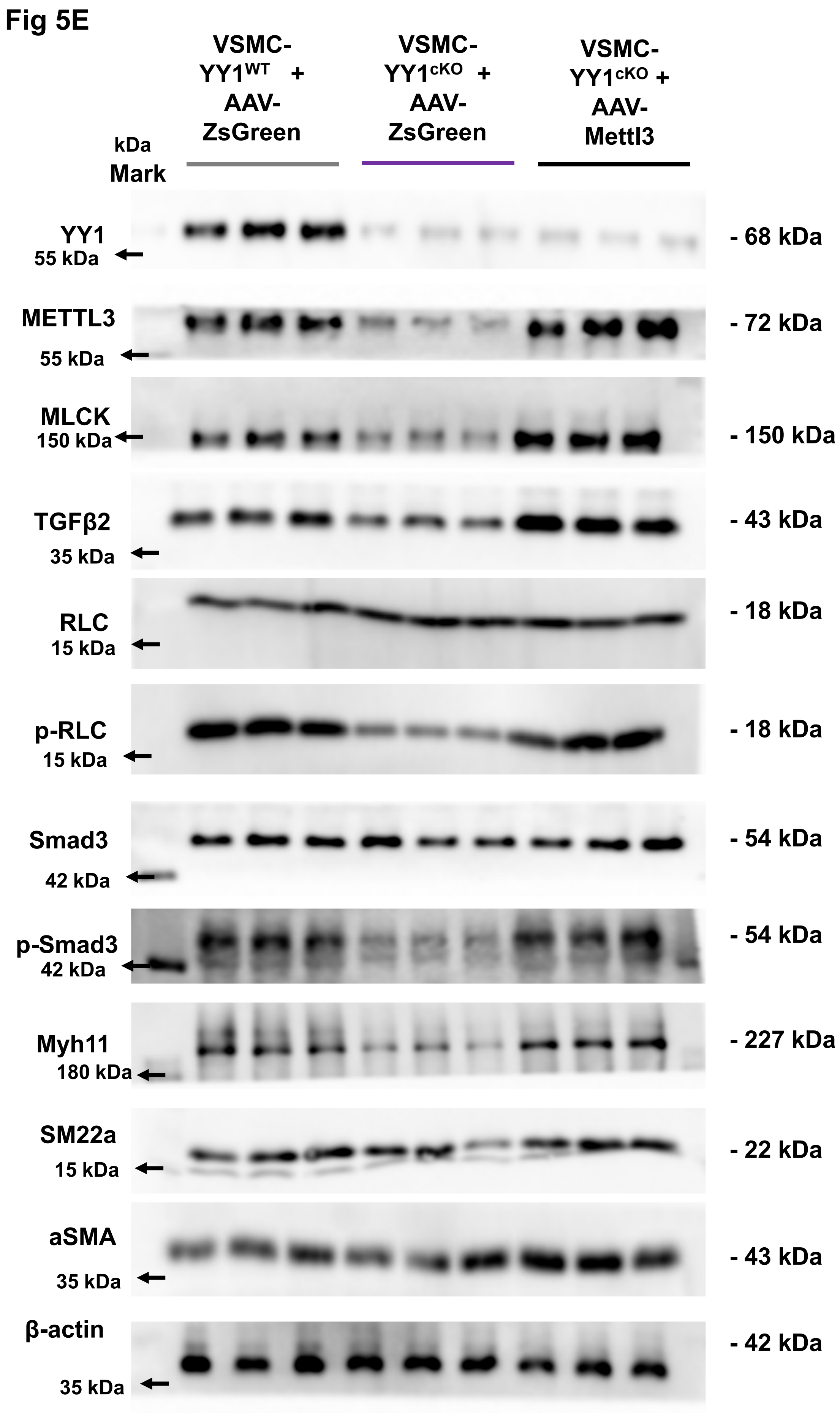

Unedited gels to corresponding figures

Fig 6A

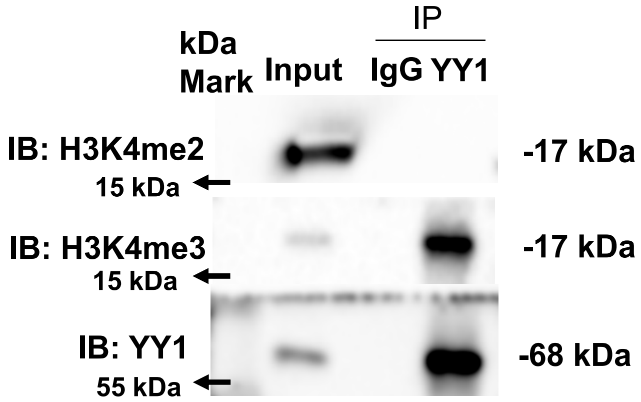

Fig 6B

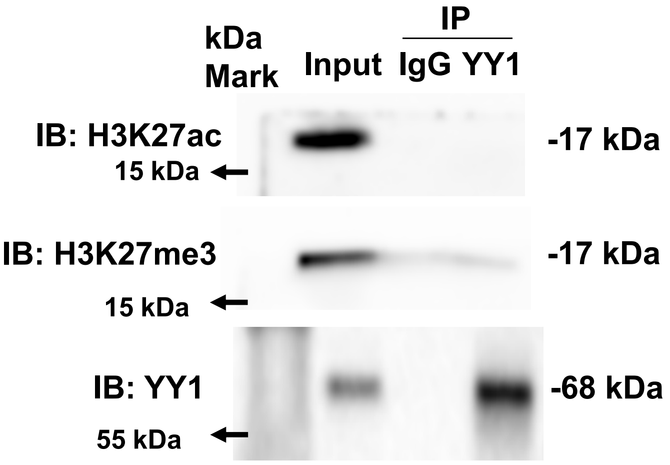

Fig 6D

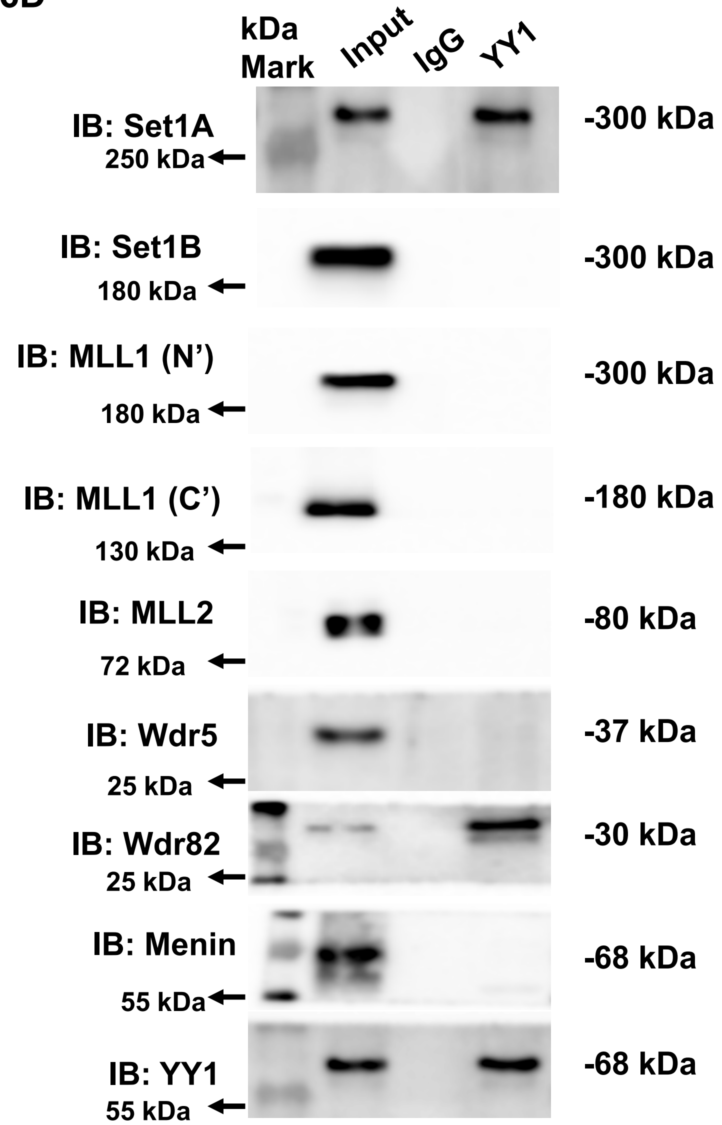

Unedited gels to corresponding figures

Fig 6E

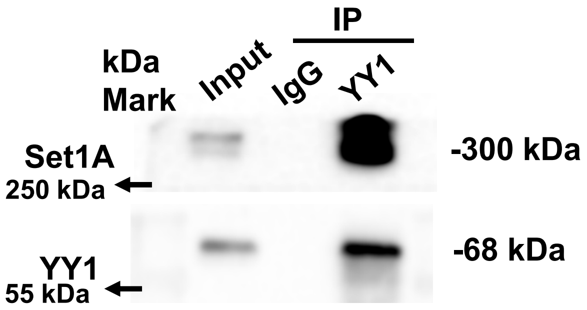

Fig 6F

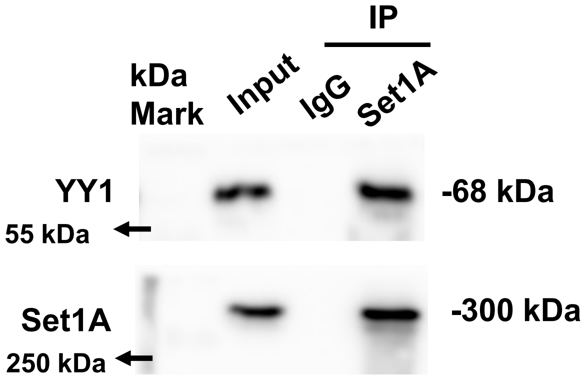

Fig 6G

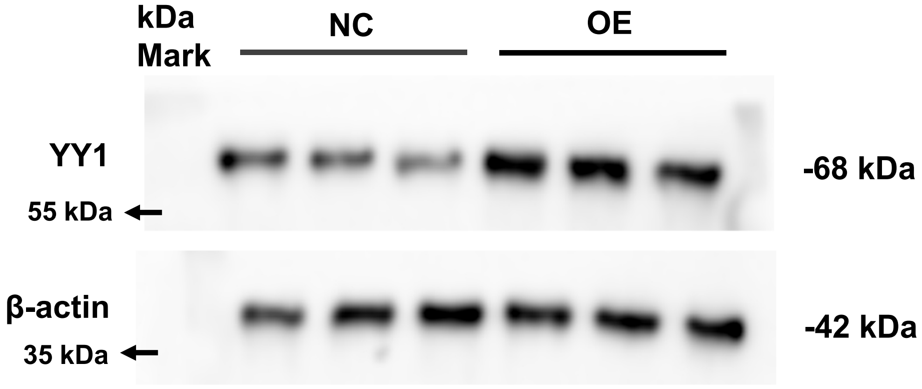

Fig 6H

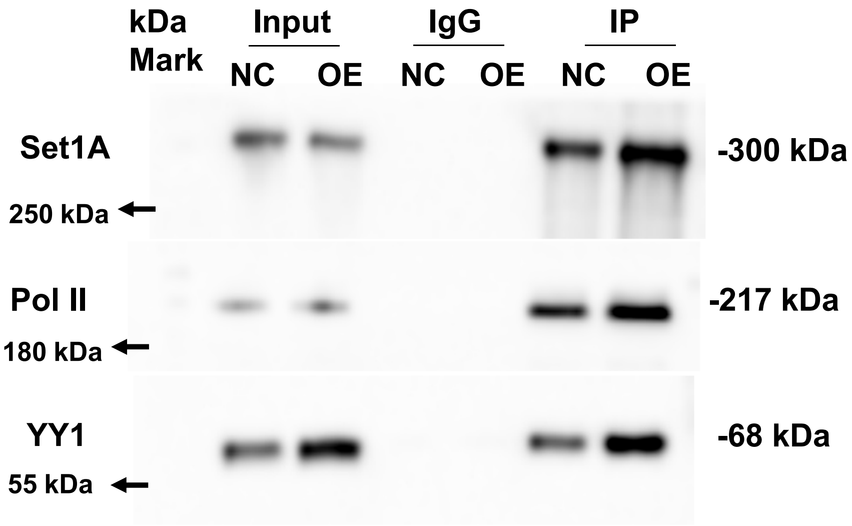

Unedited gels to corresponding figures

Fig S4F

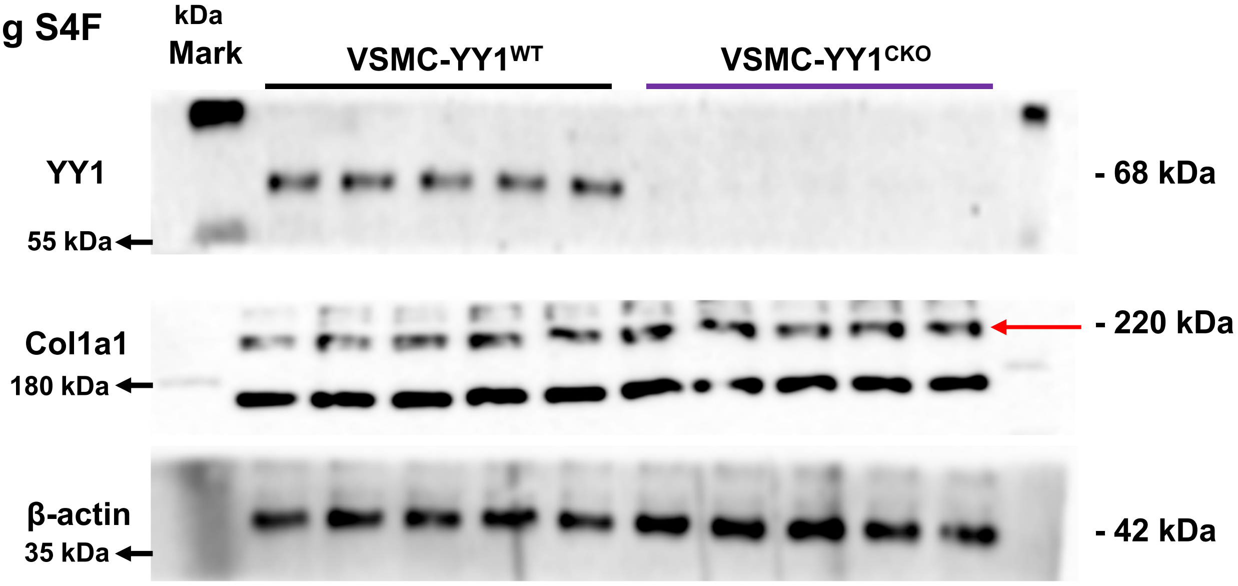

Fig S5A

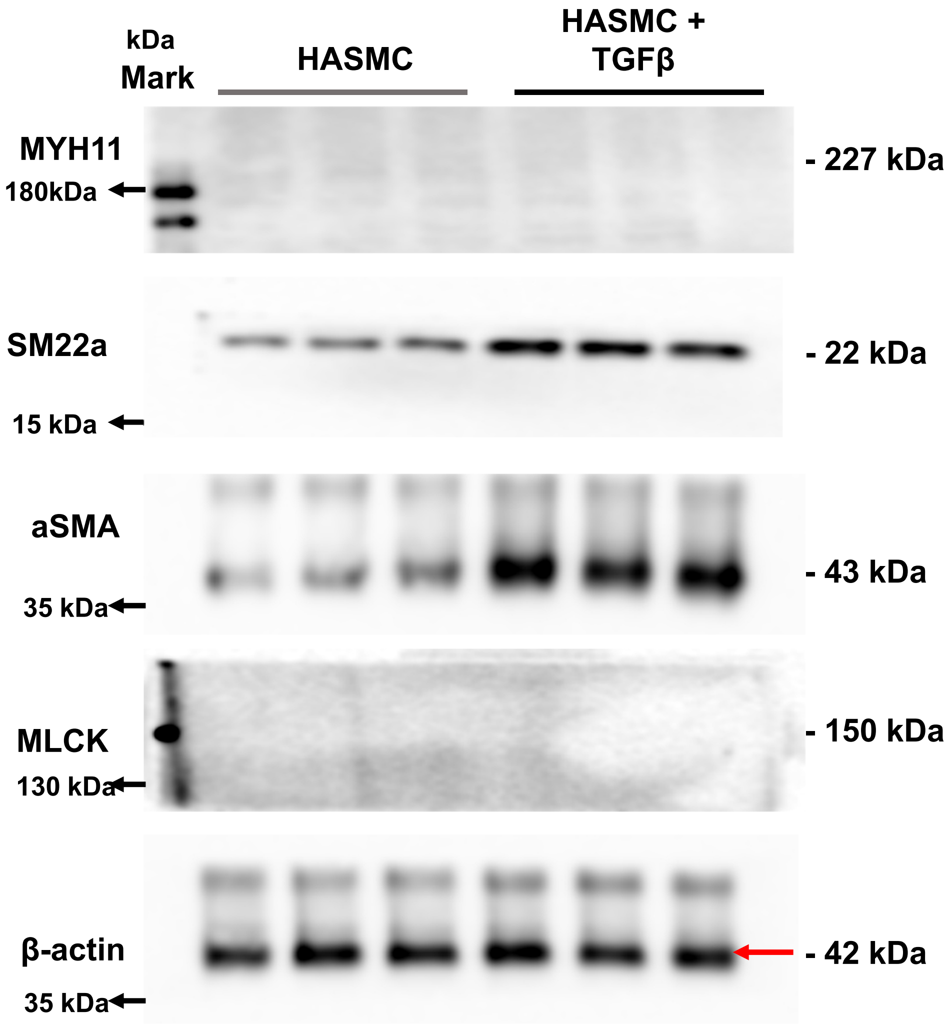

Unedited gels to corresponding figures

Fig S5C

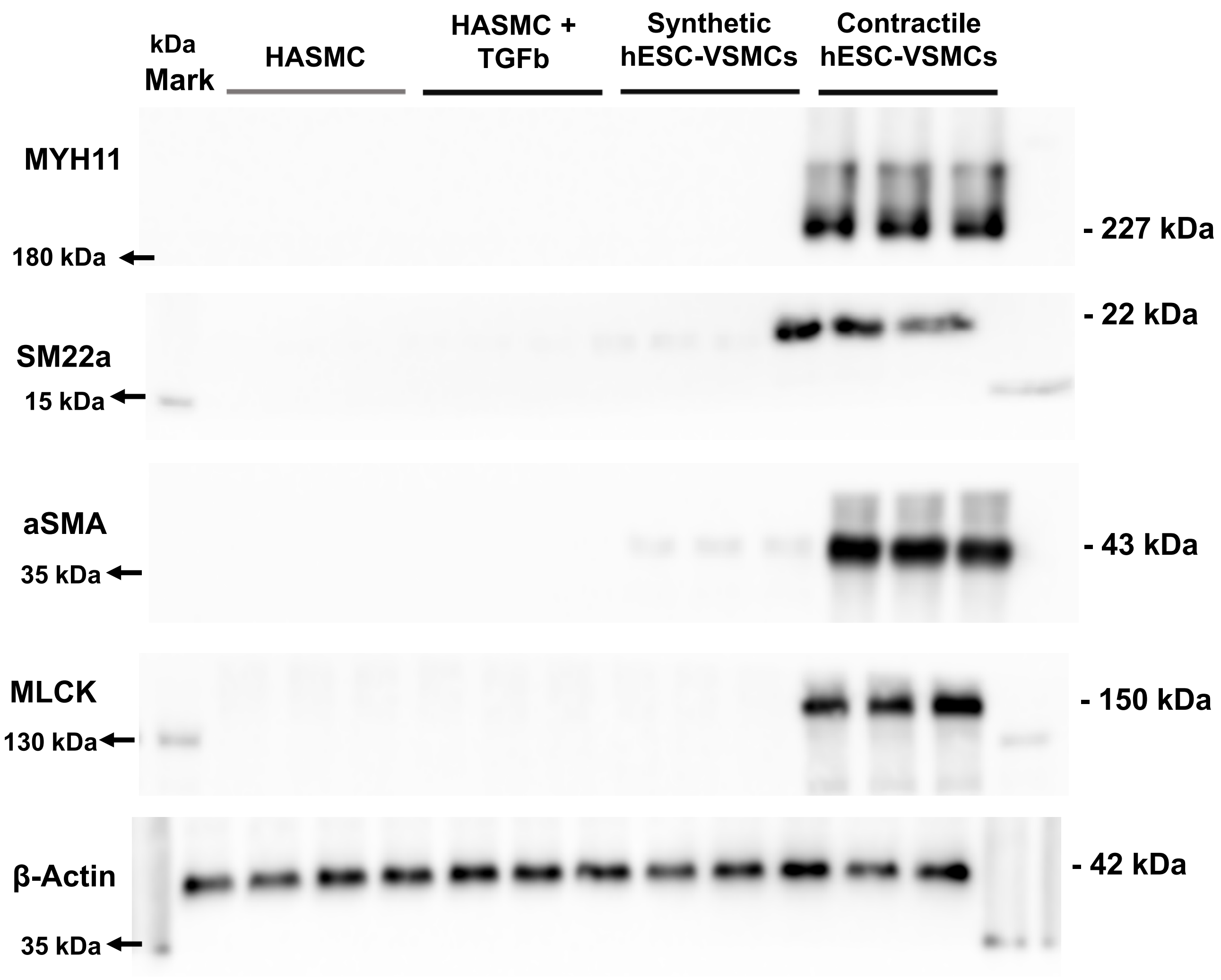

Fig S9A

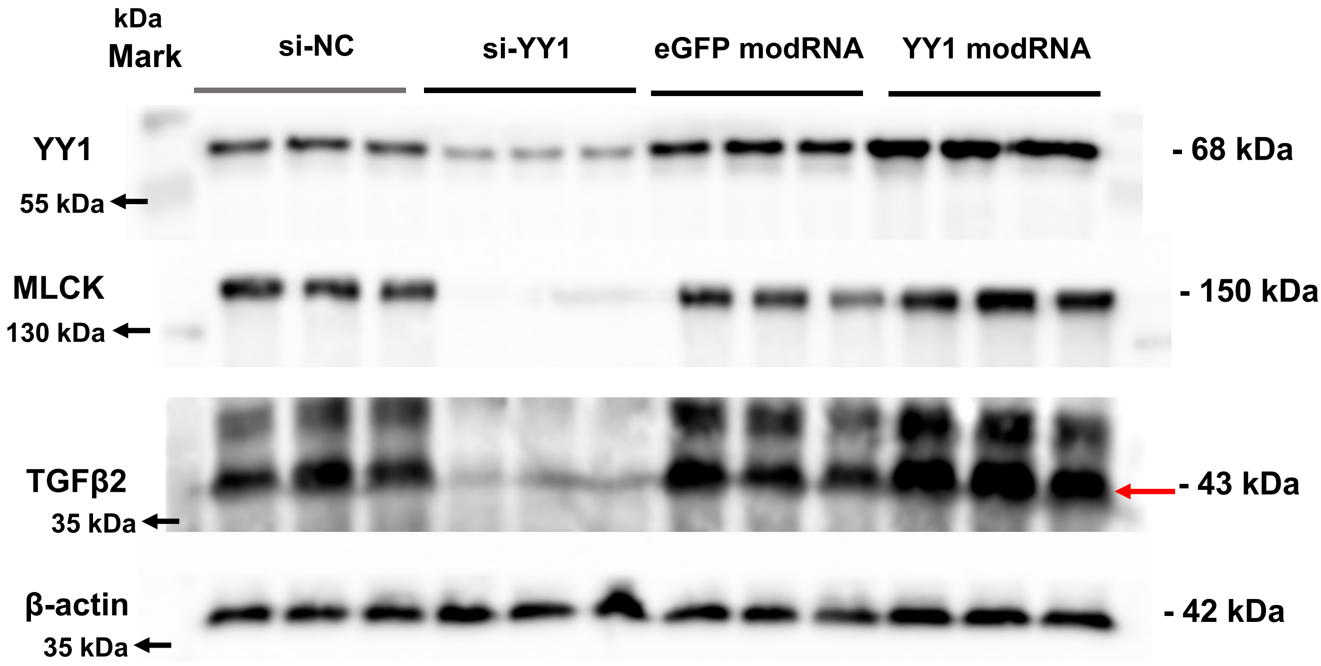

Unedited gels to corresponding figures

Fig S10A

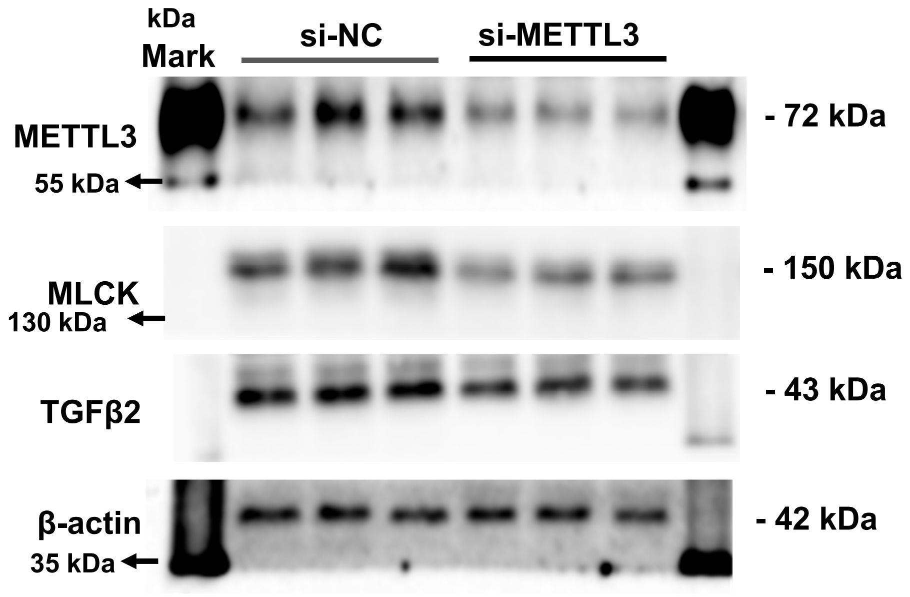

Fig S10B

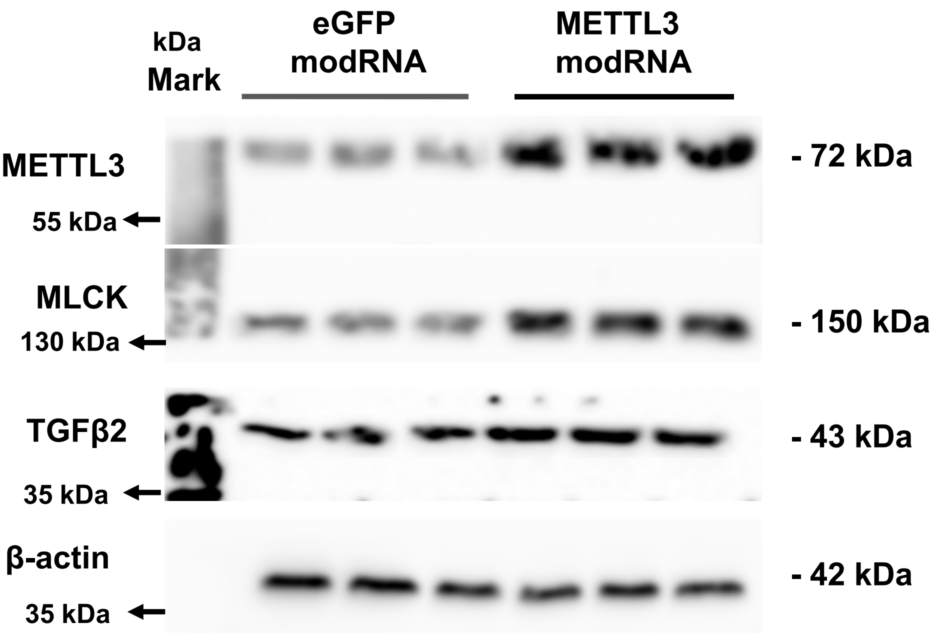

Unedited gels to corresponding figures

Fig S12A

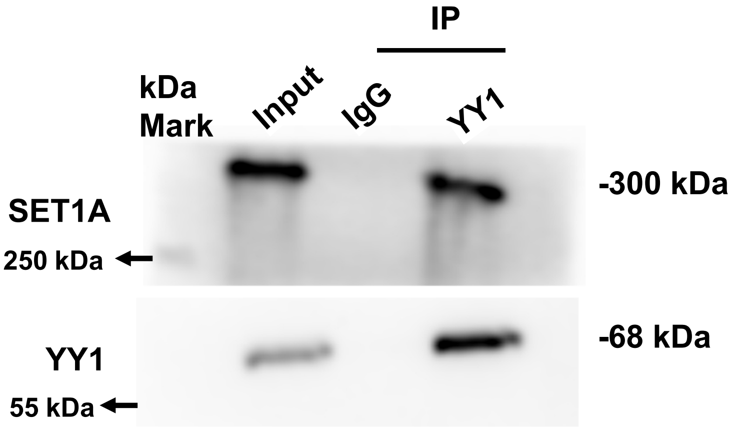

YY1 pulldown

Fig S12B

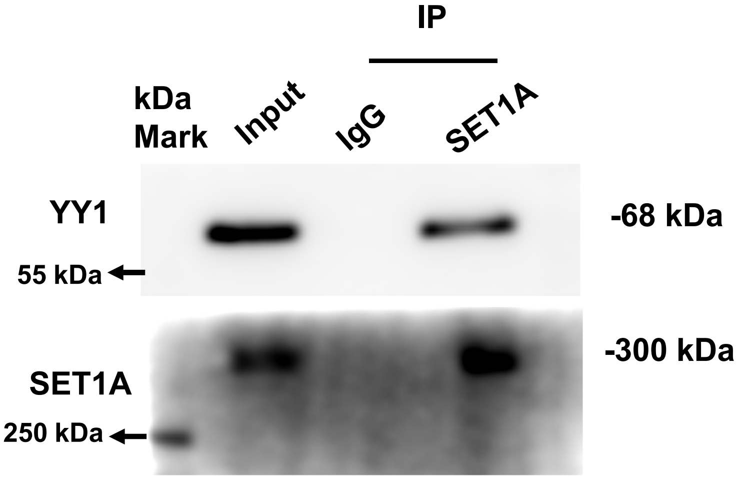

SET1A pulldown
