# Supplementary material for: YY1 regulates vascular resistance and blood pressure dynamics through epigenetic control of m6A RNA modifications in vascular smooth muscle cells
Source: Cardiovasc Res. 2025 Aug 7;121(12):1898–916. doi: 10.1093/cvr/cvaf136 (PMC12551392; doi:10.1093/cvr/cvaf136)
Supplement: cvaf136_Supplementary_Data [file cvaf136_supplementary_data.zip › Supplementary_final.pdf]

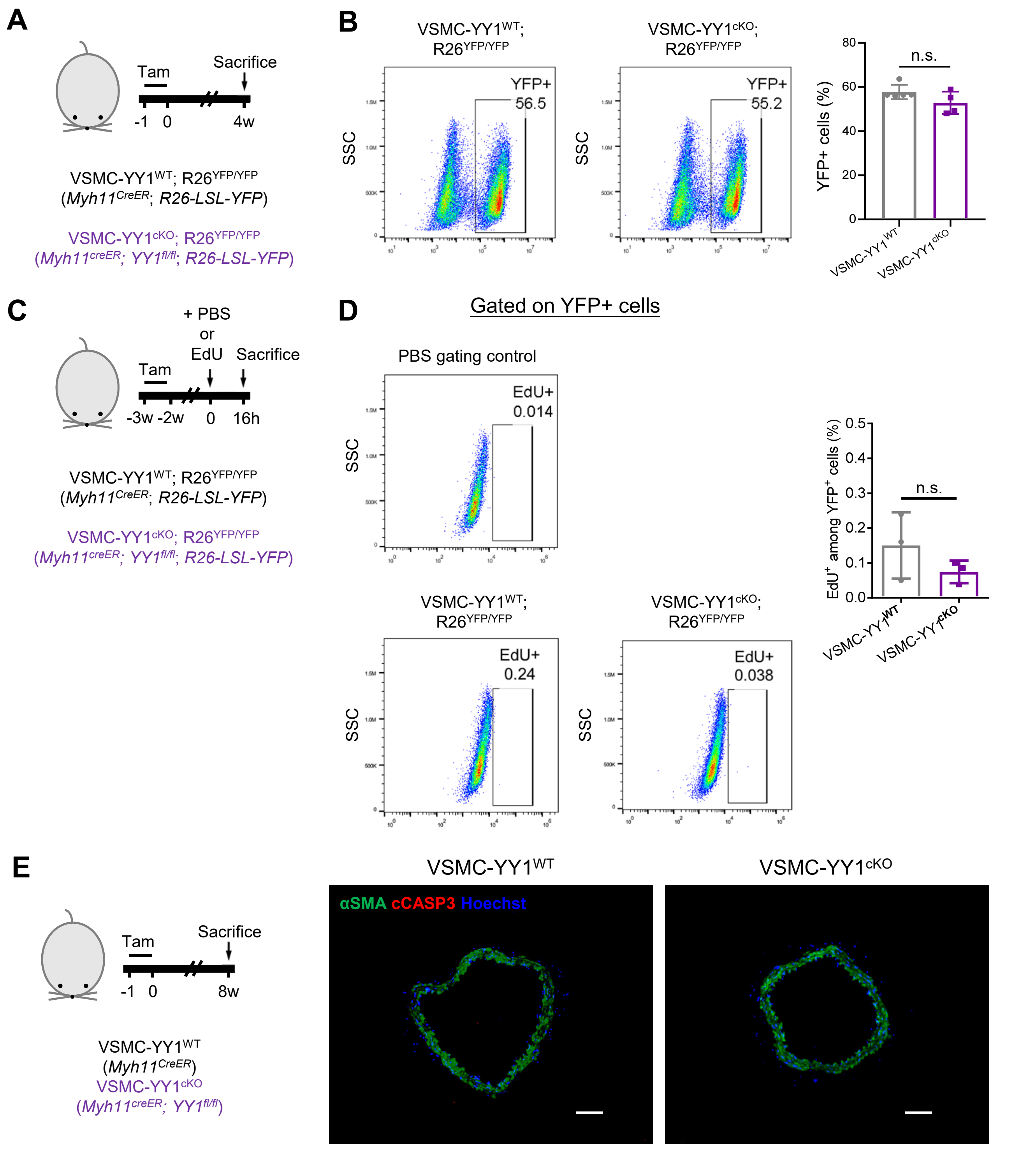

Figure S1 YY1 does not regulate VSMC proliferation and survival *in vivo*. (A) A schematic showing the experimental design and the generation of Myh11-CreER (VSMC-YY1^{WT});R26$^{YFP/YFP}$ and Myh11-CreER;Yy1$^{fl/fl}$ (VSMC-YY1^{cKO});R26$^{YFP/YFP}$ mice. (B) Quantification by flow cytometry showing the percentage of VSMCs expressing YFP in the aorta of VSMC-YY1^{WT};R26$^{YFP/YFP}$ and VSMC-YY1^{cKO};R26$^{YFP/YFP}$ at 4 weeks after tamoxifen (Tam) treatment, 5 vessels per group. (C) A schematic showing the experimental design. (D) Quantification by flow cytometry showing the percentage of VSMCs expressing YFP and EdU in the aorta of VSMC-YY1^{WT}; R26$^{YFP/YFP}$ and VSMC-YY1^{cKO}; R26$^{YFP/YFP}$ at 2 weeks after Tam treatment, n=3 per group. EdU was intraperitoneally injected and the aortas were harvested at 16 hours after EdU injection. (E) Immunostaining on frozen sections for alpha smooth muscle actin (aSMA, green), cleaved caspase 3 (cCASP3, red) and Hoechst (blue) in the aorta of VSMC-YY1^{WT} and VSMC-YY1^{cKO} at 8 weeks after Tam, scale bars: 100 um, n=5. All quantification data are represented as the mean ± SEM. *P* values were calculated by Student's *t*-tests.

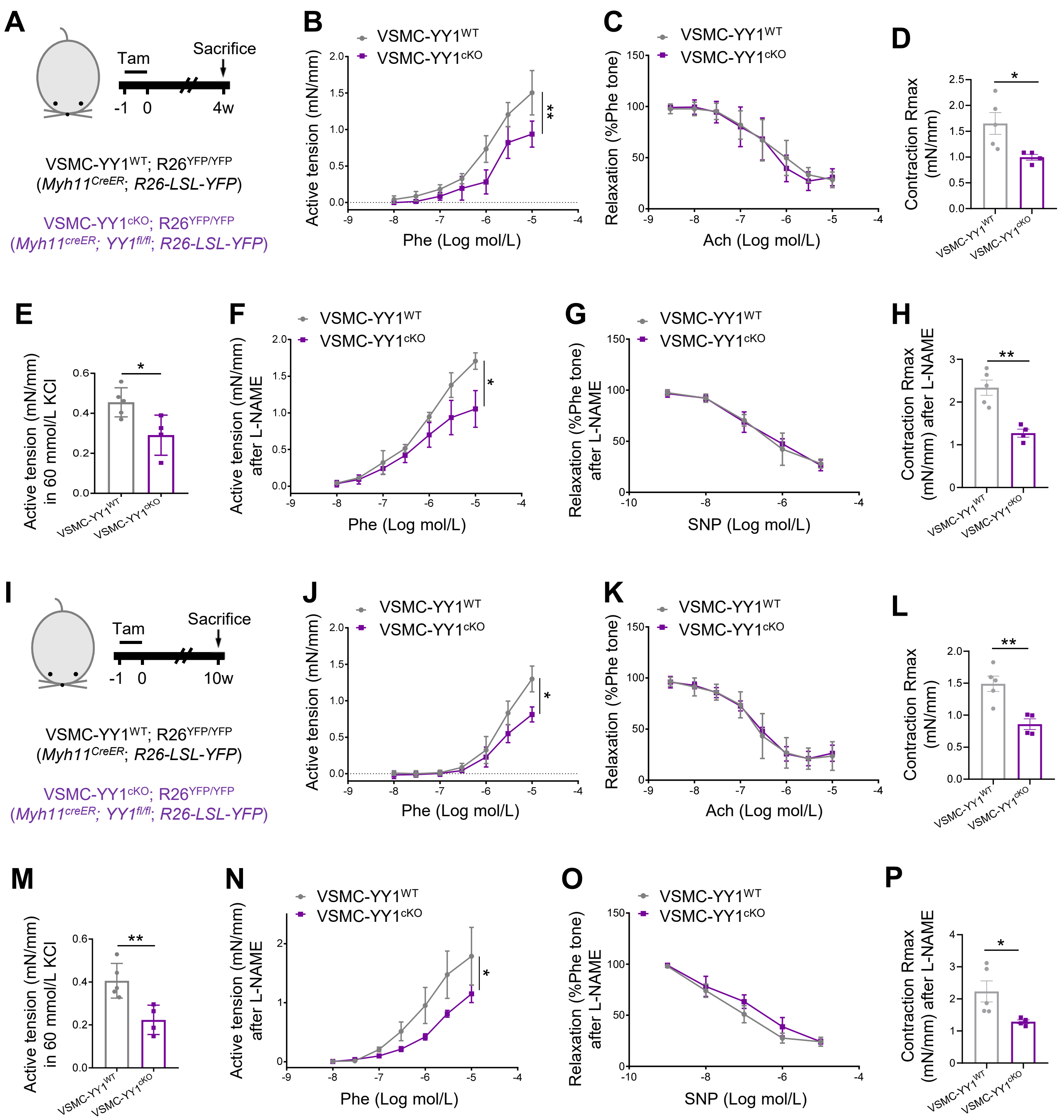

Figure S2 Loss of YY1 in VSMCs impairs mesenteric vessel contraction *in vivo*. (A) A schematic showing the experimental design and the generation of Myh11-CreER (VSMC-YY1^{WT});R26$^{YFP/YFP}$ and Myh11-CreER;Yy1$^{fl/fl}$ (VSMC-YY1^{cKO});R26$^{YFP/YFP}$ mice. (B) Concentration-dependent vasoconstriction responses of mouse mesenteric vessels to phenylephrine (Phe), and (C) concentration-dependent relaxation responses to acetylcholine (Ach) were measured in VSMC-YY1^{cKO} and VSMC-YY1^{WT} mice 4 weeks after tamoxifen (Tam) treatment, 4-5 vessels per group. (D) The maximum responses (Rmax) in vasoconstrictions and (E) 60 mM KCl-mediated contractile responses were examined, 4-5 vessels per group. (F) Endothelium-independent vasoconstrictions and (G) relaxations were evaluated in the presence of the nitric oxide synthase inhibitor L-NAME, 4-5 mesenteric vessels per group. (H) Rmax in vasoconstrictions after L-NAME. (I) A schematic showing the experimental design. (J) Concentration-dependent vasoconstriction responses of mouse mesenteric vessels to phenylephrine (Phe), and (K) concentration-dependent relaxation responses to acetylcholine (Ach) were measured in VSMC-YY1^{cKO} and VSMC-YY1^{WT} mice 10 weeks after Tam treatment, 4-5 vessels per group. (L) Rmax in vasoconstrictions or (M) 60 mM KCl-mediated contractile responses were examined, 4-5 mesenteric vessels per group. (N) Endothelium-independent vasoconstrictions and (O) relaxations were evaluated in the presence of L-NAME, 4-5 vessels per group. (P) Rmax in vasoconstrictions after L-NAME. All quantification data are represented as the mean ± SEM. *P* values were calculated by Student's *t*-tests. Statistics for concentration-response relationships were performed by calculating the area under curve followed by *t*-test analysis. $^{*}p<0.05$, $^{**}p<0.01$.

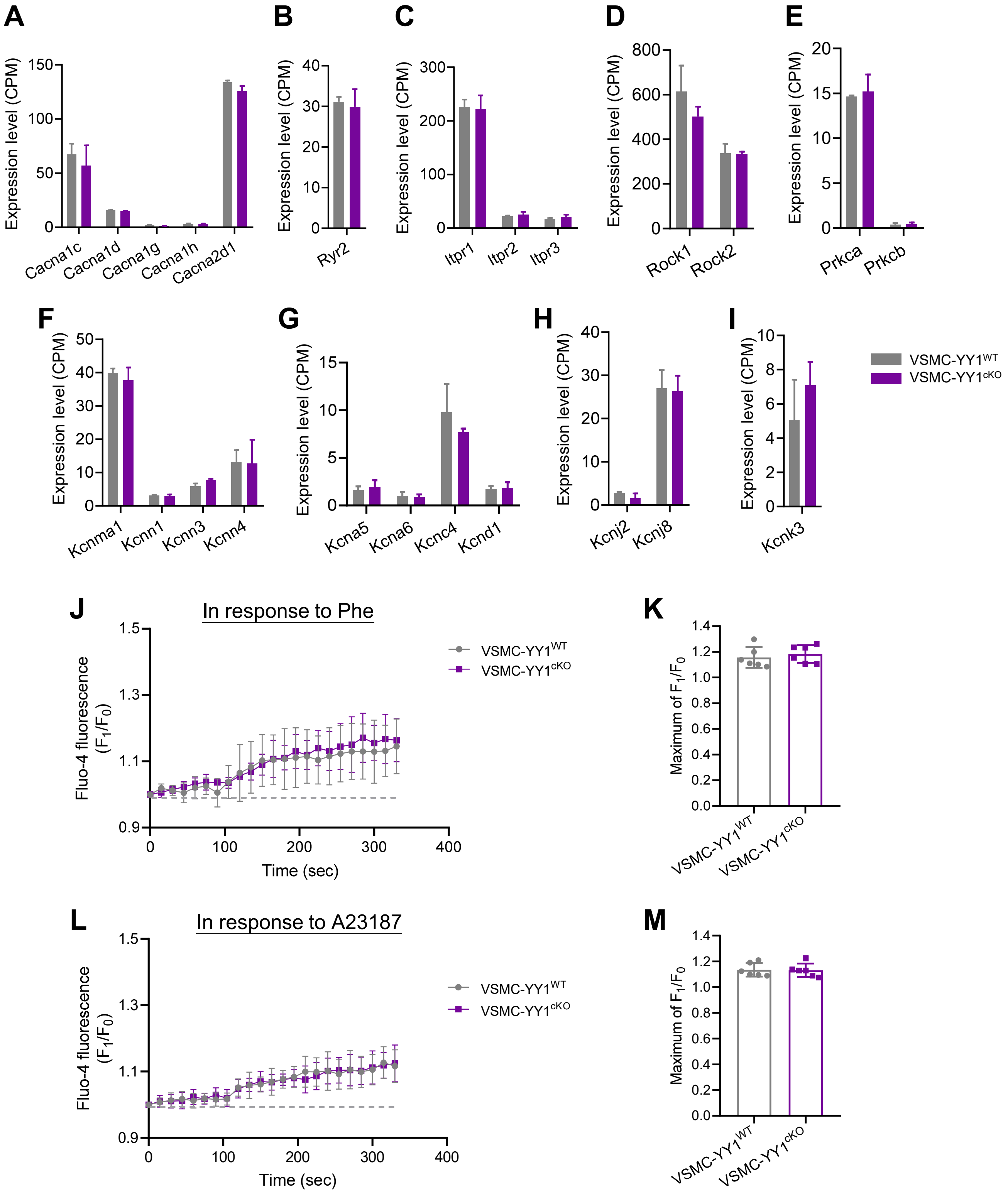

Figure S3 YY1 does not regulate ion channel expression and Ca^{2+} entry into VSMCs. (A-I) Bulk RNA-seq were performed using aortic media derived from VSMC-YY1^{WT} (3 vessels) and VSMC-YY1^{cKO} (2 vessels) at day 7 after Tam. Expression levels showing voltage gated calcium channels (A); ryanodine receptor (B); calcium and sodium channels (C); Rho kinases (D); protein kinase C (E); calcium activated potassium channels (F); voltage-gated potassium channels (G); inwardly rectifying potassium channels (H) and two-pore domain potassium channels (I). (J-M) Time-lapse recording of calcium concentrations as indicated by fluo-4 AM in the VSMC layer of the aorta after exposure to 10uM Phe (J, K) or 10uM calcium ionophore A23187 (L, M) at 2 weeks after Tam, 6 vessels per group. Continuous recording of fluorescent images with excitation at 495nm and emission at 505-525nm was performed every 15 seconds. The F_1/F_0 ratio refers to the fluorescent intensity at a specific time/fluorescence intensity in the beginning time 0. All quantification data are represented as the mean ± SEM. The *P* values are calculated by two-way ANOVA followed by Tukey's multiple comparisons or Student's *t*-tests. Statistics for time-response relationships were performed with calculation of area under curve followed by *t*-test analysis. (A-M) All data were not statistically significant.

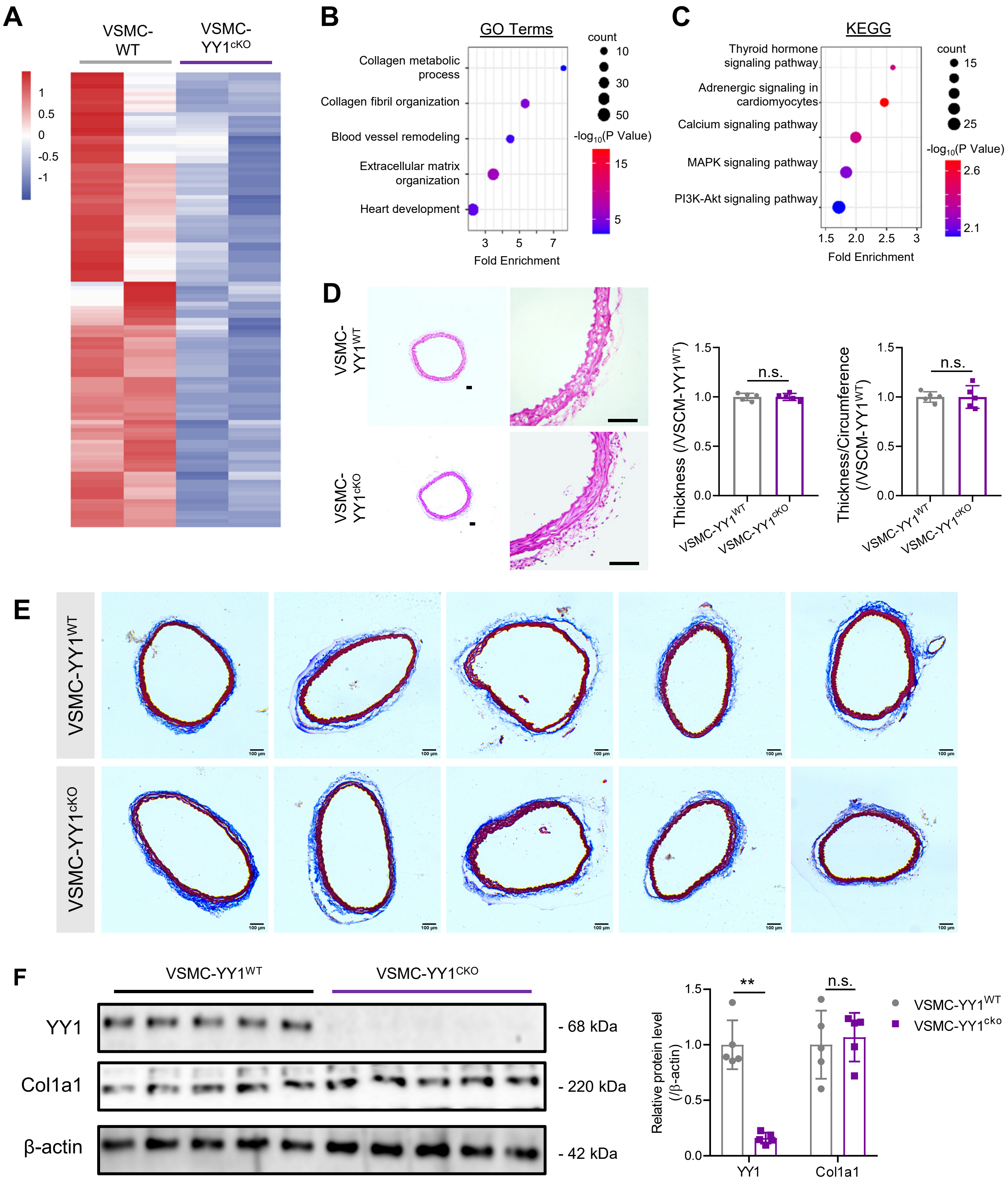

Figure S4 Characterization of VSMC-specific YY1-deficient mice. (A-C) Bulk RNA-seq data from the aortic media of VSMC-YY1^{cKO} and VSMC-YY1^{WT} at day 7 after tamoxifen (Tam) administration. (A) Heatmap of 115 significantly downregulated genes identified by GO enrichment analysis (B) and KEGG pathway analysis (C). (D) H&E staining of aortas at 4 weeks after Tam. Quantification of aortic thickness and thickness-to-circumference ratio was performed, 5 vessels per group. Scale bars: 100 um. (E) Masson's trichrome staining for collagen fibers in aortas from VSMC-YY1^{WT} and VSMC-YY1^{cKO} mice at 2 weeks after Tam, 5 vessels per group. Scale bars: 100 um. (F) Western blot analysis and quantification of protein expression in aortic VSMCs from VSMC-YY1^{WT} and VSMC-YY1^{cKO} mice at 2 weeks after Tam, 5 vessels per group. All quantification data are presented as mean ± SEM. *P* values were calculated by *t*-test and two-way ANOVA followed by Tukey's multiple comparisons. **p<0.01.

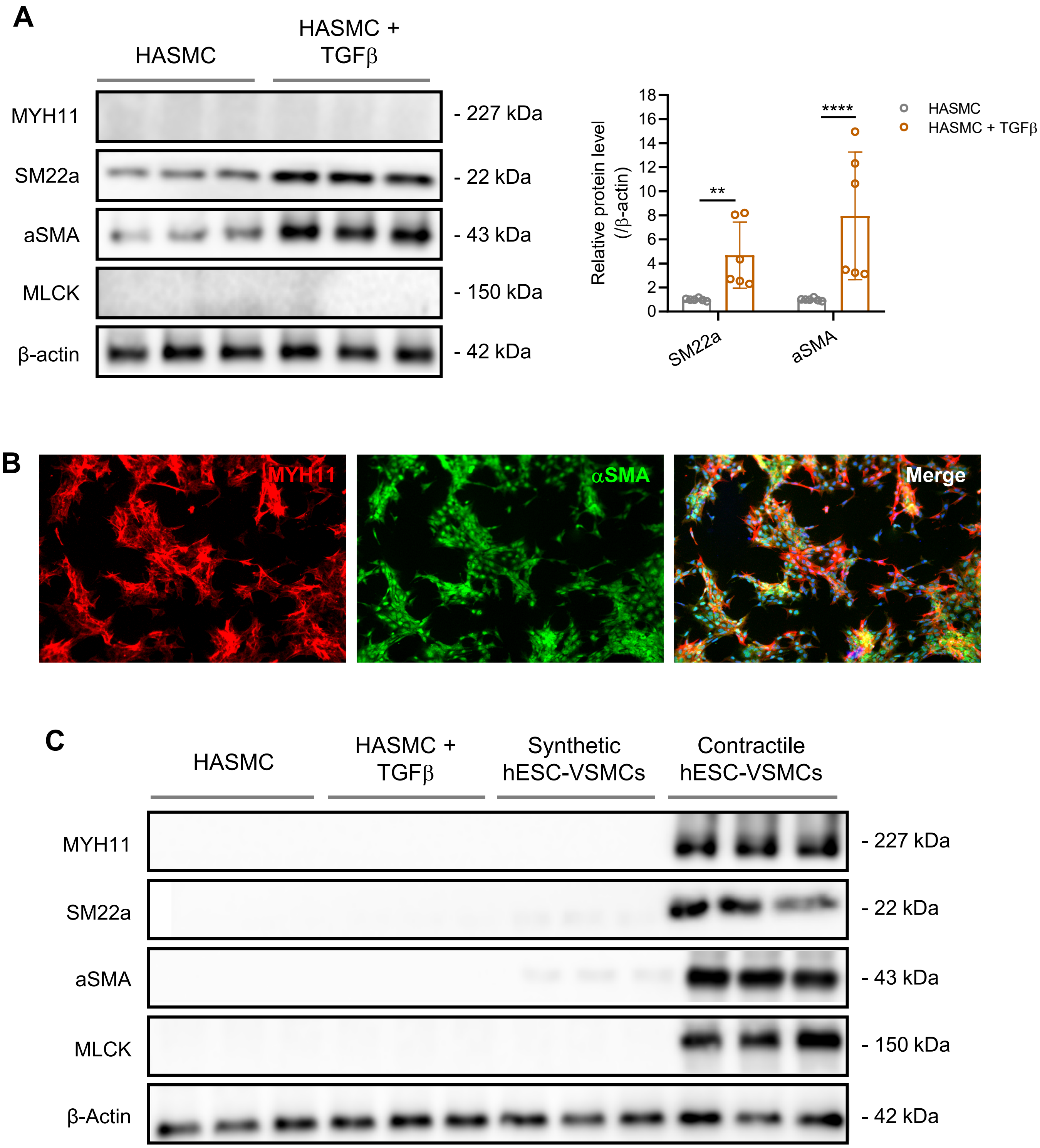

Figure S5 hESC-VSMCs are superior to the commonly used HASMC cell line for modelling quiescent, contractile VSMCs. (A) Western blot and quantification of protein expression in HASMCs. HASMCs were treated with solvent or TGFb (10 ng/mL) for 24 hours before harvest, 6 vessels per group. All quantification data are represented as mean ± SEM. *P* values were calculated by two-way ANOVA followed by Tukey's multiple comparisons. **p<0.01, ****p<0.0001. (B) Immunocytochemistry for MYH11 (red), aSMA (green) and Hoechst (blue) in contractile hESC-VSMCs at day 9 of hESC differentiation. (C) Western blot and quantification of protein expression in HASMCs and hESC-VSMCs. HASMCs were treated with solvent or TGFb (10 ng/mL) for 24 hours before harvest; and contractile hESC-VSMCs without or with PDGF-BB (10 ng/mL) for 3 more days for induction of synthetic phenotype.

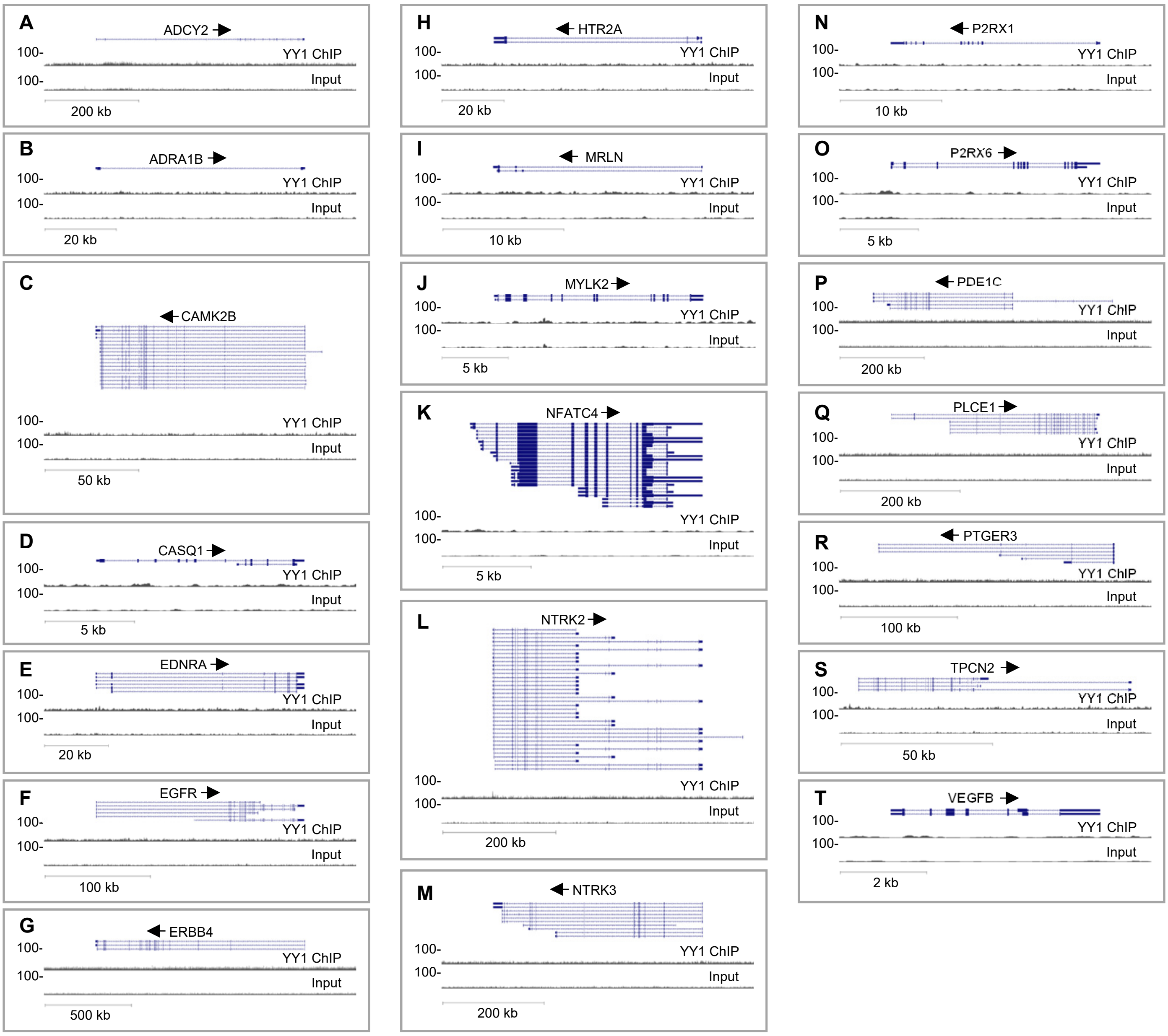

Figure S6 YY1 does not directly regulate expression of genes of the calcium signalling pathway *in vivo*. (A-T) Genome snapshot from YY1 ChIP-seq analysis using hESC-VSMCs targeting the differentially expressed genes of the calcium signalling pathway identified by bulk RNA-seq (Fig S4C, Table S2).

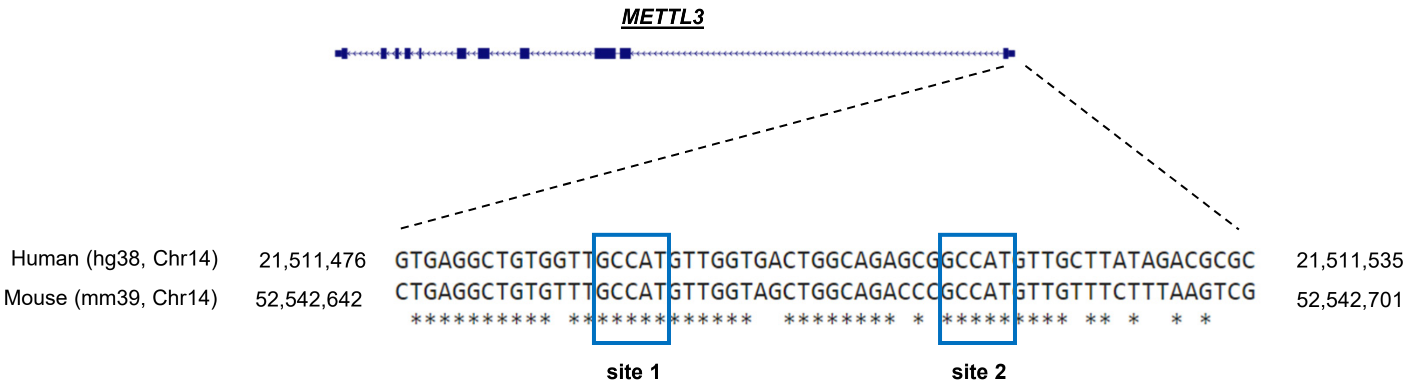

Figure S7 The canonical binding sites of YY1 in the putative promoter regions of *Mettl3* are conserved across human and mouse. A portion of alignment of orthologous regions between *Mettl3* putative promoter regions in human (hg38) and mouse (mm39) genomes was determined by Clustal Omega. Asterisks (*) denote identical nucleotides between the two DNA sequences. Blue boxes indicate the core motif sequence of YY1.

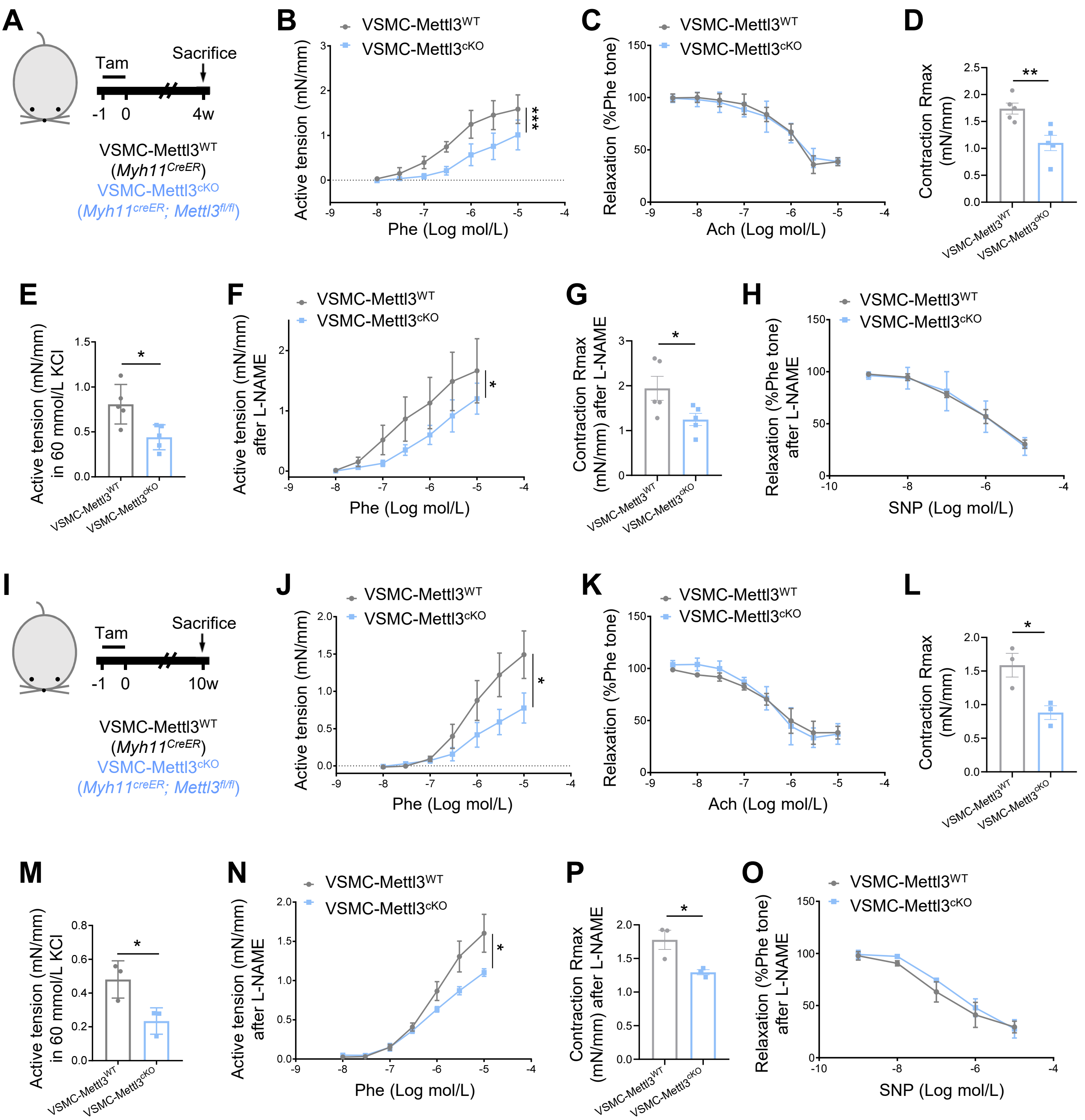

Figure S8 Loss of Mettl3 in VSMCs impairs mesenteric vessel contraction *in vivo*. (A) A schematic showing the experimental design and the generation of Myh11-CreER (VSMC-Mettl3^{WT}) and Myh11-CreER;Mettl3$^{fl/fl}$ (VSMC-Mettl3^{cKO}) mice. (B) Concentration-dependent vasoconstriction responses of mouse mesenteric vessels to phenylephrine (Phe), and (C) concentration-dependent relaxation responses to acetylcholine (Ach) were measured in VSMC-Mettl3^{cKO} and VSMC-Mettl3^{WT} mice 4 weeks after tamoxifen (Tam) treatment, 5 vessels per group. (D) The maximum responses (Rmax) in vasoconstrictions and (E) 60 mM KCl-mediated contractile responses were examined, 5 vessels per group. (F) Endothelium-independent vasoconstrictions, (G) Rmax in vasoconstrictions, and (H) relaxations were evaluated in the presence of the nitric oxide synthase inhibitor L-NAME, 5 vessels per group. (I) A schematic showing the experimental design. (J) Concentration-dependent vasoconstriction responses of mouse mesenteric vessels to phenylephrine (Phe), and (K) concentration-dependent relaxation responses to acetylcholine (Ach) were measured in VSMC-Mettl3^{cKO} and VSMC-Mettl3^{WT} mice 10 weeks after Tam treatment, 5 mesenteric vessels per group. (L) Rmax in vasoconstrctions and (M) 60 mM KCl-mediated contractile responses were examined, 3 vessels per group. (N) Endothelium-independent vasoconstrictions, (P) Rmax in vasoconstrictions, and (O) relaxations were evaluated in L-NAME, 3 vessels per group. All quantification data are represented as the mean ± SEM. *P* values were calculated by Student's *t*-tests. Statistics for concentration-response relationships were performed by calculating the area under curve followed by *t*-test analysis. *p<0.05, **p<0.001, ***p<0.001

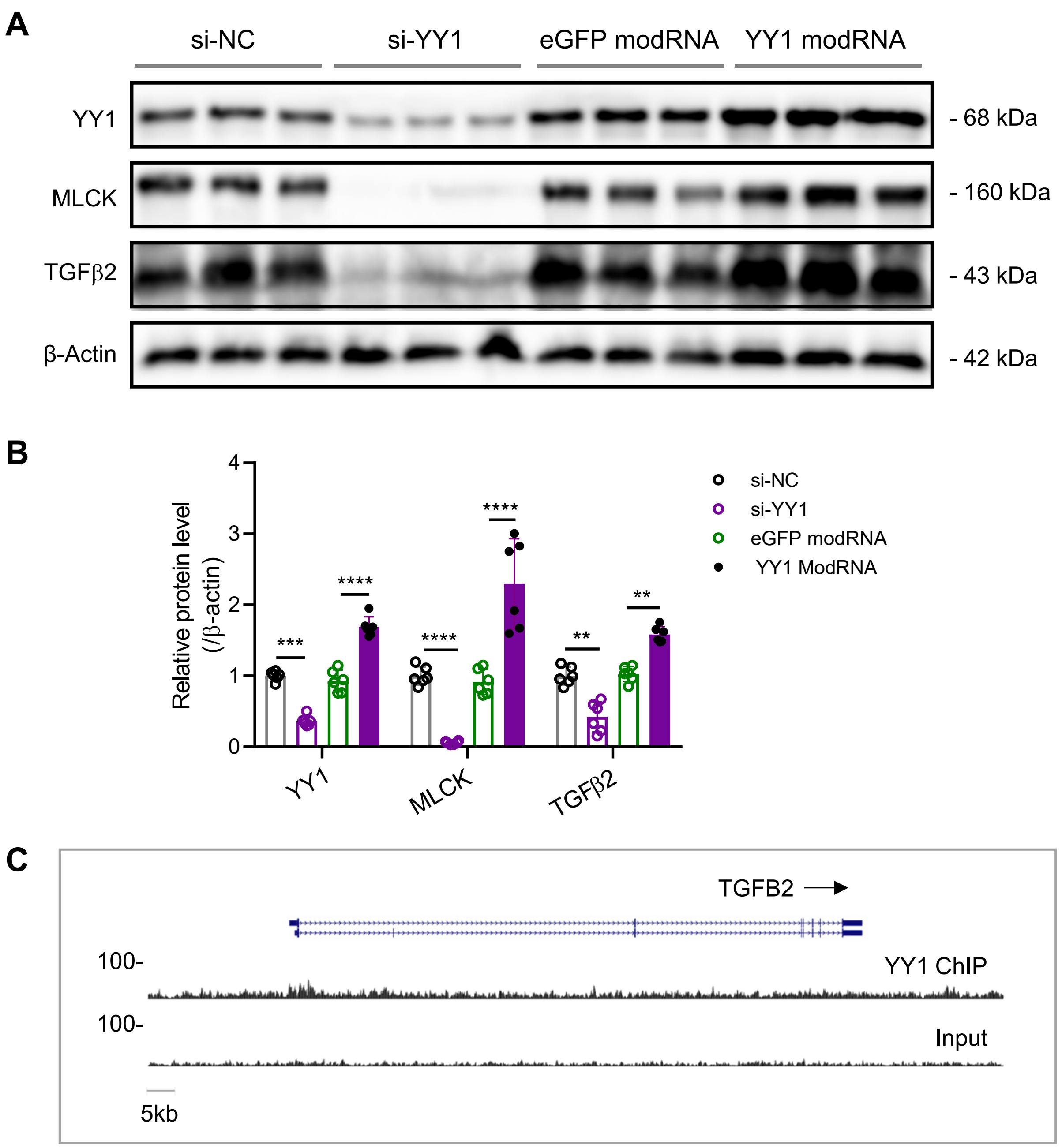

Figure S9 YY1 regulates MLCK and TGFb2 expression in hESC-VSMCs. (A) Western blot and (B) quantification of protein expression in hESC-VSMCs. Cells were treated with control or *YY1* siRNA for 48 hours; or with eGFP or *YY1* modified mRNA (modRNA, 1 mg/mL) for 48 hours before harvest, 6 samples per group. All quantification data are represented as the mean ± SEM. The *P* values are calculated by two-way ANOVA followed by Tukey's multiple comparisons. **p<0.01, ***p<0.001, ****p<0.0001.(C) Genome snapshot from YY1 ChIP-seq analysis targeting *TGFB2* gene in hESC-VSMCs.

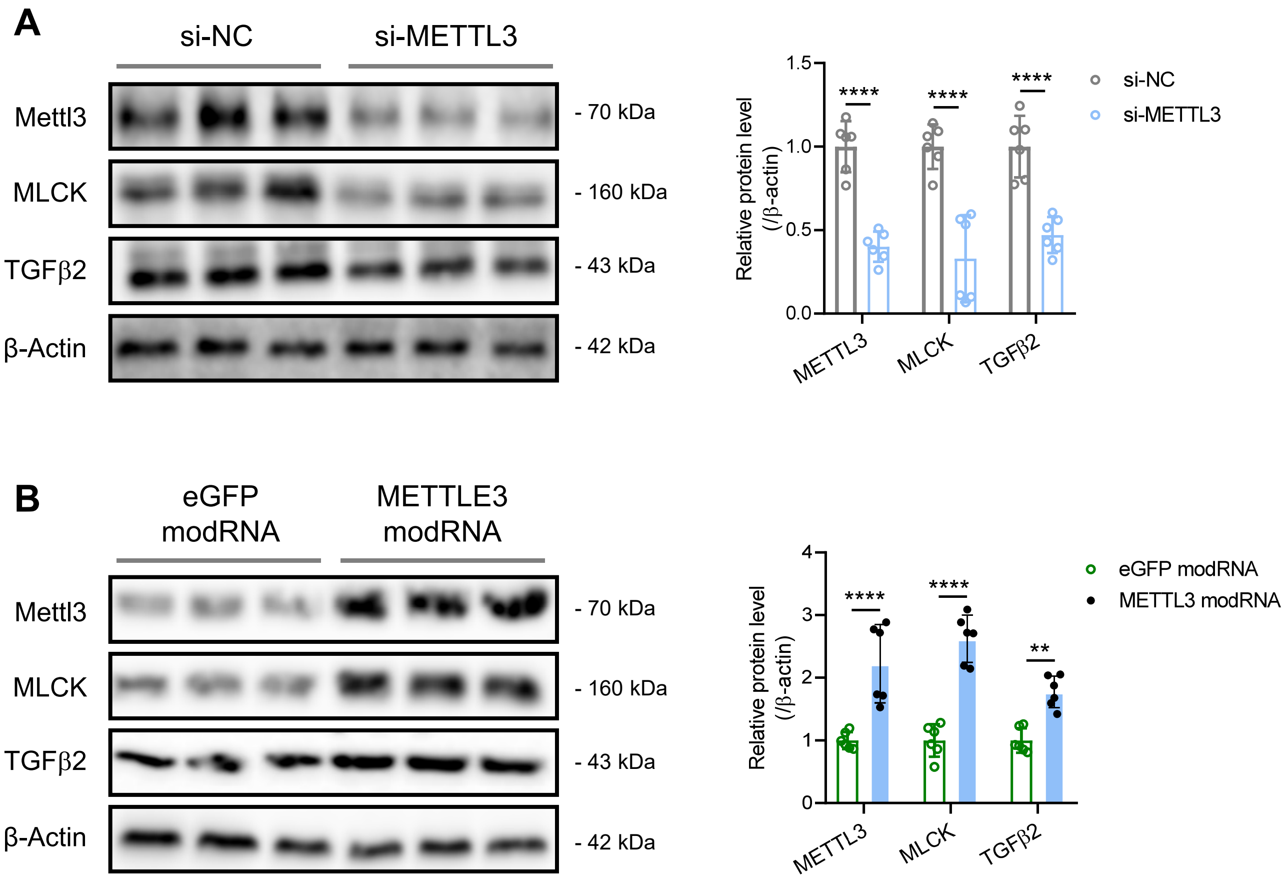

Figure S10 METTL3 regulates MLCK and TGFb2 expression in hESC-VSMCs. (A, B) Western blot and quantification of protein expression in hESC-VSMCs treated with control or *METTL3* siRNA for 48 hours (A); or with *eGFP* or *METTL3* modified mRNA (modRNA, 1 mg/mL) for 48 hours (B), 6 vessels per group. All quantification data are represented as mean ± SEM. *P* values were calculated by two-way ANOVA followed by Tukey's multiple comparisons. **p<0.01, ****p<0.0001.

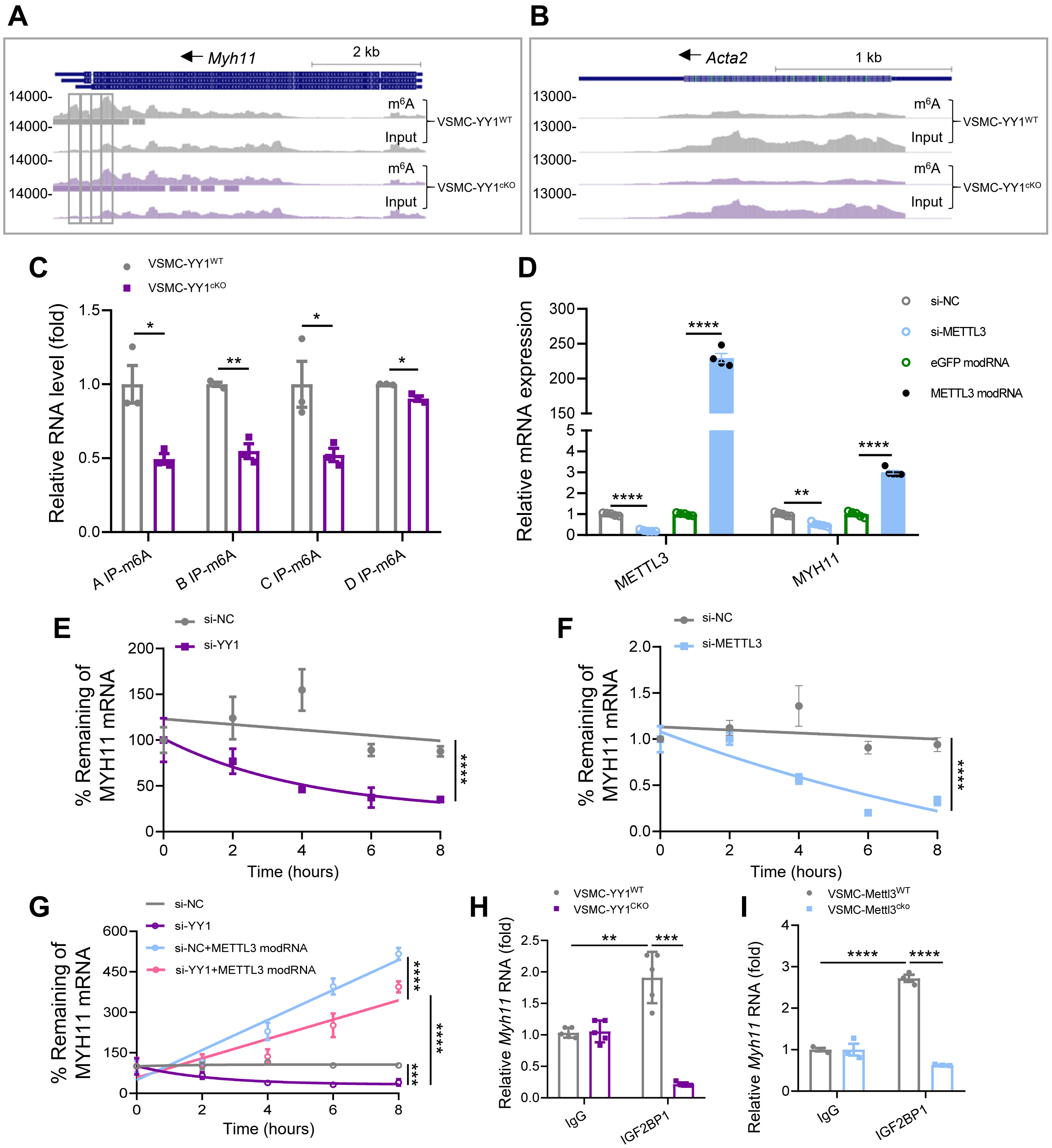

Figure S11 The YY1/Mettl3 axis controls transcription of *Myh11* in VSMCs. (A, B) Genome snapshot from YY1 ChIP-seq analysis using hESC-VSMCs targeting Myh11 and Acta2. (C) m^{6}A RIP followed by quantitative RT-qPCR, 3 vessels per group. RNA was extracted from aortic media of VSMC-YY1^{cKO} and VSMC-YY1^{WT} mice at day 7 after Tam, and then precipitated with an anti-m^{6}A antibody. The RNA fragments were evaluated for enrichment by RT-qPCR using specific primers targeting the m^{6}A peak regions of *Myh11* mRNA as indicated in boxes of (A). (D) RT-qPCR targeting *MYH11* and *METTL3* using hESC-VSMCs in different treatments, 4-5 vessels per group. Cells were treated with control or *METTL3* siRNA for 48 hours; or with eGFP or *METTL3* modified mRNA (modRNA, 1 mg/mL) for 48 hours before harvest. (E, F) Decay curves of *MYH11* mRNAs were quantified in hESC-VSMCs after treated with *YY1* siRNA (E) and *METTL3* siRNA (F) for 24 hours, 6 samples per group. (G) Decay curves of *MYH11* mRNAs were quantified in hESC-VSMCs after treated with *YY1* siRNA and *METTL3* modified mRNA (modRNA, 1 mg/mL) for 48 hours, 6 vessels per group. (H, I) IGF2BP1 RIP followed by RT-qPCR, 6 vessels per group. RNA was extracted from aortic media of VSMC-YY1^{cKO} and VSMC-YY1^{WT} (H), and VSMC-Mettl3^{cKO} and VSMC-Mettl3^{WT} (I) mice at day 7 after Tam, and then precipitated with an anti-IGF2BP1 antibody. All quantification data are represented as mean ± SEM. *P* values were calculated by two-way ANOVA followed by Tukey's multiple comparisons. Statistics for time-response relationships were performed by calculating the area under curve followed by *t*-tests or by two-way ANOVA with Tukey's multiple comparisons. *p<0.05, **p<0.01, ***p<0.001, ****p<0.0001.

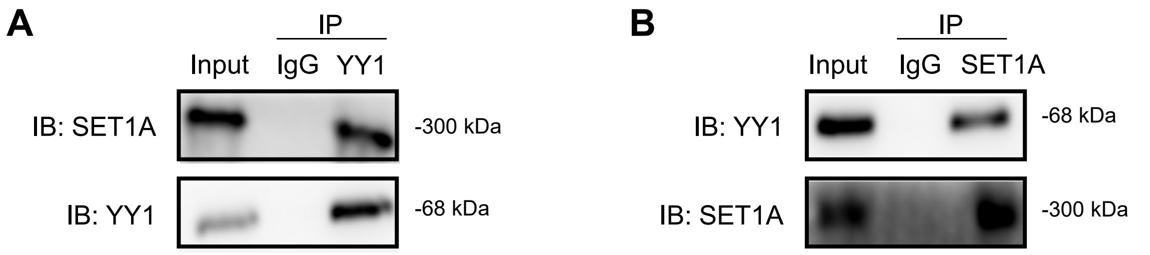

Figure S12 YY1 interacts with SET1A in the absence of regulatory DNA. Reciprocal co-immunoprecipitation was performed to demonstrate the endogenous interaction between YY1 and SET1A in the aortic media. (A) Protein was extracted, treated with Dnase I for 15 minutes to remove regulatory DNA, and precipitated with an anti-YY1 antibody. The interaction with SET1A was detected. (B) Protein was extracted, treated with Dnase I () to remove regulatory DNA, and precipitated using an anti-SET1A antibody, confirming the interaction with YY1. IgG was used as a control to rule out nonspecific binding.

Supplementary Tables

Table S1 The sensitivity/potency of different drugs is needed for half-maximal response of the aortas after YY1 knockout in VSMCs as demonstrated in Figure 1.

Figure 1	Drugs	LogEC50 of VSMC-YY1^{WT} (mol/L)	LogEC50 of VSMC-YY1^{cKO} (mol/L)	P value
B	Phe	-7.279±0.159	-7.193±0.096	ns
C	Ach	-6.424±0.193	-6.594±0.161	ns
F	Phe	-7.044±0.043	-7.078±0.036	ns
G	SNP	-7.257±0.132	-7.389±0.100	ns
H	Phe	-7.013±0.100	-6.940±0.018	ns
K	SNP	-7.193±0.173	-7.106±0.233	ns
L	$CaCl_2$	-2.983±0.033	-2.948±0.049	ns

Table S2 The sensitivity/potency of different drugs is needed for half-maximal response of the mesenteric vessels after YY1 knockout in VSMCs as demonstrated in Figure S2.

Figure S2	Drugs	LogEC50 of VSMC-YY1WT (mol/L)	LogEC50 of VSMC-YY1cKO (mol/L)	P value
B	Phe	-6.014±0.096	-5.973±0.113	ns
C	Ach	-6.406±0.172	-6.581±0.165	ns
G	Phe	-6.139±0.060	-6.356±0.097	ns
H	SNP	-6.696±0.117	-6.644±0.097	ns
J	Phe	-5.716±0.063	-5.777±0.100	ns
K	Ach	-6.759±0.094	-6.757±0.049	ns
O	Phe	-5.886±0.164	-5.565±0.066	ns
P	SNP	-7.401±0.084	-7.044±0.184	ns

Table S3 GO enrichment analyses from bulk RNA-seq data showing the top biological pathways significantly downregulated in aortic media of VSMC-YY1^{cKO} compared to that of VSMC-YY1^{WT} at 7 days after tamoxifen administration.

Pathway	P-value	Genes
GO:0030198~extracellular matrix organization	2.47E-07	COL15A1, OLFML2B, TNXB, NDNF, ADAMTS12, OLFML2A, COL19A1, MMP23, ADAMTSL1, ADAMTS5, ADAMTS15, ADAMTS2, ADAMTS14, ADAMTSL4, ABI3BP, TGFB2, FKRP, COL1A1, COL3A1, MMP14, COL1A2, CRISPLD2, COL6A6, RECK, MATN2
GO:0030199~collagen fibril organization	1.19E-05	COL1A1, EXT1, TGFB2, ADAMTS14, ADAMTS2, COL3A1, TNXB, COL1A2, LOX, COL14A1, ADAMTS12, LOXL2
GO:0007507~heart development	1.71E-04	POPDC3, PRKDC, DNAH5, ADM, PKD2, ZFP36L1, MED12, EDNRA, ERBB4, CASP3, GJA6, PDLIM4, CC2D2A, SOX4, TGFB2, FKRP, OSR1, NTRK3, PDE2A, VEGFB, AGTR1A, NFATC4, ADAM19, COL3A1, BMP2, LOX, NACA
GO:0001974~blood vessel remodeling	8.16E-04	EXT1, TGFB2, EDNRA, ANGPT2, NPR3, HOXA3, RSPO3, NDP, ADRA1B
GO:0032963~collagen metabolic process	0.003559	TNXB, COL1A2, P3H2, HIF1A, FOSL2

Table S4 KEGG analyses from bulk RNA-seq data showing the top gene pathways significantly downregulated in aortic media of VSMC-YY1^{cKO} compared to that of VSMC-YY1^{WT} at 7 days after tamoxifen administration.

Pathway	P-value	Genes
mmu04261:Adrenergic signaling in cardiomyocytes	0.002042	CAMK2B, POPDC3, CACNA2D3, ADCY2, ATP1B2, ADRA1B, AGTR1A, CACNB2, ACTC1, PPP2R1B, PPP2R3D, PPP1R1A, CREB3L1, PPP2R2D, ATF4, MYH7
mmu04919:Thyroid hormone signaling pathway	0.004046	NCOA1, CREBBP, THRB, SLC16A10, ATP1B2, MED16, HIF1A, MED12L, MED12, PLCE1, SLC16A2, MYH7, WNT4
mmu04020:Calcium signaling pathway	0.004208	CAMK2B, NTRK2, MYLK2, MRLN, PDE1C, NTRK3, PTGER3, VEGFB, ADCY2, HTR2A, ADRA1B, EGFR, AGTR1A, TPCN2, NFATC4, P2RX6, EDNRA, ERBB4, P2RX1, PLCE1, CASQ1
mmu04010:MAPK signaling pathway	0.006948	NTRK2, ANGPT4, TGFB2, ANGPT2, GADD45B, HSPA1L, PLA2G4B, VEGFB, CACNA2D3, RASGRP1, EGFR, CACNB2, NR4A1, DUSP10, ARTN, ERBB4, CASP3, NTF3, MAPT, MAP4K3, HSPA1B, ATF4, HSPA1A
mmu04151:PI3K-Akt signaling pathway	0.008985	TNXB, LAMA2, TNC, EGFR, GHR, GNG10, PPP2R1B, PPP2R3D, ERBB4, CREB3L1, NTF3, ITGB8, ANGPT4, NTRK2, ANGPT2, VEGFB, COL1A1, NR4A1, CDK6, COL1A2, ARTN, COL6A2, PPP2R2D, IL2RB, COL6A6, ATF4

Table S5 The sensitivity/potency of different drugs is needed for half-maximal response of the aortas after Mettl3 knockout in VSMCs as demonstrated in Figure 3.

Figure 3	Drugs	LogEC50 of VSMC-Mettl3WT (mol/L)	LogEC50 of VSMC-Mettl3cKO (mol/L)	P value
B	Phe	-6.964±0.087	-6.893±0.108	ns
C	Ach	-6.181±0.154	-6.410±0.032	ns
F	Phe	-7.058±0.145	-7.100±0.046	ns
G	SNP	-7.141±0.102	-6.952±0.049	ns

Table S6 The sensitivity/potency of different drugs is needed for half-maximal response of the aortas after Mettl3 knockout in VSMCs as demonstrated in Figure S8.

Figure S8	Drugs	LogEC50 of VSMC-Mettl3WT (mol/L)	LogEC50 of VSMC-Mettl3cKO (mol/L)	P value
B	Phe	-6.319±0.052	-6.173±0.064	ns
C	Ach	-6.141±0.051	-6.217±0.129	ns
G	Phe	-6.387±0.240	-5.863±0.140	ns
H	SNP	-6.381±0.083	-6.345±0.234	ns
J	Phe	-6.135±0.068	-6.040±0.044	ns
K	Ach	-6.498±0.080	-6.517±0.066	ns
O	Phe	-6.077±0.030	-6.123±0.011	ns
P	SNP	-6.937±0.149	-6.398±0.232	ns

Table S7 GO enrichment analyses from bulk RNA-seq showing the top biological pathways significantly and commonly downregulated in aortic media of VSMC-Mettl3^{cKO} and VSMC-YY1^{cKO} compared to their respective controls at day 7 after tamoxifen treatment.

Pathway	P-value	Genes
GO:0030199~collagen fibril organization	4.15E-04	COL1A1, TGFB2, ADAMTS2, TNXB, LOX
GO:0030198~extracellular matrix organization	9.30E-04	COL1A1, TGFB2, ADAMTS2, MMP14, TNXB, ADAMTSL4, CRISPLD2
GO:0043066~negative regulation of apoptotic process	0.024827	TGFB2, DAPK1, NNT, IL2RB, ALB, TWIST2, VEGFB, HSPA1B, AQP1, SNCA
GO:0006468~protein phosphorylation	0.043101	CAMK2B, MYLK2, NTRK2, TGFB2, PKDCC, DAPK1, RARA, HUNK, HIPK2
GO:0060395~SMAD protein signal transduction	0.095041	TGFB2, GDF15, HIPK2

Table S8 KEGG analyses from bulk RNA-seq showing the top gene pathways significantly and commonly downregulated in aortic media of VSMC-Mettl3^{cKO} and VSMC-YY1^{cKO} compared to their respective controls at day 7 after tamoxifen treatment.

Pathway	P-value	Genes
mmu04151:PI3K-Akt signaling pathway	0.0052	GHR, COL1A1, NTRK2, TNXB, PPP2R3D, COL6A2, CREB3L1, IL2RB, VEGFB
mmu04261:Adrenergic signaling in cardiomyocytes	0.028097	CAMK2B, POPDC3, PPP2R3D, CREB3L1, MYH7
mmu04310:Wnt signaling pathway	0.040302	CAMK2B, TLE3, CREBBP, FZD8, WNT4
mmu04919:Thyroid hormone signaling pathway	0.058964	CREBBP, MED16, WNT4, MYH7
mmu04010:MAPK signaling pathway	0.071392	NTRK2, TGFB2, HSPA1L, VEGFB, HSPA1B, HSPA1A

Table S9 The sensitivity/potency of different drugs is needed for half-maximal response of the aortas after Mettl3 overexpression in YY1 knockout VSMCs as demonstrated in Figure 5.

Figure 5	Drugs	LogEC50 of VSMC-YY1cKO + AAV-ZsGreen (mol/L)	LogEC50 of VSMC-YY1cKO + AAV-Mettl3 (mol/L)	P value
G	Phe	-6.832±0.162	-6.930±0.097	ns
H	SNP	-7.799±0.169	-7.639±0.149	ns

Table S10 siRNA sequences used in this study.

Human genes	siRNA sequence
YY1-1345	5' GAAGAUGAUGCUCCAAGAATT 3'
YY1-1665	5' UCAGUCAACUAACCUGAAATT 3'
METTL3-1400	5' GCUCAACAUACCCGUACUATT 3'
METTL3-1604	5' GGUUGGUGUCAAAGGAAAUTT 3'
SET1A-2081	5'GUCCCUUCCUCUUGGUUAUTT 3'
SET1A- 4477	5'GCAGUGAGUUUGAACAGAUTT 3'
SET1A -5209	5'CCCAGAAGAAGAUCGUGAUTT 3'

Table S11 RT-qPCR primers used in this study.

Genes	Forward	Reverse
Human genes		
YY1	GGAGGAATACCTGGCATTGACC	CCCTGAACATCTTTGTGCAGCC
METTL3	CTATCTCCTGGCACTCGCAAGA	GCTTGAACCGTGCAACCACATC
MYLK2	GACAAGGCACCTAAAGGTCCC	TTGGCTGCTAGTTGAGGGTTG
TGFB2	AAGAAGCGTGCTTTGGATGCGG	ATGCTCCAGCACAGAAGTTGGC
MYH11	GTCCAGGAGATGAGGCAGAAAC	GTCTGCGTTCTCTTTCTCCAGC
β-actin	CTTCCAGCCTTCCTTCCTGG	CTGTGTTGGCGTACAGGTCT
Mouse genes		
Yy1	ACCTGGCATTGACCTCTCAGAC	CTCATAGCAGAGTTATCCCTGAAC
Mettl3	CAGTGCTACAGGATGACGGCTT	CCGTCCTAATGATGCGCTGCAG
Mylk2	GCGAGACAACAGACCTCGTC	GGTGTCCCCTTGCACCTTAG
Tgfb2	TGGGTACCTTGATGCCATCC	GATTTAAGGATCTGATACAG

Myh11	GCAACTACAGGCTGAGAGGAAG	TCAGCCGTGACCTTCTCTAGCT
β-actin	CATTGCTGACAGGATGCAGAAGG	TGCTGGAAGGTGGACAGTGAGG

Table S12 ChIP-qPCR primers used in this study.

Human genes	Forward	Reverse
METTL3	TCCAGGATATAGCCAATTCT	GACTCCTCTCCGAATATTCA

Table S13 m^{6}A-qPCR primers used in this study.

Mouse genes	Forward	Reverse
Tgfb2-A	CGTGTTTGCTGGGGCTTTGA	AAATAAGCCAGGGGGAAGGA
Tgfb2-B	CAAACAGAACACAAGCTTGC	ACCATGATGTTTGTGACAGG
Tgfb2-C	CGTCATCGTTGTCATTATAT	CAGCTTTCCAATATGATTGT
Tgfb2-D	TTCGATCTTGGGCGTATTTC	GTTCAGACACTCAACACACC
Mylk2-A	CAATCTGACAACCTGATACA	TTCCCAGCTCAACTGCTCCG
Mylk2-B	TCCTGTCTGCCAACTGGTAC	TCAGCACTCATTCTGGCACT
Mylk2-C	TGGCGGGGAGAGACCTGGGA	GTTGTGACTGCTCCTGGGAT
Mylk2-D	CTCTGTGCCCATCCCAGGAG	AGGCACAAGCTGAGAGCAGG
Myh11-A	CTGGTGACTGTGGCTCCTGAC	CAGCAGAGCCCAGCAGACTG
Myh11-B	CCTGACATGGTGTCCAATCC	GAAACTTCGCAGTGAGCCAC
Myh11-C	CCCTCTGAGTTTGCTCTTGA	GCAGAGAAAGGAAACACCAA
Myh11-D	CTGCTCCTTGTACTGCTCTGC	CAAGTCCACTGTTGCGGC

Table S14 RIP-qPCR primers used in this study.

Mouse genes	Forward	Reverse
Tgfb2	CGCACGCACGCGCGCACACG	TAACTGAGAAGTTCAAAGAC
Mylk2	AGCCATCGGGCAGACCCCAA	TCACACACACGTGTGTGCAC
Myh11	GCCCTTCTGGAAGGAACAAATG	CTCAAGAGCAAACTCAGAGGG

Supplementary Methods

Human embryonic stem cell and hESC-VSMC cultures

The human embryonic stem cell line (hESC) H9 (Wicell, Madison, WI) was cultured in mTeSR™ Plus medium (Stemcell) per manufacturer's instructions. Cells were refreshed daily to maintain the undifferentiated state and passaged using Accutase (Life Technologies). hESCs were differentiated into hESC-VSMCs with a protocol modified from a previous report[30]. Briefly, hESCs were passaged onto a growth factor reduced matrigel coated plate and cultured overnight in mTeSR + ROCK inhibitor Y-27632 (10μM) with a density ranging from 37.000-47.000 cells per cm^2. On the following day, mTeSR was refreshed with N2B27 medium containing 1:1 DMEM/F12 and Neurobasal medium supplemented with Glutamax, β-Mercaptoethanol, N2 and B27 supplements (all Life Technologies). Cells were cultured in N2B27 medium supplemented with CHIR-99021 (6-8 μM) and hBMP4 (25 ng/mL) for 3 days. On day four, the medium was replaced with N2B27 medium supplemented with PDGF-BB (10 ng/mL, Peprotech) and Activin A (2 ng/mL, Peprotech) every day for 2 days. On day six, cells were seeded on collagen-coated wells at a concentration of 3.7×10^4 cells/cm^2 and cultured in N2B27 medium supplemented with Activin A (2 ng/mL) and Heparin (2 μg/mL, Sigma) for 3 days to induce the contractile phenotype. For the induction of contractile to synthetic phenotypic switching, contractile hESC-VSMCs were refreshed with N2B27 medium supplemented with PDGF-BB (10 ng/mL, Peprotech) and cultured for 3 more days.

HASMC cultures

The primary HASMC cell line (Human primary aortic smooth muscle cells, ATCC, PCS-100-012) was cultured in vascular cell basal medium (ATCC, PCS-100-030) supplemented with Vascular Smooth Muscle Growth Kit components (ATCC, PCS-100-042) at 37°C with 5% CO_2. To induce a contractile phenotype, HASMCs were supplemented with TGFβ (10 ng/mL) for 24 hours.

Lentiviral transduction

For stable knockdown of *YY1* and *METTL3* in hESC-VSMCs, scrambled control shRNA, *YY1* (Vigene Biosciences), and *METTL3* (Hanheng Biotechnology Co.,Ltd., Shanghai, China) shRNAs were purchased from the companies. The sequences of YY1 and METTL3 siRNAs were listed in Table S10. In some experiments, YY1 and METTL3 were overexpressed in

hESC-VSMCs. Lentiviral overexpression vectors were constructed with the help from Hanheng Biotechnology Co. Ltd., Shanghai, China. hESC-VSMCs were seeded on 6-well plates overnight, and supplemented with the virus stock solution with polybrene (MOI = 30). At 12 hours after infection, the medium was refreshed with complete culture medium and cells were further cultured at 37°C for 48 hours unless otherwise specified.

Generation of modified mRNA

In some experiments, *eGFP*, *YY1* and *METTL3* modified mRNAs (modRNA) were synthesized as previously described[33]. Briefly, open reading frames were amplified by PCR from plasmids encoding eGFP, human YY1 or Mettl3. RNA was synthesized with the MEGAscript T7 kit (Thermofisher, Cat. No. AMB13345), and a custom ribonucleoside blend was used comprising 3´-O-Me-m7G(5')ppp(5')G cap analog (New England Biolabs, Cat. No. S1411L), adenosine triphosphate and guanosine triphosphate (TriLink Biotechnologies, Cat. No. N-1014-1), 5-methylcytidine triphosphate and pseudouridine triphosphate (TriLink Biotechnologies, Cat. No. N-1091-1). For transfection, modRNA and RNAiMAX transfection agent were each dissolved separately in Opti-MEM (Invitrogen), combined, and then incubated for 15 min at room temperature to generate a transfection mixture. *In vitro* transfection was performed by adding the transfection mixture (1 µg modRNA) to cells plated in each of a six-well plate with DMEM supplemented with 2% FBS and 200 ng/ml B18R (eBioscience) or in Pluriton Reprogramming Medium (Stemgent).

Bulk RNA-sequencing and library preparation

RNA-seq library preparation was performed as previously described[12]. Total RNA was isolated and analyzed on the Agilent Tape station for RNA integrity numbers (RINs) before library preparation. RNA-seq libraries were prepared using the NEBNext® Ultra™ II RNA Library Prep Kit (NEB, Cat. No. E7775) according to the manufacturer's protocol. mRNA was isolated using poly-T oligos conjugated to magnetic beads and then fragmented and reverse-transcribed to first strand cDNA. During the synthesis of the second strand, deoxyuridine triphosphates (dUTPs) were included and consequently were not amplified. Double strand cDNA was purified by SPRIselect Beasds (Beckman Coulter). cDNA underwent end prep reaction, ligation with indexed adaptors, purification, and PCR amplification. PCR reaction was purified by SPRIselect Beasds (Beckman Coulter). RNA libraries were quantified, pooled, and

sequenced at paired-end 150 bp on the Illumina NovaSeq 6000 platform with a total of 65 million reads per library.

Quantitative real time-PCR (RT-qPCR)

Total RNA from tissues and cells was extracted using TRIzol reagent (Vazyme, China) and cDNA was generated with iScript™ cDNA Synthesis Kit (Bio-Rad, USA) according to the manufacturers' instructions. Briefly, the tissues were minced and grounded with a grinding rod for 1 min by adding liquid nitrogen and TRIzol reagent. RT-qPCR was performed using CFX Connect Read-Time PCR Detection system (Bio-Rad) via SYBR Green (Bio-Rad) according to the manufacturer's instructions. Gene expression levels were normalized to that of β-actin. The relative gene expression level of each sample was compared with an internal control. Primers used are listed in Table S11.

ChIP-seq and ChIP-qPCR

ChIP-seq library preparation was performed as previously described[12]. ChIP assays were performed in hESC-VSMCs or mouse aortic media with a SimpleChIP Enzymatic Chromatin IP Kit (#S9003, CST, USA) according to the manufacturer' s instructions. The mouse aortic tissues were minced and grounded with a grinding rod for 1 min by adding liquid nitrogen and PBS. The aortic tissue or hESC-VSMCs were cross-linked with 1% formaldehyde (Sigma) at room temperature for 10 min. The reaction was quenched by an addition of 0.125 M glycine for 5 min, and the cross-linked chromatin was fragmented by 0.5μl Micrococcal Nuclease (CST) for 20 min at 37 °C with sonication (Shanghai Lichen Bangxi Technology Co., Ltd.) for 3 times (20 s /time, peak power 250W, power ratio 20%). The sheared chromatin was then taken and 10% of which served as "Input DNA" and negative control. The rest was incubated with 10 μl rabbit anti-human YY1 (Active Motif, Cat. No. 61779), rabbit anti-human H3K4me3 (abcam, ab8580), rabbit anti-human H3K27ac (abcam, ab4729), rabbit anti-human H3K27me3 (Millipore, #07-449), or mouse anti-human IgG antibody (1:1000 dilution, Millipore, #12-371), with rotation at 4°C overnight. The reaction was subsequently pulled with ChIP-Grade protein G Magnetic Beads (CST, #9006). The protein G magnetic beads were then washed with low salt for 3 times (5 min/ time), and high salt for 1 time (5 min/ time). The ChIP elution buffer (CST, #7009) was added to the input sample and each ChIP sample for 30 min at 65 °C with gentle vortex, and then added 6 μl 5M Nacl and 2 μl Proteinase K (CST, #10012), and incubated 2 h at 65 °C. For ChIP-seq, the immunoprecipitated DNA was then resuspended in 20 μl of

water for library preparation. ChIP-seq libraries were prepared according to instructions of the NEBNext Ultra™ DNA Library Prep Kit for Illumina (NEB, Cat. No. E7645). Sequencing was performed at paired-end 150 bp on the Illumina NovaSeq 6000 system according to manufacturer's instructions. For ChIP-qPCR, the precipitated genomic DNA was resuspended in 50 μl of DNA Elution buffer and diluted to 120 μl. ChIP DNA was then analyzed by qPCR using specific primers, and the data were normalized to the input DNA. The results were derived from three independent experiments. Primers used in this study are listed in Table S12.

m^6A sequencing and m^6A-qPCR

Total RNA was isolated and purified using TRIzol reagent (Invitrogen, Carlsbad, CA, USA) following the manufacturer's procedure. The RNA amount and purity of each sample was quantified using NanoDrop ND-1000 (NanoDrop, Wilmington, DE, USA). The RNA integrity was assessed by Bioanalyzer 2100 (Agilent, CA, USA) with RIN number >7.0, and confirmed by electrophoresis with denaturing agarose gel. Approximately more than 1μg of total RNA was fragmented into small pieces using Magnesium RNA Fragmentation Module (NEB, cat. e6150, USA) at 94°C for 5 min. The cleaved RNA fragments (including rRNA fragments) were then incubated with m^6A antibody-dynabead compounds. The Input RNA and IP RNA were reverse-transcribed to create the first-strand cDNA by SMARTScribe™ Reverse Transcriptase (CloneTech, cat.634414, Japan), which were used to perform m^6A-qPCR. Primers used in this study are listed in Table S13. The above first-strand cDNA was attached with adapter and synthesized second-stranded DNAs with PCR by the following conditions: initial denaturation at 94°C for 1 min; denaturation at 98°C for 15 sec, annealing at 55°C for 15 sec, and extension at 68°C for 30 sec; and then final extension at 68°C for 2 min,5 cycles. The amplified DNA-seq library was purified by immobilization onto pure beads. The cDNA sequences originating from rRNA reverse transcription were cut by ZapR v2 and R-Probes v2 (for mammal) under the condition: incubation at 72°C for 2 min,4°C for 2 min,37°C for 1h, 72°C for 10 min, finally stored at 4°C. The final library was amplified by second round PCR which was consistent with the first round PCR program (PCR cycles: 13). Finally, the 2×150bp paired-end sequencing (PE150) was performed on an illumina Novaseq™ 6000 (LC-Bio Technology CO., Ltd., Hangzhou, China) following the vendor's recommended protocol.

Sequencing analysis

RNA-seq Analysis

The sequenced reads were aligned to the mouse reference genome (GRCm39) with an annotated gene model (M29; GENCODE, www.gencodegenes.org/) using Spliced Transcripts Alignment to a Reference (STAR; version 2.7.10a; https://github.com/alexdobin/STAR) with default parameters. The expression abundances of all genes were estimated by running RSEM (version 1.3.1; deweylab.github.io/RSEM/) across all of the samples. The differential expression analysis was performed using edgeR (version 3.38.1; bioconductor.org/packages/release/bioc/html/edgeR.html) with absolute logarithm of fold change (log2fc) cutoff of 0.5849 and P value <0.05. The identified differentially expressed genes isolated from the VSMCs of conditional knockout and control mice were further annotated with gene ontology (GO) and the Kyoto encyclopedia of genes and genomes (KEGG), using the DAVID (Database for Annotation, Visualization and Integrated Discovery) Bioinformatics Tool (version 2021; david.ncifcrf.gov/).

YY1 ChIP-seq Analysis

The sequenced reads of YY1 ChIP-seq were aligned to the human reference genome (GRCh38) with the annotated gene model (release 42; GENCODE) using the Burrow-Wheeler Aligner (version 0.7.17) with the Maximum Exact Match algorithm. After filtering the GRCh38 ChIP-seq blacklisted regions and removal of duplicate reads, YY1 binding peaks were identified using the Model-Based Analysis of ChIP-Seq peak caller (MACS; version 2.2.8; pypi.org/project/MACS2/) with q value cutoff of 0.01. Annotation of peaks was performed with the Hypergeometric Optimization of Motif Enrichment (HOMER) software (version 4.11.1 ; homer.ucsd.edu/homer/) and the annotated gene model (release 42; GENCODE). With default settings, YY1 binding peaks were assigned to the nearest transcription start site (TSS) of genes, and further classified using the following features: TSS (−1 kb to +100 bp), transcription end site (−100 bp to +1kb), exons, introns, and intergenic regions. De novo motif discovery was performed with all the identified YY1 binding peaks by HOMER. Heat map visualization of YY1 ChIP-seq data was performed using deepTools (version 3.5.1; github.com/deeptools/deepTools). Selected genes with YY1 binding peaks were visualized using the UCSC (University of California, Santa Cruz) genome browser.

Histone ChIP-seq Analysis

Raw data of histone ChIP-seq targeting H3K4me3 in human smooth muscle cells were retrieved from ENCODE with an accession code GSE96206. The sequenced reads were aligned and potential YY1 gene targets with DNA regions enriched for histone modification marks were identified as described in the previous paragraph. The BEDtools intersect program was used to evaluate regions overlapped by YY1 and the histone modification marks. Enrichment profiles of selected genes were visualized using the UCSC genome browser.

m^6A-seq data analysis

The sequenced reads were aligned to the mouse reference genome (GRCm39) with an annotated gene model (M29; GENCODE, www.gencodegenes.org/) using Hierarchical Indexing for Spliced Alignment of Transcripts (HISAT2; version 2.2.1; https://daehwankimlab.github.io/hisat2/). m^6A peaks were identified using the R package exomePeak (version 2.16.0; github.com/ZW-xjtlu/exomePeak) with parameters WINDOW_WIDTH=100 and SLIDING_STEP=15. Motifs were identified by HOMER (version 4.11.1 ; homer.ucsd.edu/homer/) with parameter Motif length=7. The distribution of m^6A peaks over different regions on the transcripts was generated by the R package Guitar (v2.12.0). The m^6A peaks of selected genes were visualized using the UCSC (University of California, Santa Cruz) genome browser.

Wire myograph

Vasoreactivity was measured in wire myograph as previously described[22]. In aortas or mesenteric vessels with endothelium, L-NAME (100 μmol/L, 30 minutes) was used before testing vasoconstriction and vasodilation. In some experiments, the endothelial layer of the aortas was mechanically removed. Vessels were dissected in oxygenated ice-cold Krebs solution that contained (mmol/L): 119 NaCl, 4.7 KCl, 2.5 $CaCl_2$, 1 $MgCl_2$, 25 $NaHCO_3$, 1.2 KH_2PO_4 and 11 D-glucose. Measurements of isometric tension were recorded in wire myograph (Danish Myo Technology) with chambers connected to 95% O_2 and 5% CO_2 for pH control. The aortic segments were stretched to optimal baseline tension at 3 mN. After that, they were washed in Krebs solution for three times and allowed to equilibrate for 15 minutes before contracted with 60 mmol/L KCl to test viability. VSMC-dependent vasoconstriction induced by testing concentration-responses to cumulative addition of phenylephrine (Phe, μmol/L). VSMC-dependent relaxation was measured by testing concentration-responses to

cumulative concentration of acetylcholine (Ach) or sodium nitroprusside (SNP). The maximum response (Rmax) and logEC50 values were obtained from the fitted concentration-response curves using non-linear regression (Curve Fit) in Prism, and compared using Student's *t*-test.

Measurement of intracellular calcium influx by confocal microscopy

Calcium influx into the VSMC layer of an aorta was measured by confocal microscopy as previously described[26]. The aorta was opened longitudinally, and the vascular strip was loaded with 10 μmol/L calcium indicator fluo-4 AM (Thermo Fisher, F14201) diluted in HBSS solution. Fluorimetric measurements were performed using a laser scanning confocal system. Flourescence was triggered at excitation 495 nm with emission filter 505-525 nm. Changes in intracellular calcium concentration were expressed as F1/F0 ratios where F1 was the fluorescence intensity at a specific time while F0 measured at the starting point of image recording. Briefly, calcium influx was triggered by 10 μmol/L Phe or A23187. Continuous recording of fluorescence images were obtained every 15 second.

Flow cytometry

The medial layer of the aorta was surgically isolated after digestion with enzyme solution containing collagenase II (1 mg/ml) and Pepsin (1mg/ml) at 37°C for 15min. The medial layers were then cut into pieces and digested in a cocktail containing collagenase I (450 U/ml) and collagenase XI (125 U/ml) at 37°C with occasional agitation until the tissues were fully digested. The isolated VSMCs were cultured with vascular cell basal medium containing 15% FBS and a combination of penicillin–streptomycin at 37°C in an incubator. Red blood cells were first removed with lysis buffer (eBiosciences) at room temperature for 5 min. The dissociated mouse aortic tissues were then washed three times with 2% FBS-containing PBS and analyzed on the flow cytometer (BD FACSAria™ Fusion). Propidium iodide (PI, BD Pharmingen) positive dead cells were excluded from live cell analysis/sorting; and FACS data was analyzed by the FlowJo software (Tree star).

Histological staining

The aortas were fixed by 4% PFA at 4℃ overnight. After fixation, the tissues were washed with 1× PBS several times and dehydrated in 30% sucrose at 4℃ until the tissues sank to the bottom. The dehydrated tissues were embedded in Optimum Cutting Temperature (OCT)

compound (Sakura, 4583) and allowed to freeze at -80℃. Before cryosection, the tissue blocks were put in the cryosection machine (Thermo fisher, CryoStar NX70) for 20-30 min. 8-10 μm sections were collected. Before staining, the slides were washed with 1× PBS to remove residual OCT. The sections were stained with H&E (Beyotime, C0105S), and Masson's Trichrome staining (Epredia, 87019) according to manufacturers' instructions. Images of the stained sections were captured by Leica DM4 B microscopes (Leica Microsystems Inc) and analyzed by ImageJ software.

Immunostaining

The aortas were fixed by 4% PFA at 4℃ overnight. After fixation, the tissues were washed with 1× PBS several times and dehydrated in 30% sucrose at 4℃ until the tissues sank to the bottom. The dehydrated tissues were embedded in Optimum Cutting Temperature (OCT) compound (Sakura, 4583) and allowed to freeze at -80℃. Before cryosection, the tissue blocks were put in the cryosection machine (Thermo fisher, CryoStar NX70) for 20-30 min. 8-10 μm sections were collected. Before staining, the slides were washed with 1× PBS to remove OCT, and the tissues were permeabilized by 1× PBS with 0.3% Triton X-100 (RPI, T18000-0.05) for 10-15 min at room temperature, followed by blocking with 1% BSA (Beyotime, ST023), 22.52 mg/mL glycine (Invitrogen, 15527013), 5% donkey serum (Sigma-Aldrich, S30) or goat serum (Sigma-Aldrich, G9023) in 1× PBST (1× PBS + 0.1% Tween 20) for 30-60 min at room temperature. After blocking, the tissues were incubated with primary antibodies diluted in 1% BSA in 1× PBST at 4℃ overnight in the dark. On the following day, the tissues were washed with 1× PBST 3-5 times, followed by incubation with secondary antibodies diluted in 1% BSA in 1× PBST at room temperature for 30-45 min in the dark. The tissues were washed with 1× PBST 3-5 times, then incubated with 0.1-1 μg/mL Hoechst 33342 (Life technology, H3570) for 5-10 min at room temperature in the dark. The slides were then washed with 1× PBST 3-5 times and mounted with the mounting medium (Abcam, ab104135). Fluorescence images were acquired by fluorescence microscope (Leica) or confocal microscope (Leica). Primary antibodies used in this study: mouse anti-human/mouse α-SMA (1:400 dilution, Dako, M085101-2), rabbit anti-human MYH11 antibody (1:100 dilution, abcam, ab125884) and rabbit anti-mouse cleaved caspase 3 (1:100 dilution, CST, #9661). Secondary antibodies used: Goat anti-rabbit IgG (H+L) 546 (1:1000 dilution, Invitrogen, A-11010), and Goat anti-mouse IgG (H+L) 488 (1:1000 dilution, Invitrogen, A-11001).

Dual-luciferase reporter assay

Luciferase activities of promoters were performed via Dual-Luciferase Reporter Assay System (Promega Corporation). hESC-SMC cells were transfected with 500 ng each METTL3 wild-type (WT) or METTL3 deletion luciferase reporter vector (a deletion vector was constructed by deleting YY1 binding sites ATGGA), pRL-TK (Promega Corporation), and YY1 overexpression lentivirus (OE-YY1) in 24-well culture plate. Transfection plasmid combination was as follows: 1) OE-NC + pGL3-Basic + pRL-TK; 2) OE-YY1 + pGL3-Basic + pRL-TK; 3) OE-NC + METTL3-WT + pRL-TK; 4) OE-YY1 + METTL3-WT + pRL-TK; 5) OE-NC + METTL3-Del1 + pRL-TK (Deleted 2 binding sites); 6) OE-YY1 + METTL3-Del1 + pRL-TK (Deleted 2 binding sites); 7) OE-NC + METTL3-Del2 + pRL-TK (Deleted 1 binding site); 8) OE-YY1 + METTL3-Del2 + pRL-TK (Deleted 1 binding site); 9) OE-NC + METTL3-Del3 + pRL-TK (Deleted 1 binding site); 10) OE-YY1 + METTL3-Del3 + pRL-TK (Deleted 1 binding site). Total protein was prepared at 48 h post-transfection. Lysate cells were collected for centrifugation at 10 000 g for 5 min, and supernatant was taken as test solution and then operated according to the instructions of Dual-Luciferase® Reporter Assay System (Promega). An amount of 50 µl of each sample was used to determine luciferase activity. The pRL-TK plasmid was used as a normalizing control.

RNA immunoprecipitation analysis (RIP)

An RNA Immunoprecipitation Kit (BersinBio, China) was performed for RIP analysis according to the manufacturer's instructions. First, the mouse aorta was minced and grounded with a grinding rod for 1 min by adding liquid nitrogen and polysome lysis buffer supplemented with protease inhibitor and RNase inhibitor. The aorta samples were divided into 3 parts according to 0.8 mL (IP), 0.8 mL (IgG), and 0.1 mL (Input). IP and IgG samples were added with the corresponding experimental antibodies, rabbit anit-mouse IGF2BP1 (1:100 dilution, CST, #8482) and mouse anti-mouse IgG (Millipore, #12-371), respectively, and were incubated at 4 °C for overnight. Samples were then incubated with balanced protein A/G beads for 2 h, followed by RNA eluting using polysome washing buffer, polysome elution buffer, and proteinase K. Finally, RNA was extracted with TRIzol reagent, and cDNA was generated with iScript™ cDNA Synthesis Kit (Bio-Rad, USA), which was analyzed using qRT-PCR according to the percentage input method according to the manufacturers' instructions. The primers used for RIP-qPCR in this study are listed in Table S14.

Co-immunoprecipitation (co-IP)

For the co-IP assay, cells and mouse aorta tissue were lysed in RIPA buffer (Beyotime) supplemented with proteinase inhibitor (Beyotime) and dithiothreitol (DTT). The mouse aortic tissues were minced and grounded with a grinding rod for 1 min by adding liquid nitrogen and 200 μl IP buffer, and then incubated on ice for 30 min. The lysate was harvested by centrifugation at 13,200g at 4°C for 3 min. In some experiments, the lysate was incubated with Dnase I (0.8 U/mL) for 15 minutes. The precleaning step was followed by incubation with rabbit anti-mouse YY1 (1:100 dilution, Active motif, #61779), rabbit anti-mouse SET1A (1:100 dilution, CST, #61702S) or mouse anti-mouse IgG antibody (1:1000 dilution, Millipore, #12-371) with rotation at 4 °C overnight and Protein A/G magnetic beads (ThermoFisher) with rotation at 4 °C 2 h. The immune complex was then washed with RIPA buffer for 3 times (5 min/time). Bound proteins were eluted with SDS-PAGE sample loading buffer (Beyotime) by incubating at 95°C for 8 min followed by Western blot analysis.

Western blot analysis

Cells and aorta tissue were collected at the indicated times in RIPA buffer (Beyotime). The aorta tissues were minced and grounded with a grinding rod for 1 min by adding liquid nitrogen and 200 μl RIPA buffer, and then incubated on ice for 30 min. The lysate was harvested by centrifugation at 13,200g at 4°C for 3 min. The lysed protein was then added to the loading buffer (Beyotime), and incubated at 95°C for 8 min. Equal amounts of proteins were separated by SDS-PAGE and transferred to PVDF western blotting membranes (Roche). The membrane was soaked in 5% BSA in TBST for 1 hour at room temperature and incubated with primary antibodies overnight at 4 °C. Anti-mouse or anti-rabbit horseradish peroxidase (HRP)-conjugated secondary antibodies (Santa Cruz,1:5000) were used. β-actin was used as an internal control. Primary antibodies: rabbit anti-human/mouse YY1 (1:1000 dilution, Abcam, ab109237), mouse anti-human/mouse MLCK (1:1000 dilution, Sigma, SAB1300116), mouse anti-human/mouse TGFβ2 (1:100 dilution, Santacruz, sc-374658), mouse anti-mouse Smad3 (1:100 dilution, Santacruz, sc-101154), mouse anti-mouse p-Smad3 (1:100 dilution, santacruz, sc-517575), rabbit anti-mouse SM22α (1:1000 dilution, CST, #52011), rabbit anti-mouse calmodulin (1:1000 dilution, Abcam, ab45689), rabbit anti-mouse KLF4 (1:100 dilution, Active motif, #39745), mouse anti-human/mouse α-SMA (1:1000 dilution, Dako, M085101-2), rabbit anti-mouse/human METTL3 (1:1000 dilution, abcam, ab195352), rabbit anti-mouse RLC (1:1000 dilution, CST, #8505), rabbit anti-mouse p-RLC (1:1000 dilution, CST, #95777),

mouse anti-human/mouse β-actin (1:5000 dilution, GenScript, A00702), rabbit anti-human/mouse MYH11 (1:1000 dilution, Abcam, ab125884), rabbit anti-human/mouse SET1A (1:1000 dilution, CST, #61702S), rabbit anti-human/mouse SET1B (1:1000 dilution, CST, #44922), rabbit anti-mouse MLL1 (D2M7U, 1:1000 dilution, CST, #14689), rabbit anti-mouse MLL1 (D6G8N, 1:1000 dilution, CST, #14197), rabbit anti-mouse MLL2 (1:1000 dilution, CST, #63735), rabbit anti-mouse WDR5 (1:1000 dilution, CST, #13105), rabbit anti-mouse WDR82 (1:1000 dilution, CST, #99715), rabbit anti-mouse Menin (1:1000 dilution, CST, #6891), mouse-anti-mouse RNA pol II (1:1000, Active Motif, #39097), rabbit anti-human/mouse H3K4me3 (1:1000 dilution, abcam, ab8580), rabbit anti-human/mouse H3K4me2 (1:1000 dilution, CST, #9725), rabbit anti-human/mouse H3K27ac (1:1000 dilution, abcam, ab4729), and rabbit anti-human/mouse H3K27me3 (1:1000 dilution, Millipore, #07-449).

Quantification of m^{6}A mRNA methylation

Total RNA from aorta tissues was extracted using TRIzol reagent (Vazyme, China) and the mRNA was purified by Dynabeads ™ mRNA purification Kit (Therom Fish) according to the manufacturer's instructions. Total RNA was heated at 65 °C for 2 min to disrupt secondary structures and then placed on ice. Total RNA was transferred to the resuspended Dynabeads ™ magnetic beads in 1.5 mL EP tubes, that were subsequently placed on the magnet for 30s to remove the supernatant and washed 1 time with binding buffer. 100 µl binding buffer was added to resuspend Dynabeads ™ magnetic beads and 10 µg total RNA was added to the Dynabeads ™ magnetic beads at room temperature for 5 min. mRNA-bead complex was washed twice with washing buffer B, and 20 µl ddH_2O was added to the mRNA-bead complex followed by heating at 72 °C for 2 min. mRNA was transferred to a new tube. m^{6}A mRNA methylation was detected by the EpiQuik™ m^{6}A RNA Methylation Quantification Kit (EpiQuik, P-9005) according to the manufacturer's instructions. Firstly, 80 µl of binding solution was added to each well, followed by 2 µl NC (Negative control), 2 µl PC (Positive control), with sample mRNA (control and CKO-YY1 or CKO-Mettl3) into the wells that was incubated at 37 °C for 90 min. The binding solution was removed and samples were washed for three times with 150 µl Diluted WB (wash buffer). After that, 50 µl Diluted CA (Capture antibody), 50 µl Diluted DA (Detection antibody) and 50 µl Diluted ES (Enhancer solution) were added to the wells and allowed to incubate at room temperature for 60 min, 30 min, and 30 min. Samples were washed with 150 µl Diluted WB. Finally, 50 µl DS (Developer solution)

was added to each well followed by incubation at room temperature for 2 min. 50 µl SS (stop solution) was then added to stop enzyme reaction. The absorbance was detected on a microplate reader at 450 nm and the relative m^6A mRNA methylation was calculated.

RNA stability test

hESC-VSMCs were seeded at a density of 10^6 cells/ml and treated with 20 pmol/mL *Mettl3*, YY1 or negative control siRNA for 24 hours. After that, cells were treated with 5 ug/mL actinomycin D (Life Technologies, Cat No. 11805017) for the indicated time points to inhibit transcription. Cells were harvested for RNA extraction and the concentration of total RNA was determined using NanoDrop 2000 (ThermoFisher). Equal amounts of total RNA for each sample were reverse transcribed using iScript cDNA Synthesis Kit (BioRad, 170-8891) followed by RT-PCR as aforementioned.
